# Supplementary material for: Synthesis of New Azetidine and Oxetane Amino Acid Derivatives through Aza-Michael Addition of NH-Heterocycles with Methyl 2-(Azetidin- or Oxetan-3-Ylidene)Acetates
Source: Molecules. 2023 Jan 21;28(3):1091. doi: 10.3390/molecules28031091 (PMC9921373; doi:10.3390/molecules28031091)
Supplement: Supplementary file 1 [file molecules-28-01091-s001.zip › molecules-2134144-supplementary.pdf]

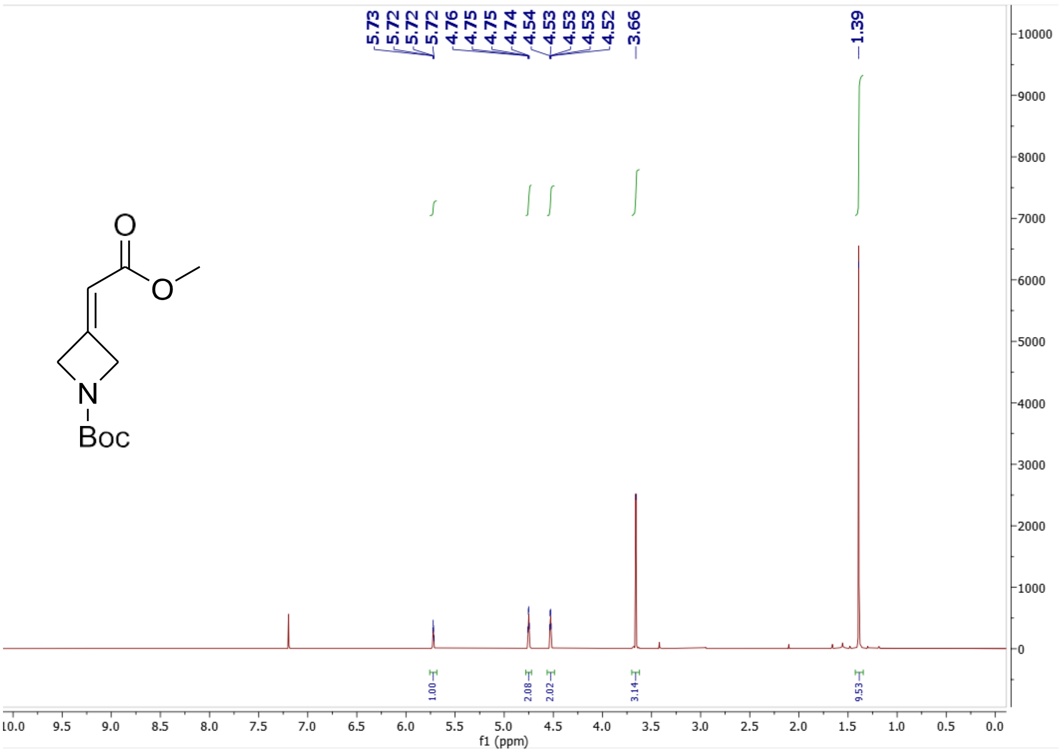

Figure S1. *tert*-Butyl 3-(2-methoxy-2-oxoethylidene)azetidine-1-carboxylate (3) ^{1}H NMR spectrum (700 MHz, $CDCl_3$).

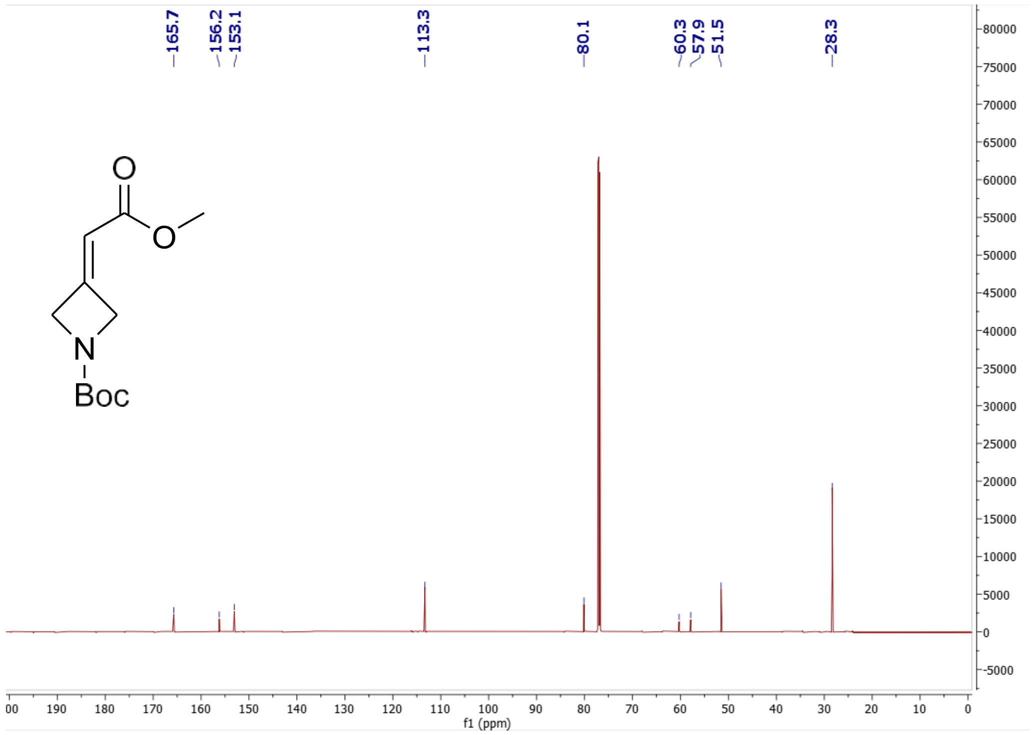

Figure S2. *tert*-Butyl 3-(2-methoxy-2-oxoethylidene)azetidine-1-carboxylate (3) ^{13}C NMR spectrum (176 MHz, $CDCl_3$).

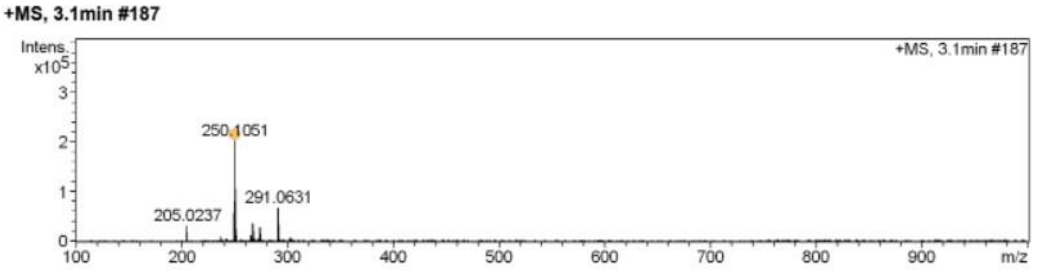

Meas. m/z	#	Ion Formula	m/z	err [ppm]	mSigma	# Sigma	Score	rdb	e⁻ Conf	N-Rule
250.1051	1	C11H17NNaO4	250.1050	-0.6	0.8	1	100.00	3.5	even	ok

Figure S3. *tert*-Butyl 3-(2-methoxy-2-oxoethylidene)azetidine-1-carboxylate (3) HRMS (ESI-TOF).

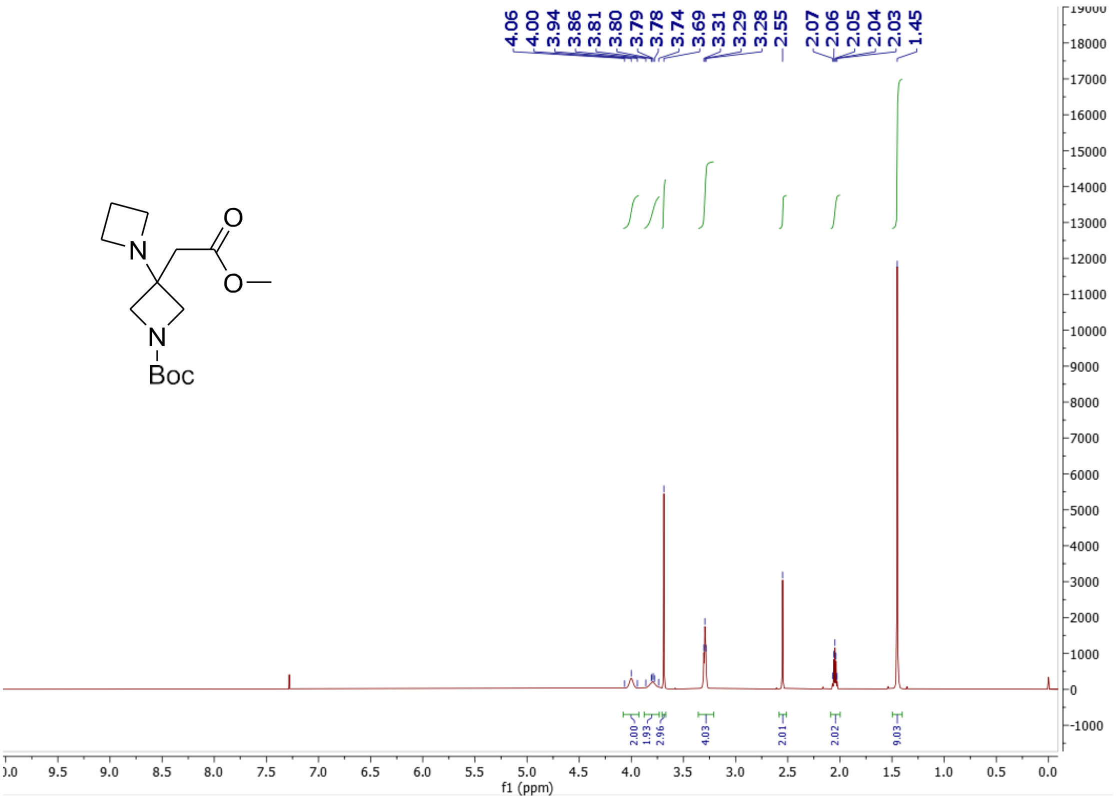

Figure S4. *tert*-Butyl 3'-(2-methoxy-2-oxoethyl)-[1,3'-biazetidine]-1'-carboxylate (4a) ^{1}H NMR spectrum (700 MHz, $CDCl_3$).

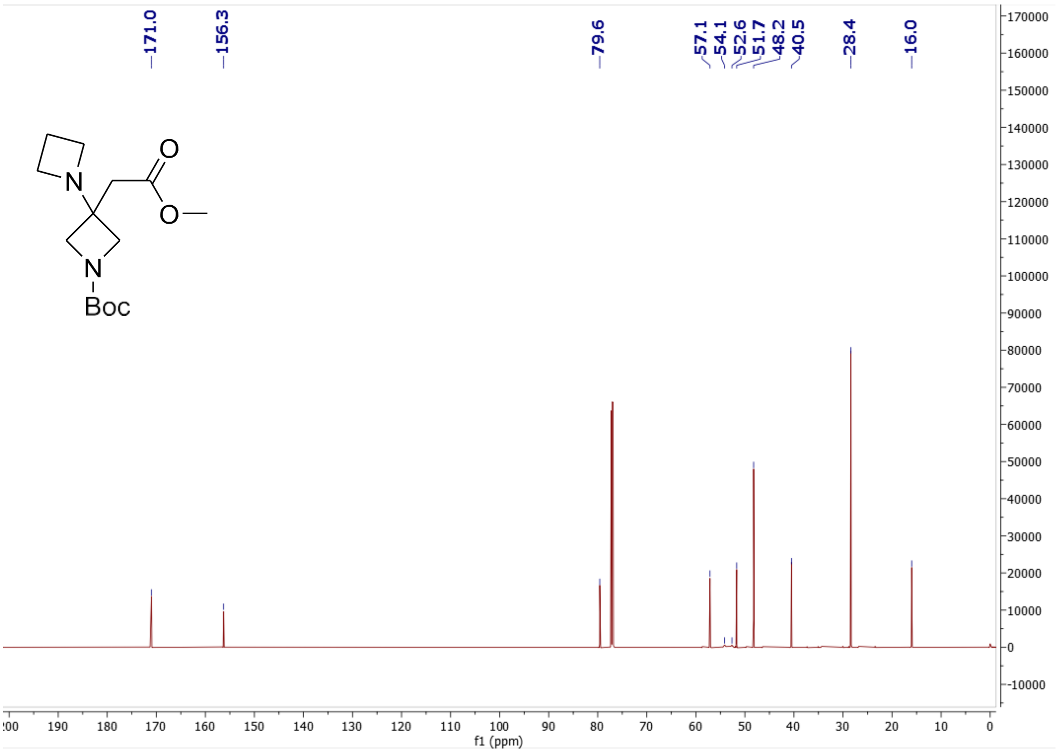

Figure S5. *tert*-Butyl 3'-(2-methoxy-2-oxoethyl)-[1,3'-biazetidine]-1'-carboxylate (4a) ^{13}C NMR spectrum (176 MHz, $CDCl_3$).

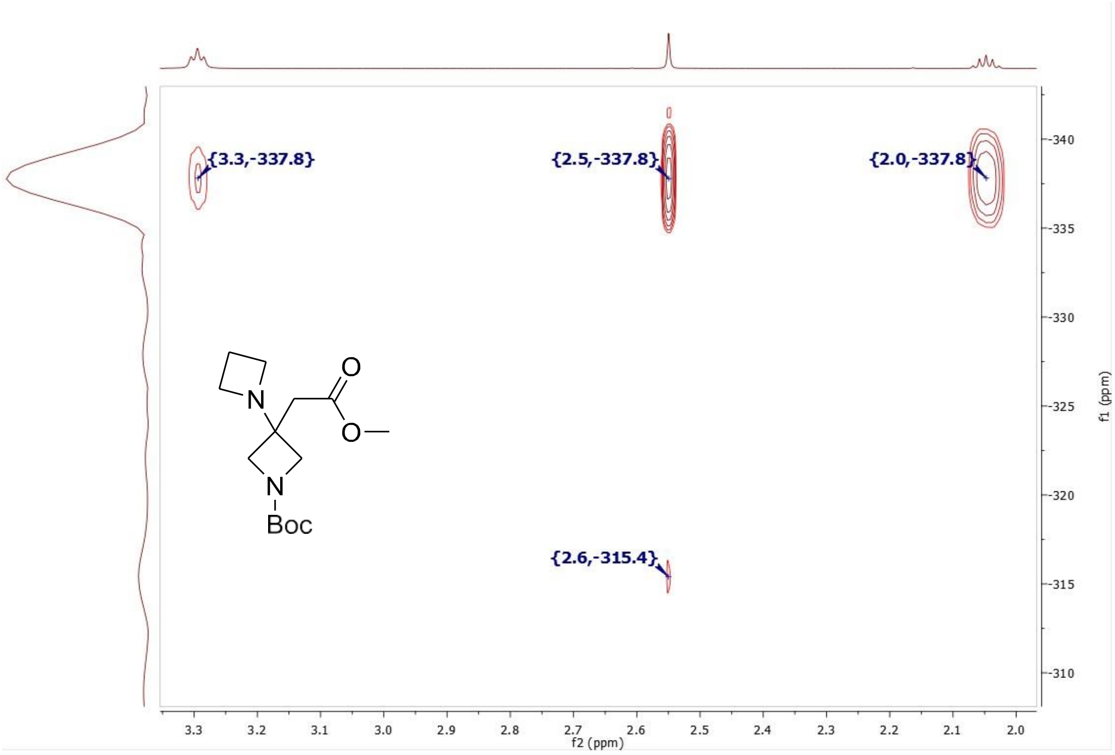

Figure S6. *tert*-Butyl 3'-(2-methoxy-2-oxoethyl)-[1,3'-biazetidine]-1'-carboxylate (4a) ^{1}H-^{15}N HMBC spectrum (71 MHz, $CDCl_3$).

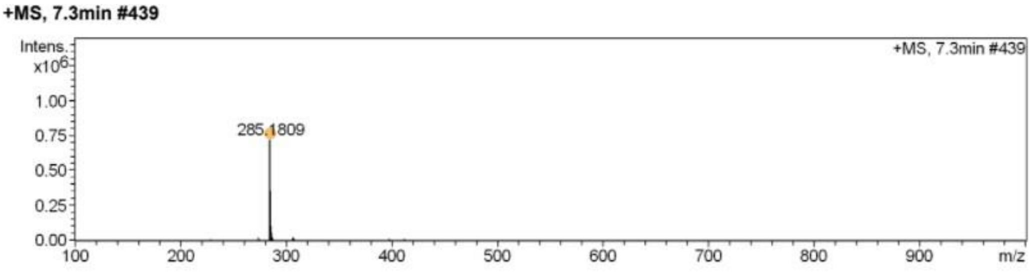

Meas. m/z	#	Ion Formula	m/z	err [ppm]	mSigma	# Sigma	Score	rdb	e⁻ Conf	N-Rule
285.1809	1	C14H25N2O4	285.1809	-0.2	4.3	1	100.00	3.5	even	ok

Figure S7. *tert*-Butyl 3'-(2-methoxy-2-oxoethyl)-[1,3'-biazetidine]-1'-carboxylate (4a) HRMS (ESI-TOF).

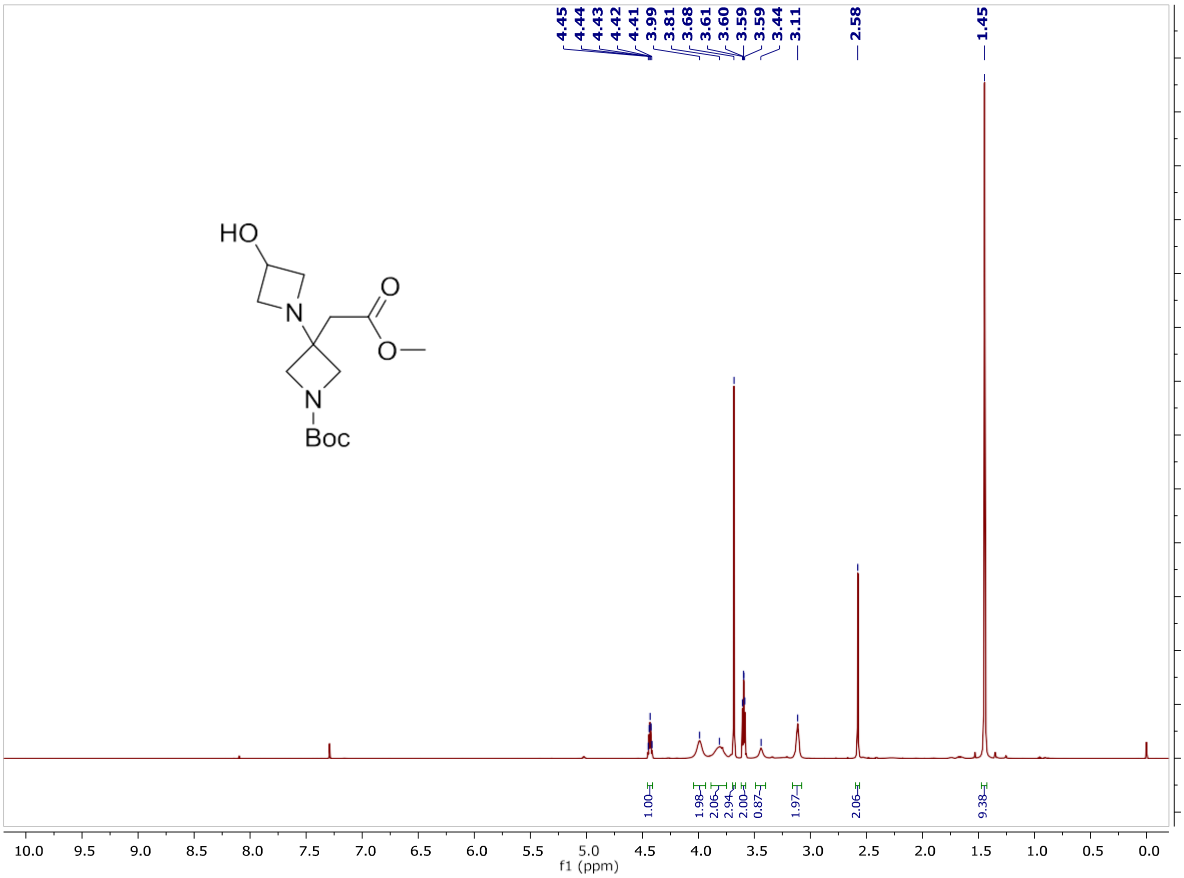

Figure S8. *tert*-Butyl 3-hydroxy-3'-(2-methoxy-2-oxoethyl)[1,3'-biazetidine]-1'-carboxylate (4b) ^{1}H NMR spectrum (700 MHz, $CDCl_3$).

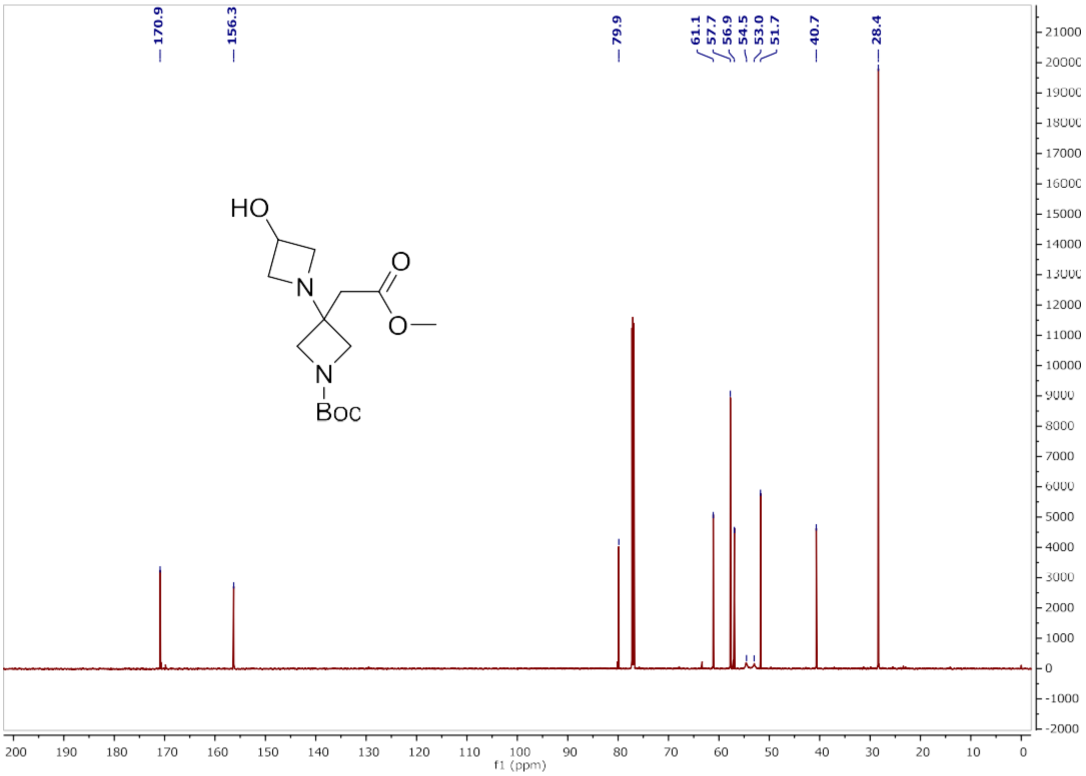

Figure S9. *tert*-Butyl 3-hydroxy-3'-(2-methoxy-2-oxoethyl)[1,3'-biazetidine]-1'-carboxylate (4b) ^{13}C NMR spectrum (176 MHz, $CDCl_3$).

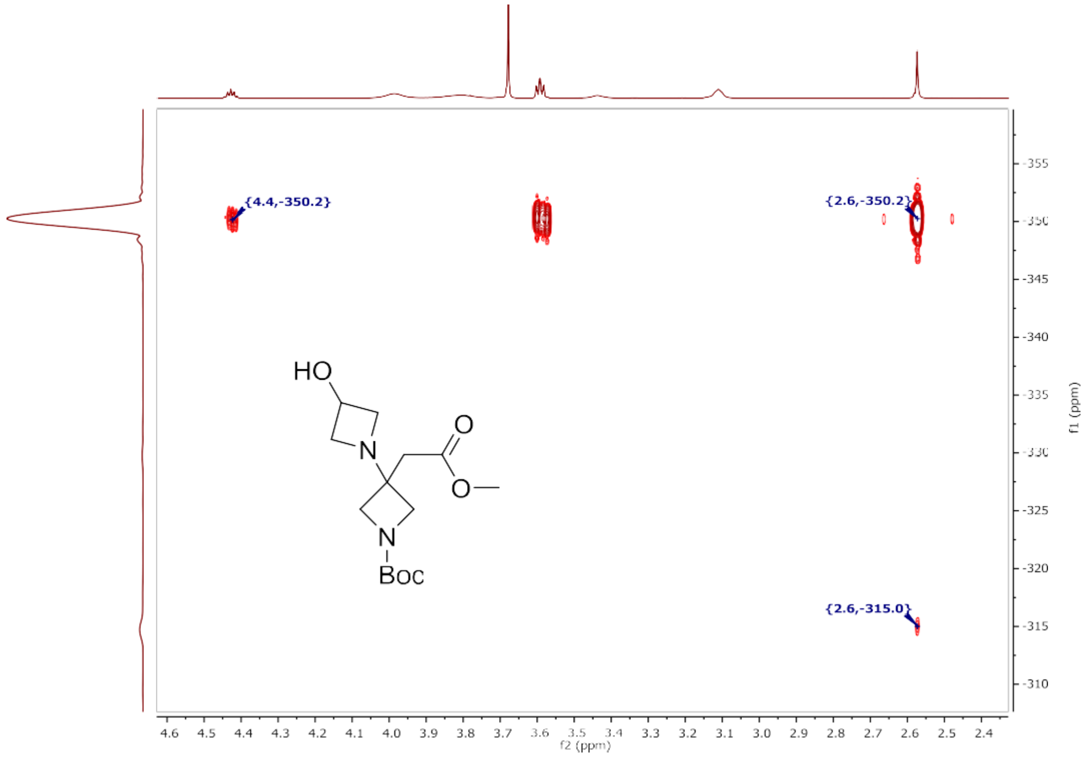

Figure S10. *tert*-Butyl 3-hydroxy-3'-(2-methoxy-2-oxoethyl)[1,3'-biazetidine]-1'-carboxylate (4b) ^{1}H-^{15}N HMBC spectrum (71 MHz, $CDCl_3$).

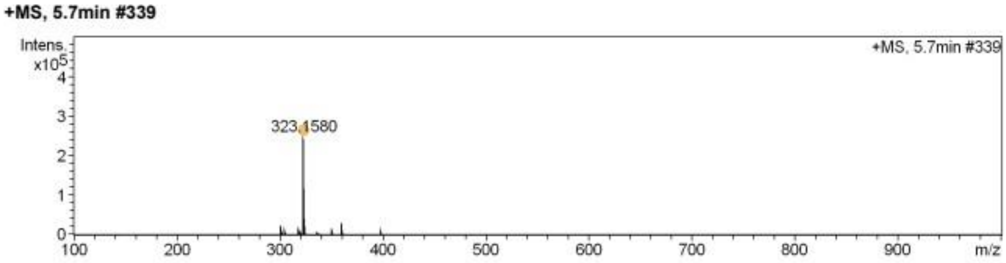

Meas. m/z	#	Ion Formula	m/z	err [ppm]	mSigma	# Sigma	Score	rdb	e¯ Conf	N-Rule
323.1580	1	C14H24N2NaO5	323.1577	-0.7	4.6	1	100.00	3.5	even	ok

Figure S11. *tert*-Butyl 3-hydroxy-3'-(2-methoxy-2-oxoethyl)[1,3'-biazetidine]-1'-carboxylate (4b) HRMS (ESI-TOF).

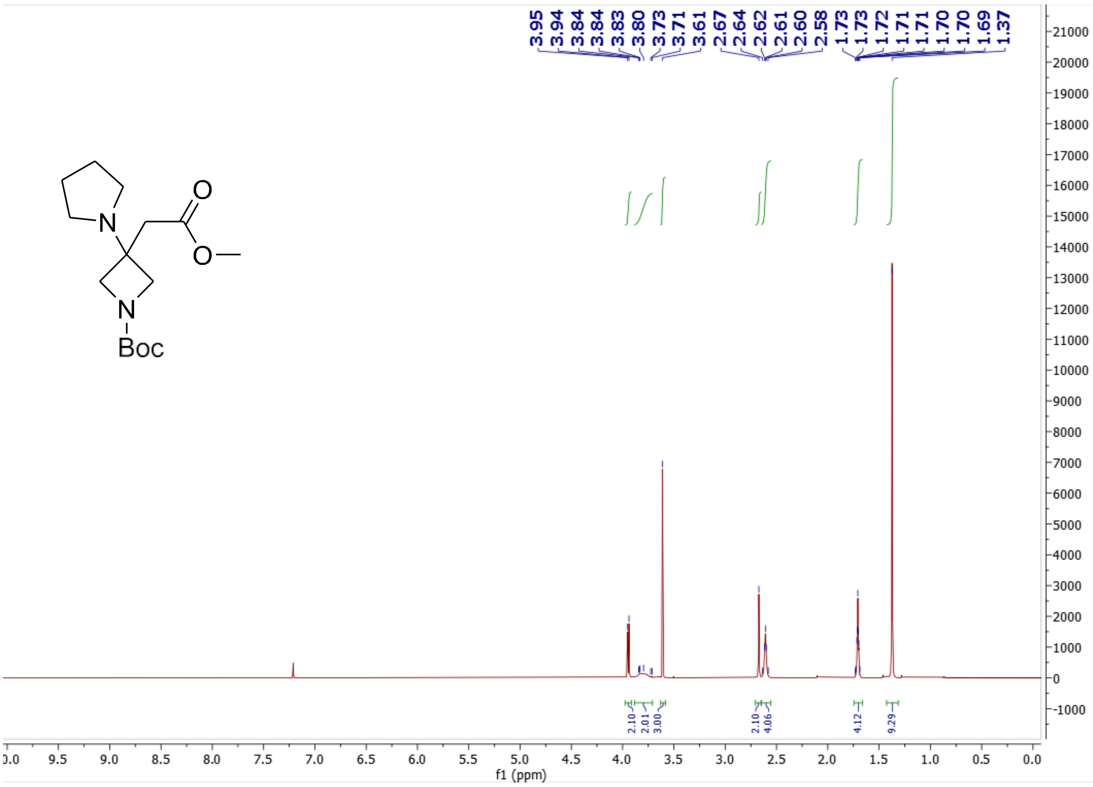

Figure S12. *tert*-Butyl 3-(2-methoxy-2-oxoethyl)-3-(pyrrolidin-1-yl)azetidine-1-carboxylate (4c) ^{1}H NMR spectrum (700 MHz, $CDCl_3$).

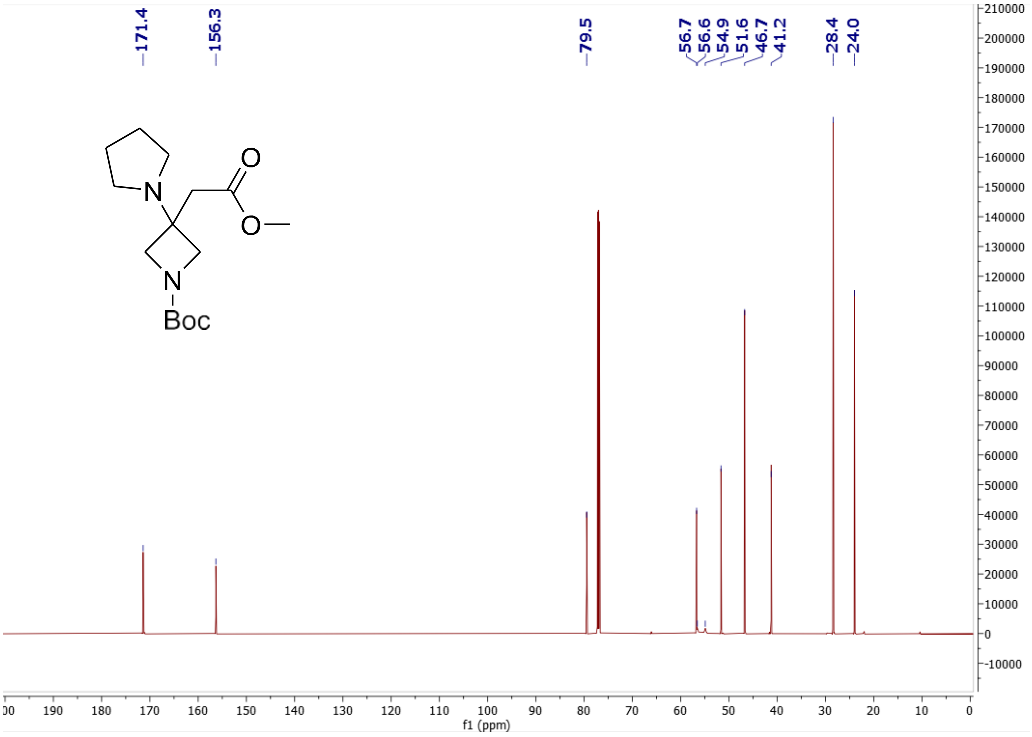

Figure S13. *tert*-Butyl 3-(2-methoxy-2-oxoethyl)-3-(pyrrolidin-1-yl)azetidine-1-carboxylate (4c) ^{13}C NMR spectrum (176 MHz, $CDCl_3$).

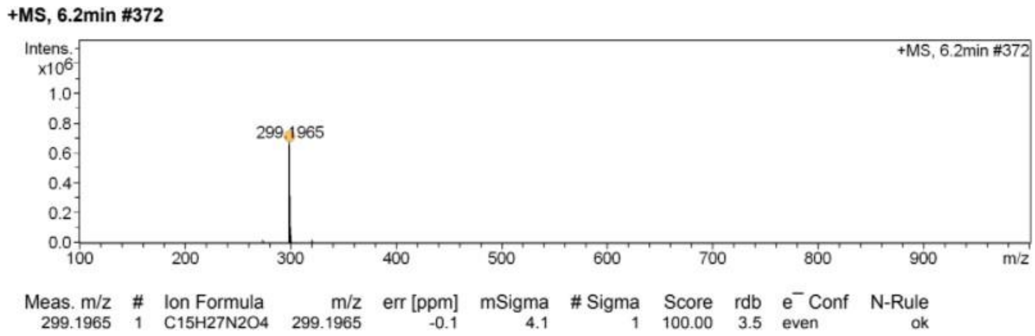

Figure S14. *tert*-Butyl 3-(2-methoxy-2-oxoethyl)-3-(pyrrolidin-1-yl)azetidine-1-carboxylate (4c) HRMS (ESI-TOF).

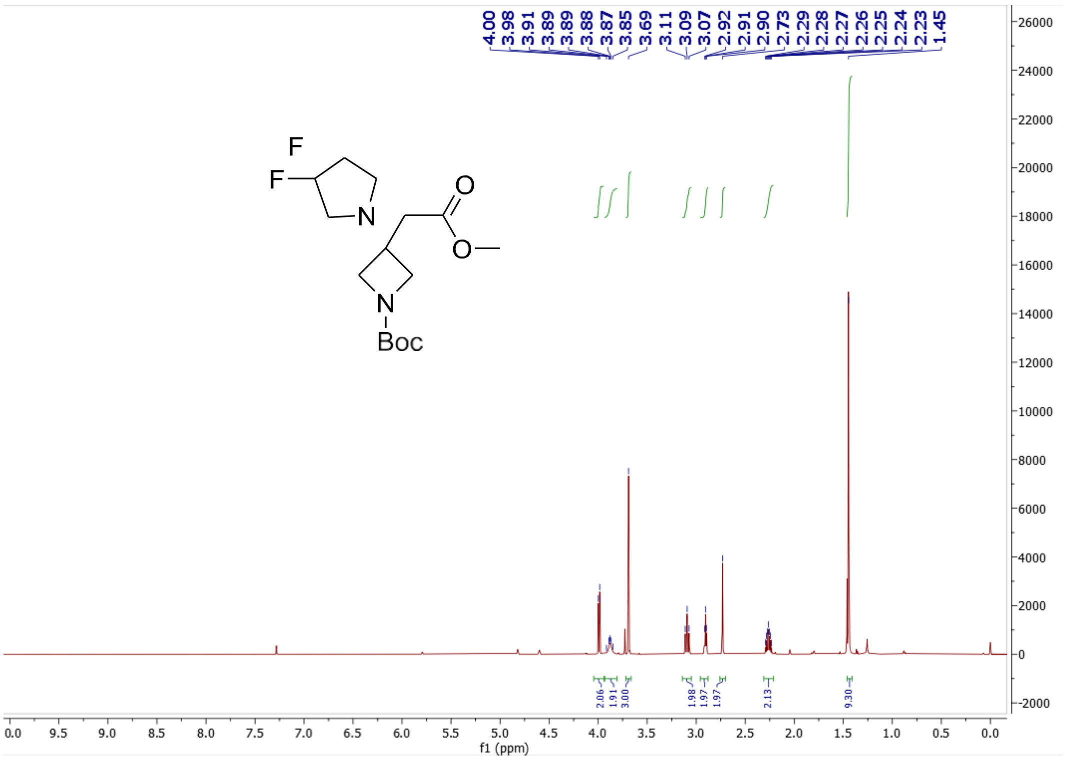

Figure S15. *tert*-Butyl 3-(3,3-difluoropyrrolidin-1-yl)-3-(2-methoxy-2-oxoethyl)azetidine-1- carboxylate (4d) ^{1}H NMR spectrum (700 MHz, $CDCl_3$).

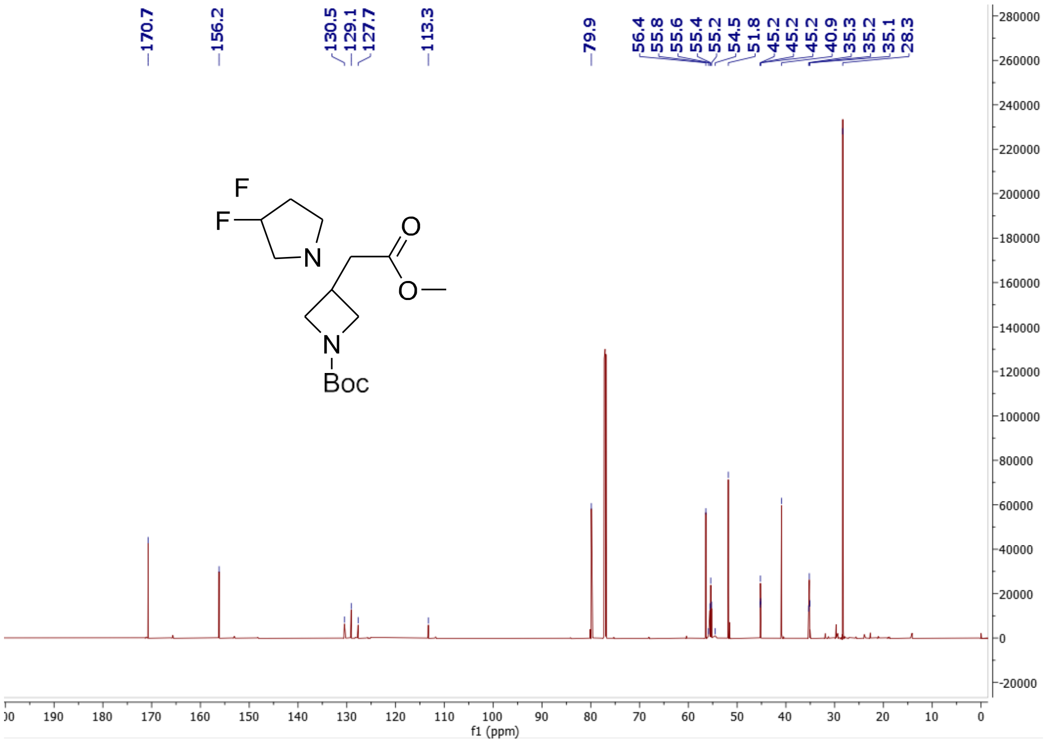

Figure S16. *tert*-Butyl 3-(3,3-difluoropyrrolidin-1-yl)-3-(2-methoxy-2-oxoethyl)azetidine-1- carboxylate (4d) ^{13}C NMR spectrum (176 MHz, $CDCl_3$).

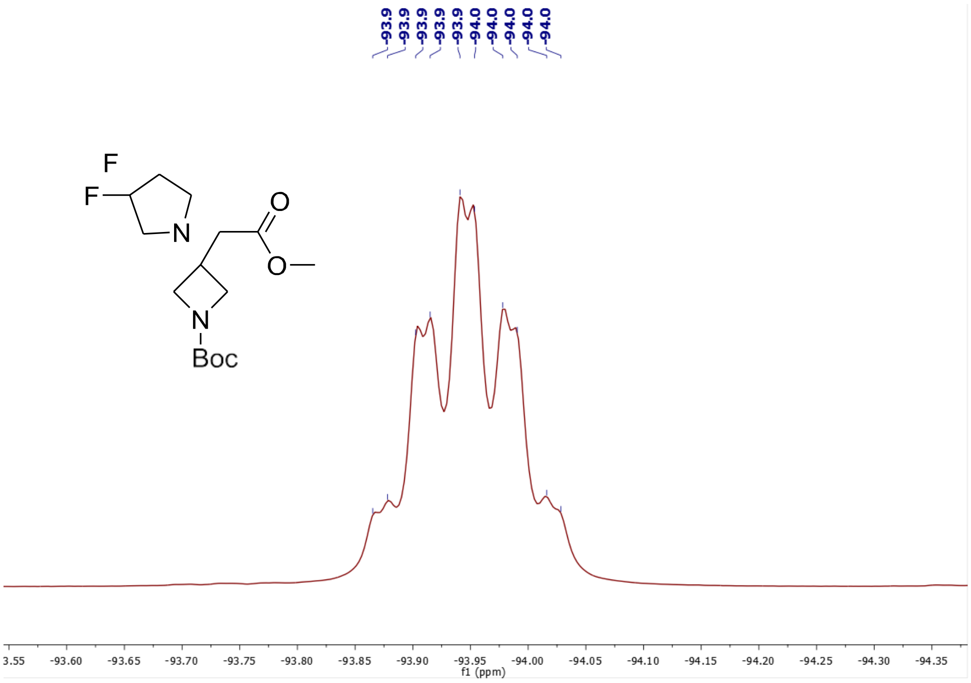

Figure S17. *tert*-Butyl 3-(3,3-difluoropyrrolidin-1-yl)-3-(2-methoxy-2-oxoethyl)azetidine-1- carboxylate (4d) ^{19}F NMR spectrum (376 MHz, $CDCl_3$).

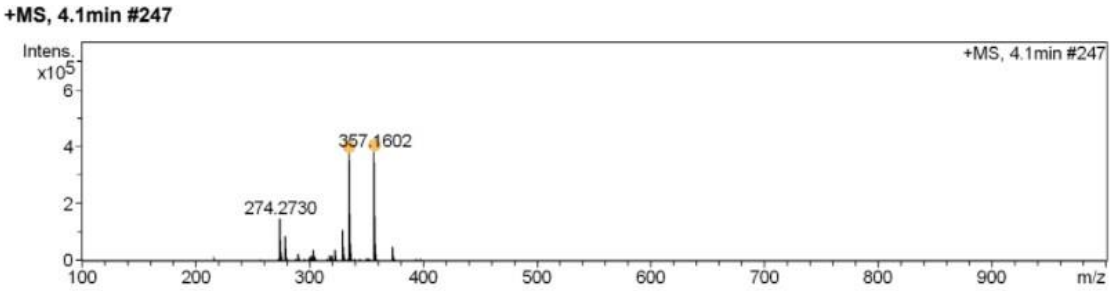

Meas. m/z	#	Ion Formula	m/z	err [ppm]	mSigma	# Sigma	Score	rdb	e⁻ Conf	N-Rule
335.1777	1	C15H25F2N2O4	335.1777	0.1	1.7	1	100.00	3.5	even	ok
357.1602	1	C15H24F2N2NaO4	357.1596	1.6	1.2	1	100.00	3.5	even	ok

Figure S18. *tert*-Butyl 3-(3,3-difluoropyrrolidin-1-yl)-3-(2-methoxy-2-oxoethyl)azetidine-1- carboxylate (4d) HRMS (ESI-TOF).

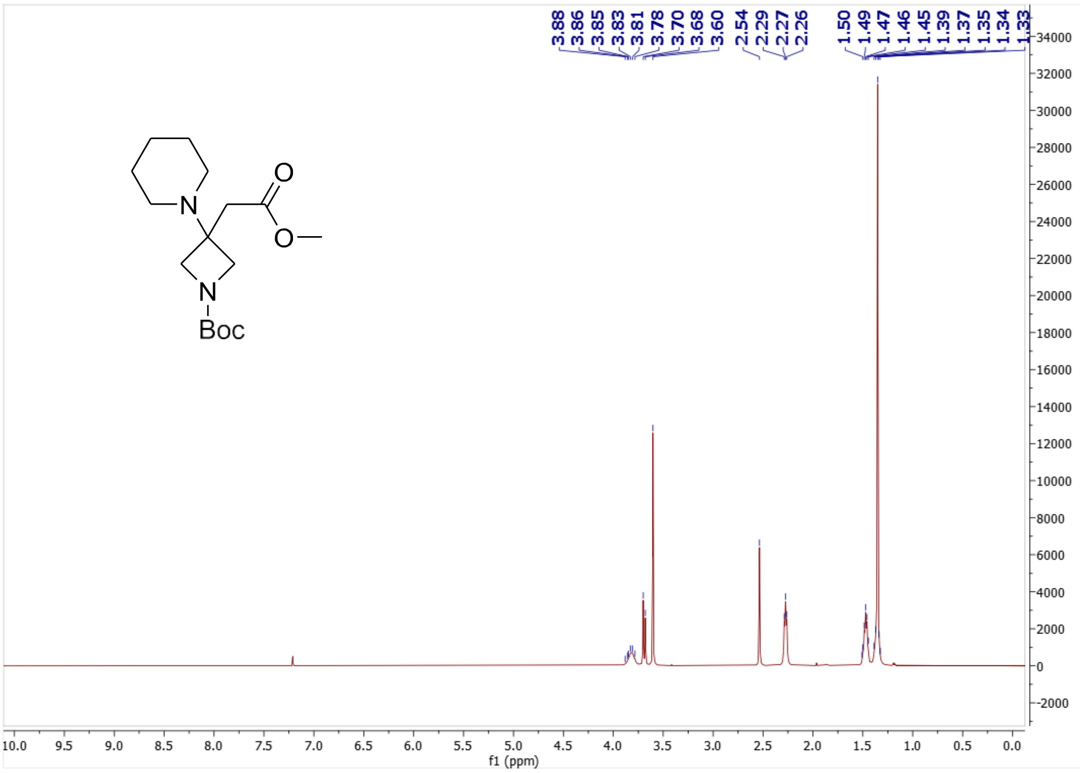

Figure S19. *tert*-Butyl 3-(2-methoxy-2-oxoethyl)-3-(piperidin-1-yl)azetidine-1-carboxylate (4e) ^{1}H NMR spectrum (400 MHz, $CDCl_3$).

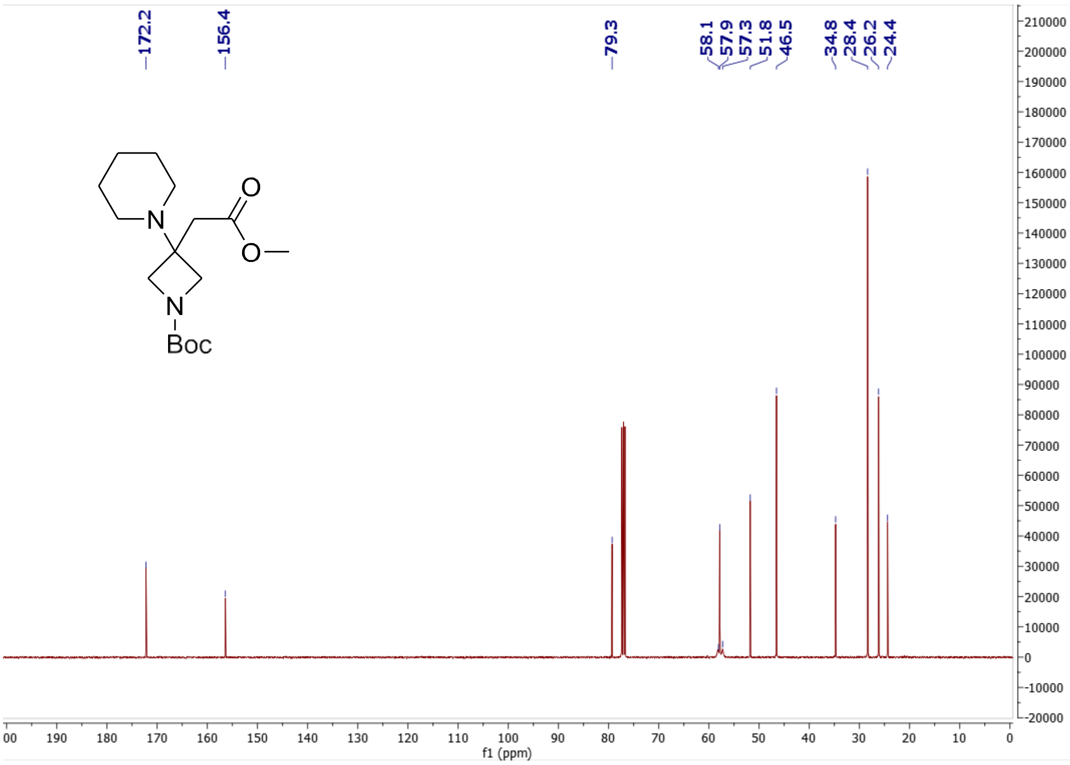

Figure S20. *tert*-Butyl 3-(2-methoxy-2-oxoethyl)-3-(piperidin-1-yl)azetidine-1-carboxylate (4e) ^{13}C NMR spectrum (101 MHz, $CDCl_3$).

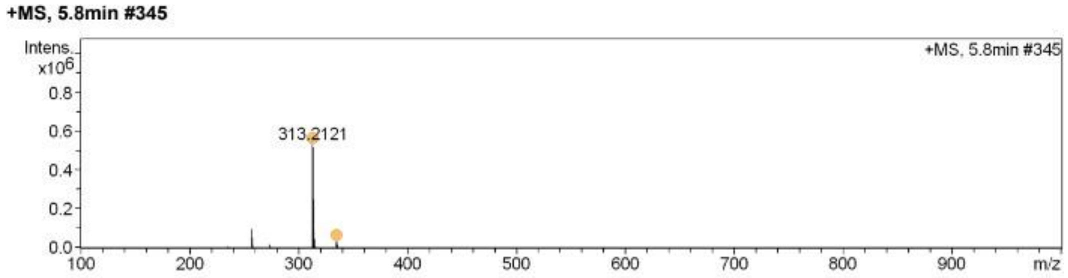

Meas. m/z	#	Ion Formula	m/z	err [ppm]	mSigma	# Sigma	Score	rdb	e⁻ Conf	N-Rule
313.2121	1	C16H29N2O4	313.2122	0.3	0.1	1	100.00	3.5	even	ok
335.1931	1	C16H28N2NaO4	335.1941	-3.0	3.0	1	100.00	3.5	even	ok

Figure S21. *tert*-Butyl 3-(2-methoxy-2-oxoethyl)-3-(piperidin-1-yl)azetidine-1-carboxylate (4e) HRMS (ESI-TOF).

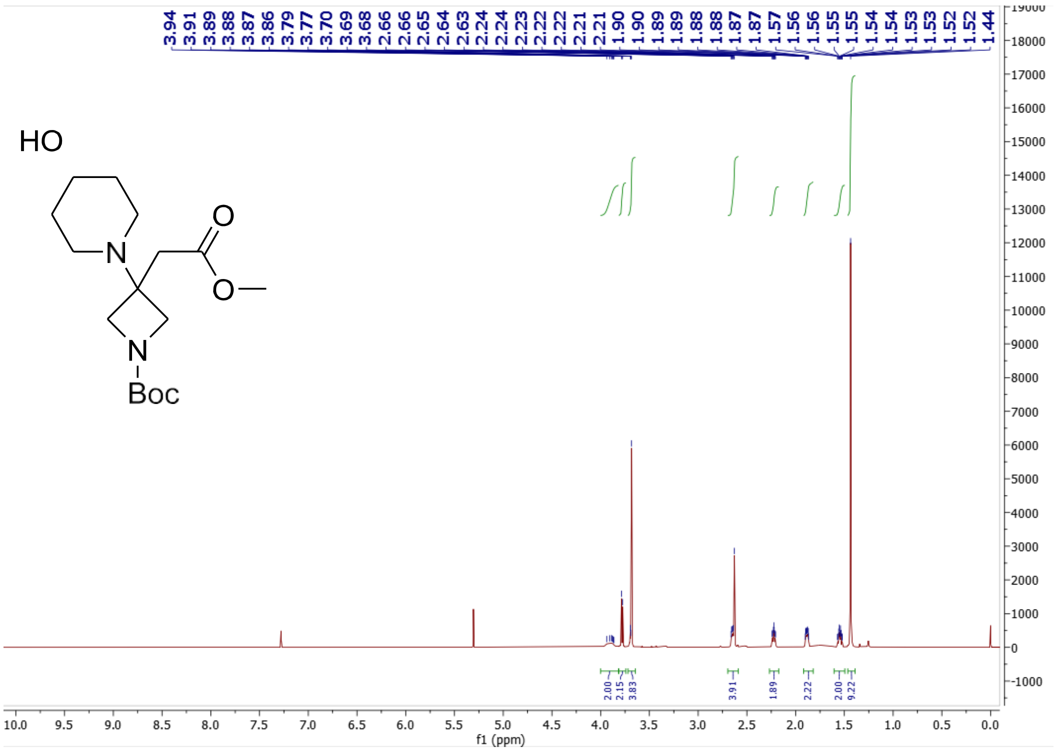

Figure S22. *tert*-Butyl3-(4-hydroxypiperidin-1-yl)-3-(2-methoxy-2-oxoethyl)azetidine-1-carboxylate (4f) ^{1}H NMR spectrum (700 MHz, $CDCl_3$).

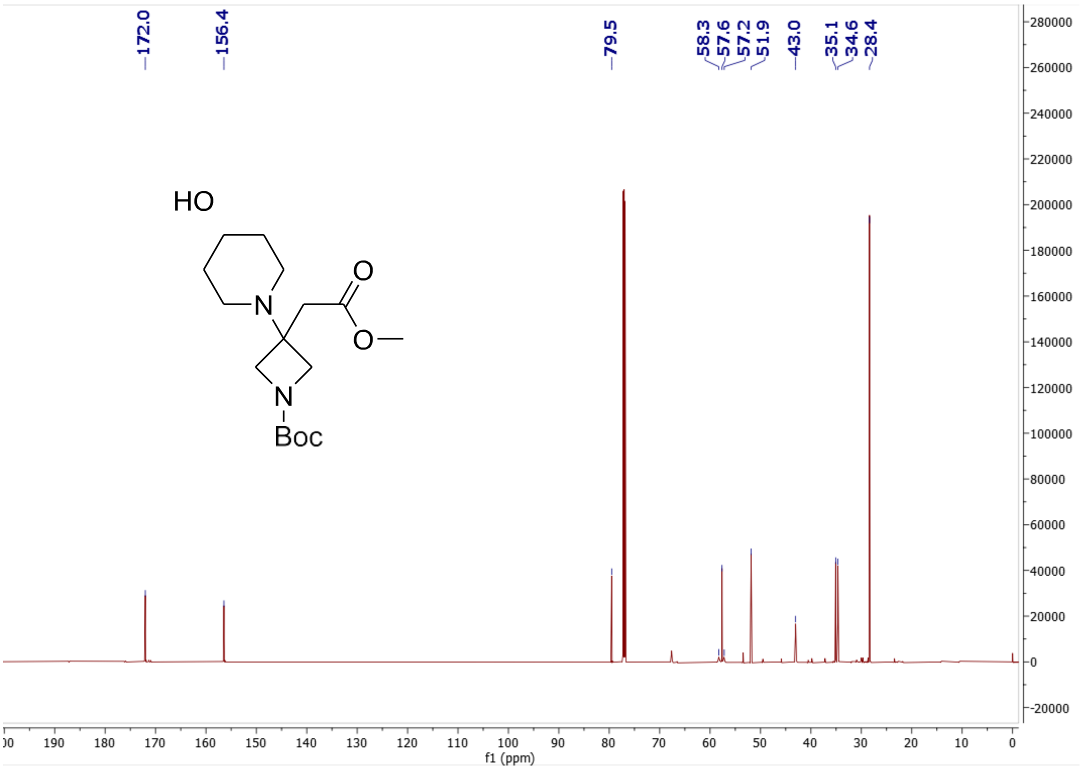

Figure S23. *tert*-Butyl3-(4-hydroxypiperidin-1-yl)-3-(2-methoxy-2-oxoethyl)azetidine-1-carboxylate (4f) ^{13}C NMR spectrum (176 MHz, $CDCl_3$).

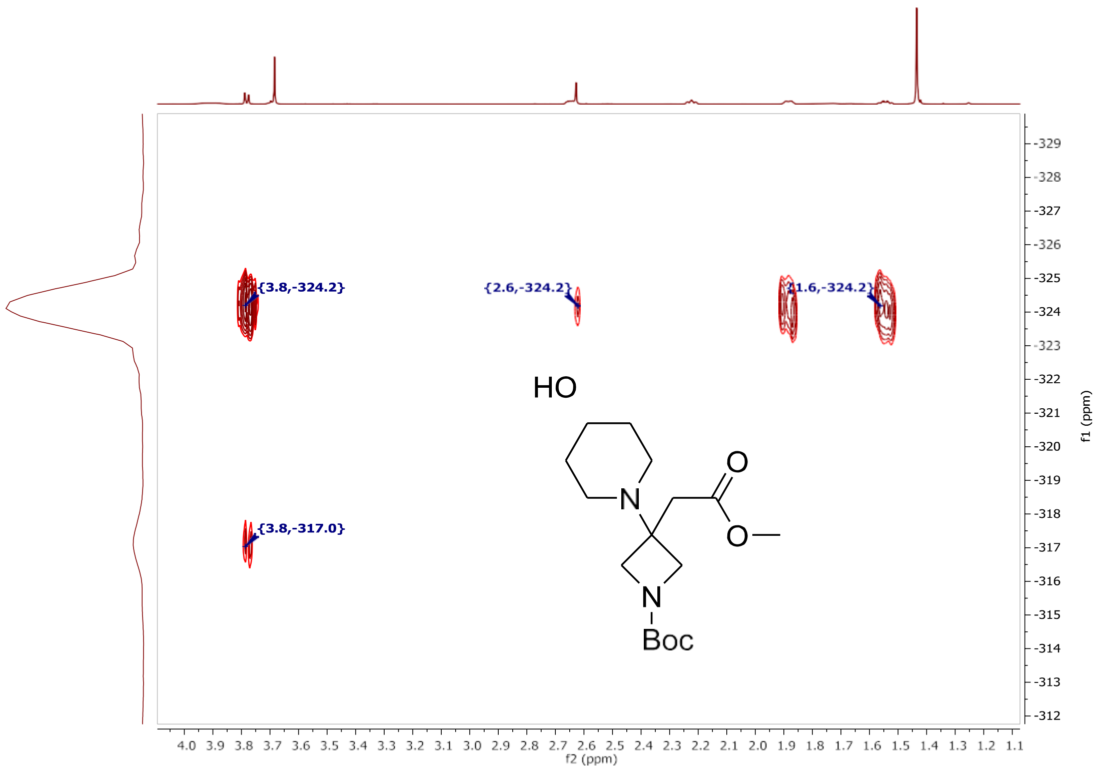

Figure S24. *tert*-Butyl3-(4-hydroxypiperidin-1-yl)-3-(2-methoxy-2-oxoethyl)azetidine-1-carboxylate (4f) ^{1}H-^{15}N HMBC spectrum (71 MHz, $CDCl_3$).

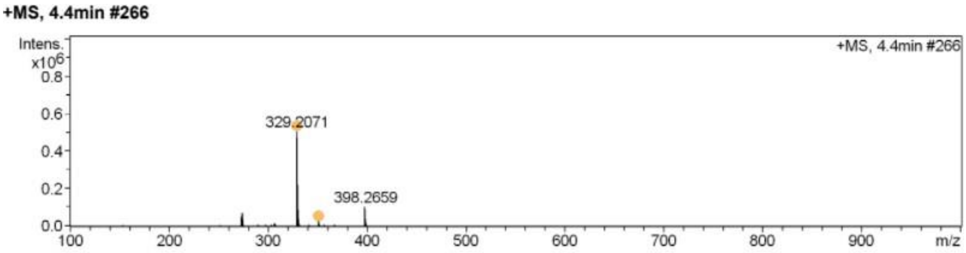

Meas. m/z	#	Ion Formula	m/z	err [ppm]	mSigma	# Sigma	Score	rdb	e⁻ Conf	N-Rule
329.2071	1	C16H29N2O5	329.2071	0.1	1.0	1	100.00	3.5	even	ok
351.1898	1	C16H28N2NaO5	351.1890	-2.1	16.1	1	100.00	3.5	even	ok

Figure S25. *tert*-Butyl3-(4-hydroxypiperidin-1-yl)-3-(2-methoxy-2-oxoethyl)azetidine-1-carboxylate (4f) HRMS (ESI-TOF).

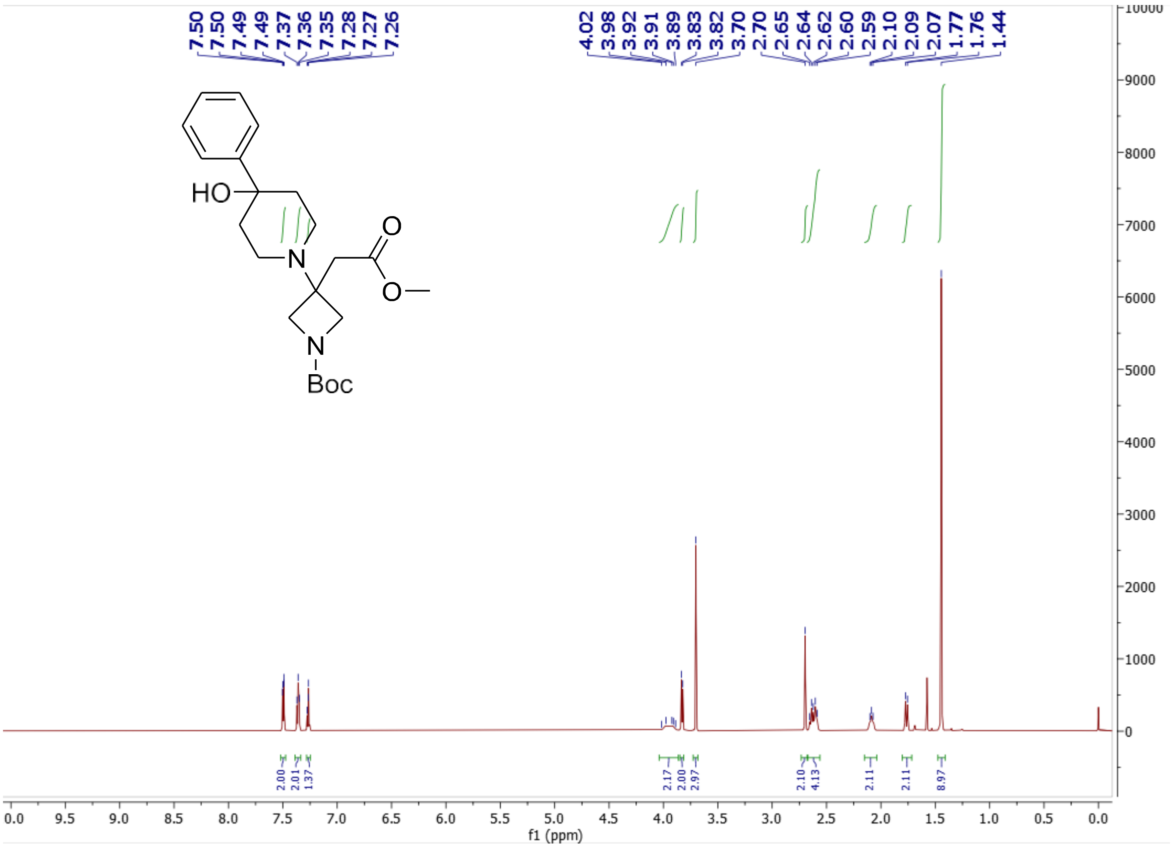

Figure S26. *tert*-Butyl 3-(4-hydroxy-4-phenylpiperidin-1-yl)-3-(2-methoxy-2-oxoethyl)azetidine-1-carboxylate (4g) ^{1}H NMR spectrum (700 MHz, $CDCl_3$).

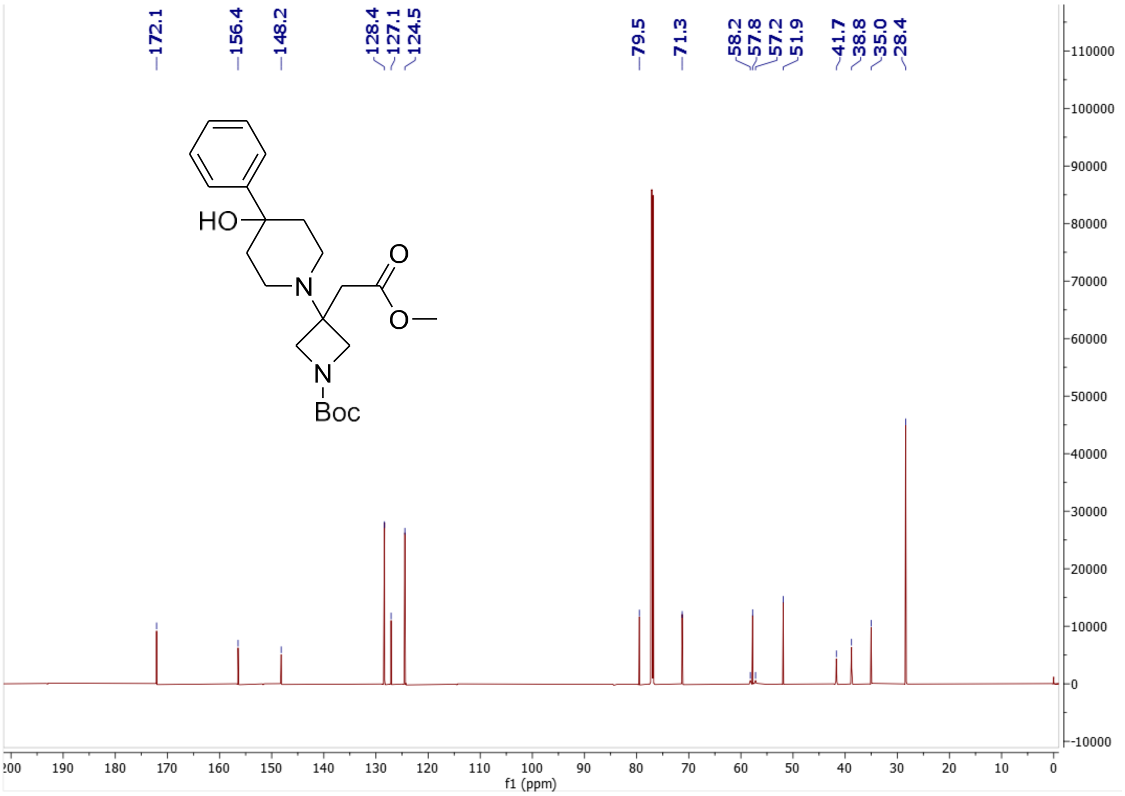

Figure S27. *tert*-Butyl 3-(4-hydroxy-4-phenylpiperidin-1-yl)-3-(2-methoxy-2-oxoethyl)azetidine-1-carboxylate (4g) ^{13}C NMR spectrum (176 MHz, $CDCl_3$).

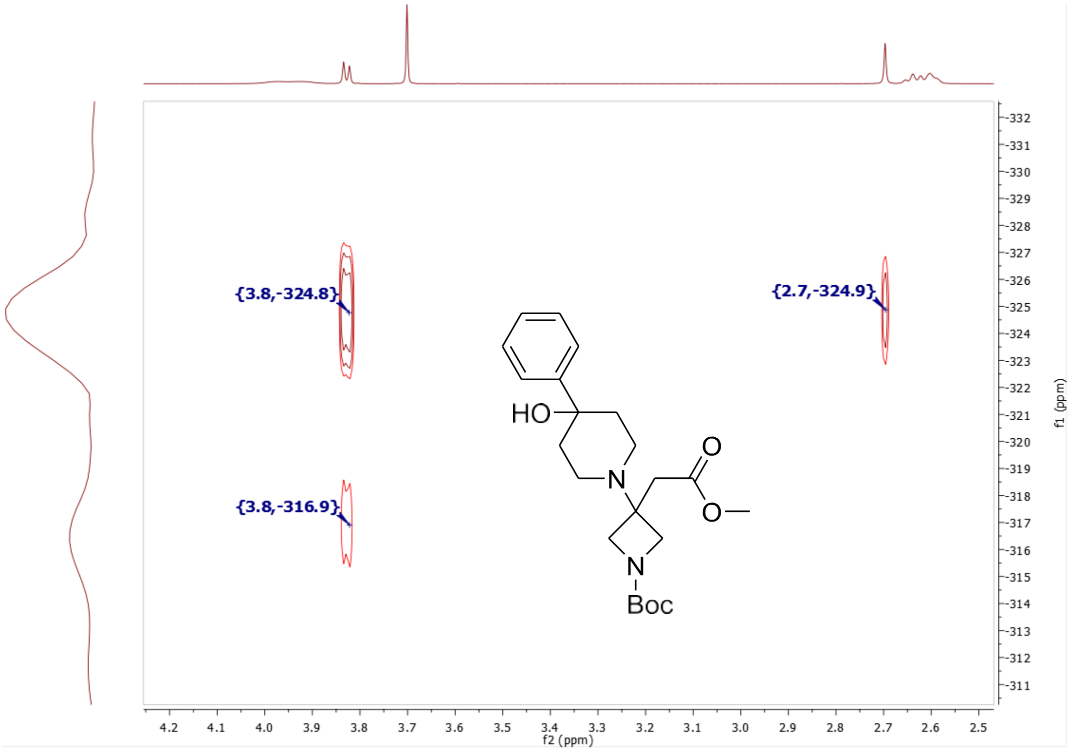

Figure S28. *tert*-Butyl 3-(4-hydroxy-4-phenylpiperidin-1-yl)-3-(2-methoxy-2-oxoethyl)azetidine-1-carboxylate (4g) ^{1}H-^{15}N HMBC spectrum (71 MHz, $CDCl_3$).

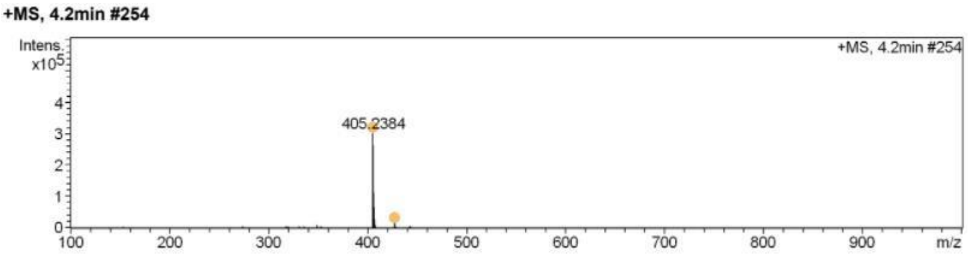

Meas. m/z	#	Ion Formula	m/z	err [ppm]	mSigma	# Sigma	Score	rdb	e⁻ Conf	N-Rule
405.2384	1	C22H33N2O5	405.2384	-0.0	1.9	1	100.00	7.5	even	ok
427.2200	1	C22H32N2NaO5	427.2203	0.8	7.1	1	100.00	7.5	even	ok

Figure S29. *tert*-Butyl 3-(4-hydroxy-4-phenylpiperidin-1-yl)-3-(2-methoxy-2-oxoethyl)azetidine-1-carboxylate (4g) HRMS (ESI-TOF).

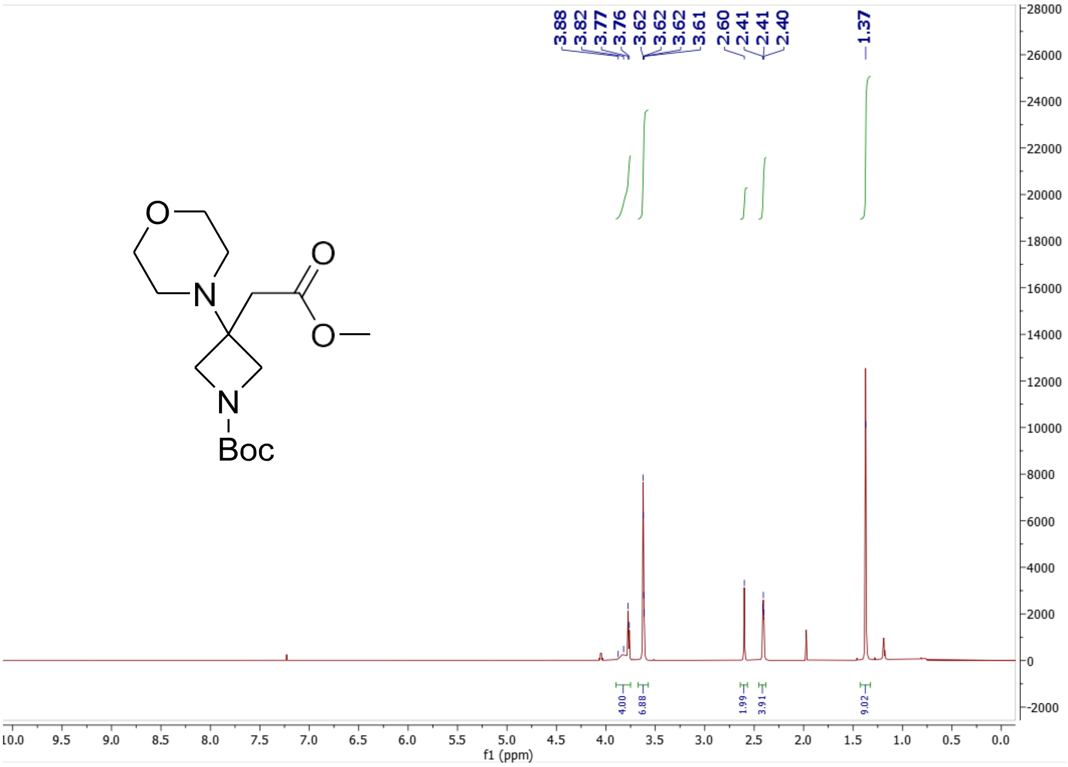

Figure S30. *tert*-Butyl 3-(2-methoxy-2-oxoethyl)-3-(morpholin-4-yl)azetidine-1-carboxylate (4h) ^{1}H NMR spectrum (700 MHz, $CDCl_3$).

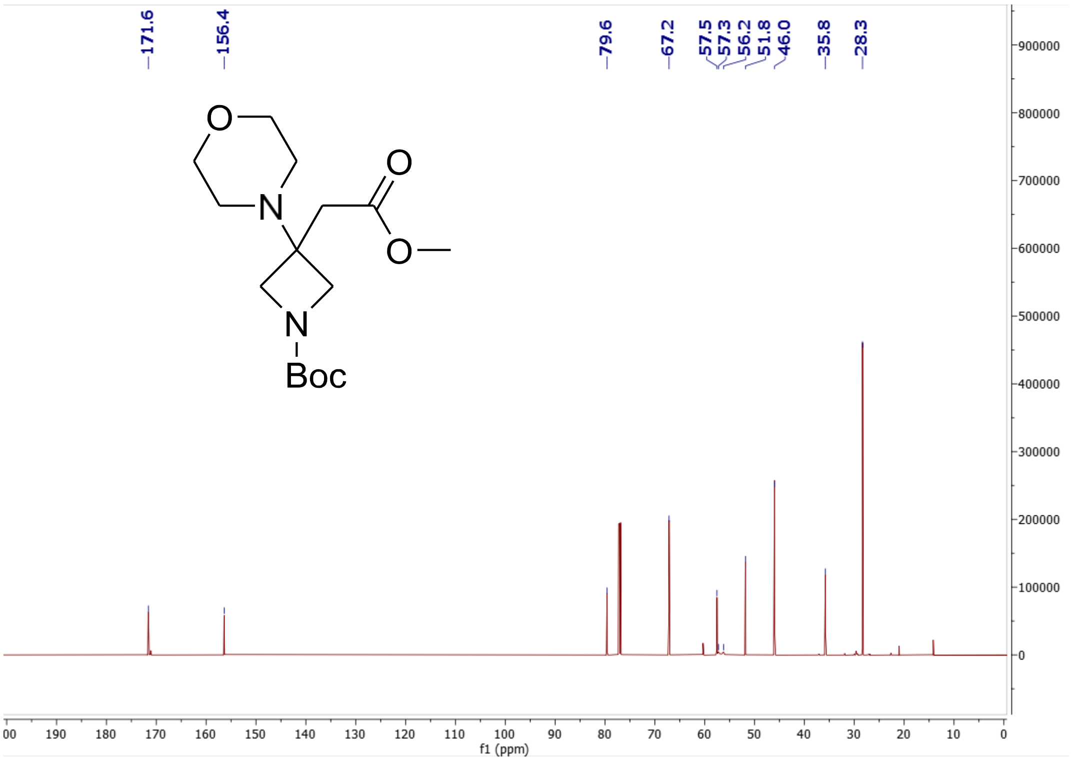

Figure S31. *tert*-Butyl 3-(2-methoxy-2-oxoethyl)-3-(morpholin-4-yl)azetidine-1-carboxylate (4h) ^{13}C NMR spectrum (176 MHz, $CDCl_3$).

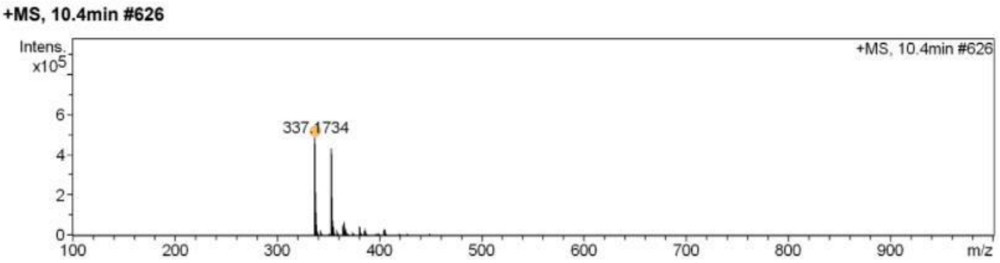

Meas. m/z	#	Ion Formula	m/z	err [ppm]	mSigma	# Sigma	Score	rdb	e⁻ Conf	N-Rule
337.1734	1	C15H26N2NaO5	337.1734	0.0	48.5	1	100.00	3.5	even	ok

Figure S32. *tert*-Butyl 3-(2-methoxy-2-oxoethyl)-3-(morpholin-4-yl)azetidine-1-carboxylate (4h) HRMS (ESI-TOF).

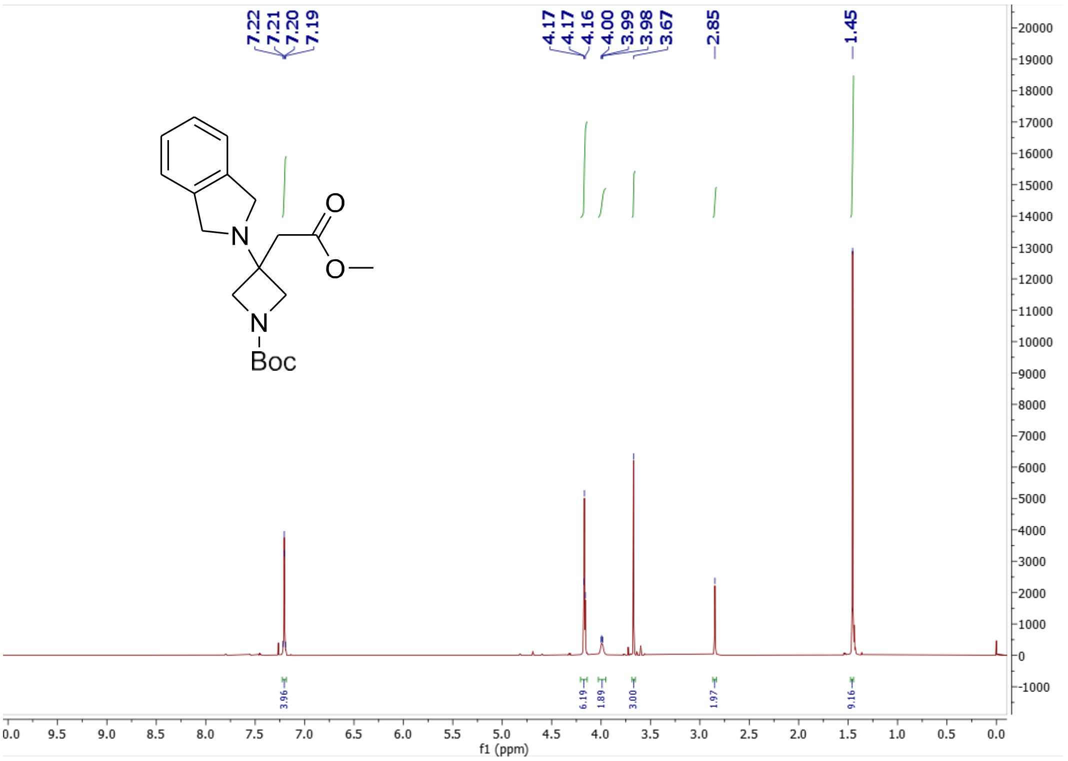

Figure S33. *tert*-Butyl 3-(1,3-dihydro-2*H*-isoindol-2-yl)-3-(2-methoxy-2-oxoethyl)azetidine-1-carboxylate (4i) ^{1}H NMR spectrum (700 MHz, $CDCl_3$).

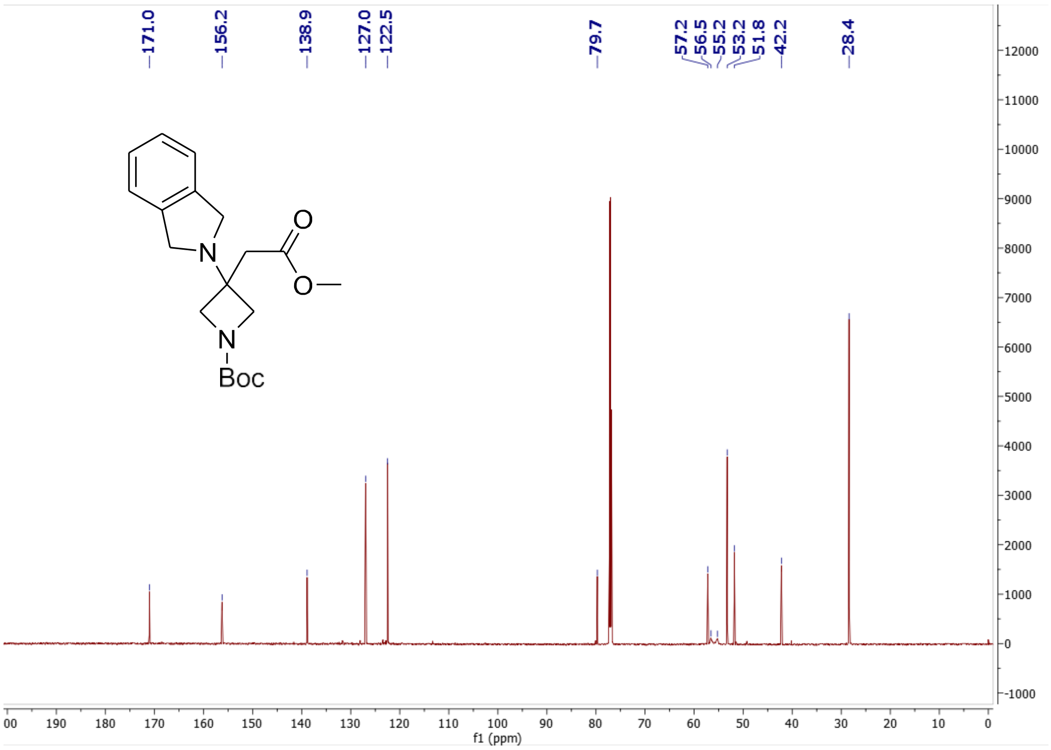

Figure S34. *tert*-Butyl 3-(1,3-dihydro-2*H*-isoindol-2-yl)-3-(2-methoxy-2-oxoethyl)azetidine-1- carboxylate (4i) ^{13}C NMR spectrum (176 MHz, $CDCl_3$).

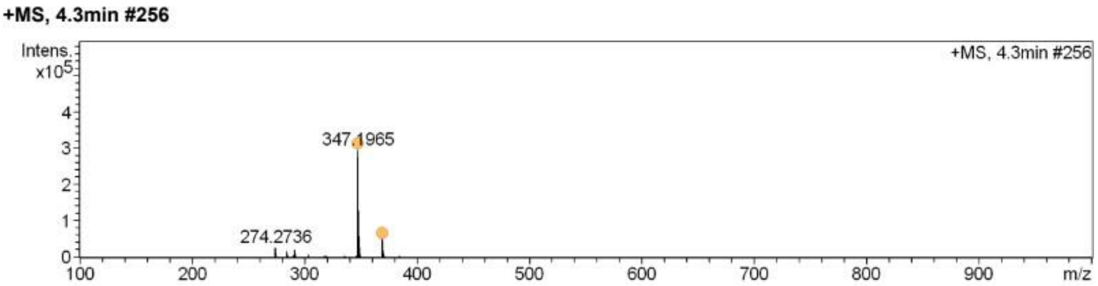

Meas. m/z	#	Ion Formula	m/z	err [ppm]	mSigma	# Sigma	Score	rdb	e⁻ Conf	N-Rule
347.1965	1	C19H27N2O4	347.1965	-0.1	4.8	1	100.00	7.5	even	ok
369.1777	1	C19H26N2NaO4	369.1785	2.0	7.2	1	100.00	7.5	even	ok

Figure S35. *tert*-Butyl 3-(1,3-dihydro-2*H*-isoindol-2-yl)-3-(2-methoxy-2-oxoethyl)azetidine-1- carboxylate (4i) HRMS (ESI-TOF).

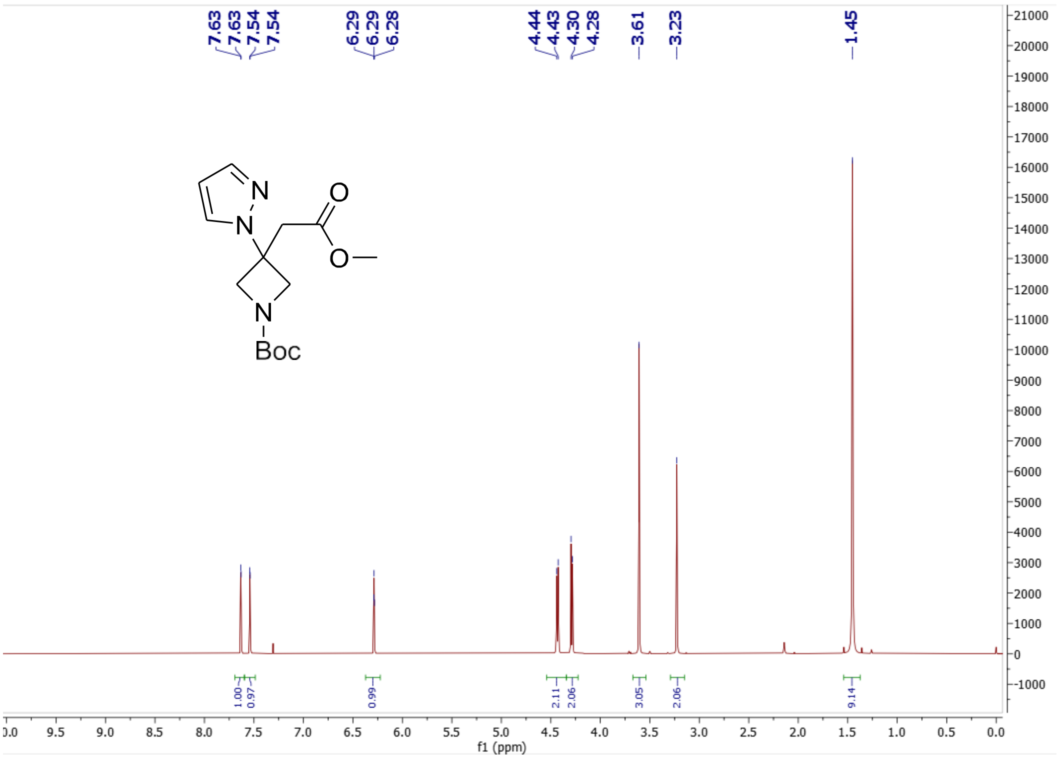

Figure S36. *tert*-Butyl 3-(2-methoxy-2-oxoethyl)-3-(1*H*-pyrazol-1-yl)azetidine-1-carboxylate (4j) ^{1}H NMR spectrum (700 MHz, $CDCl_3$).

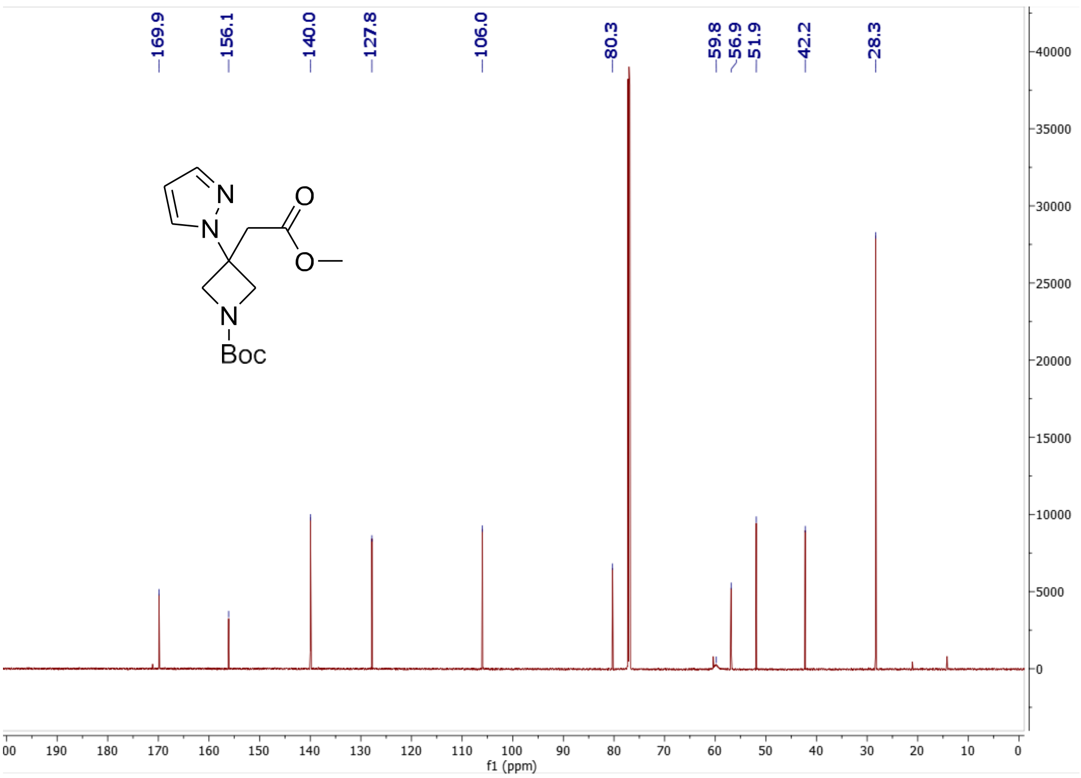

Figure S37. *tert*-Butyl 3-(2-methoxy-2-oxoethyl)-3-(1*H*-pyrazol-1-yl)azetidine-1-carboxylate (4j) ^{13}C NMR spectrum (176 MHz, $CDCl_3$).

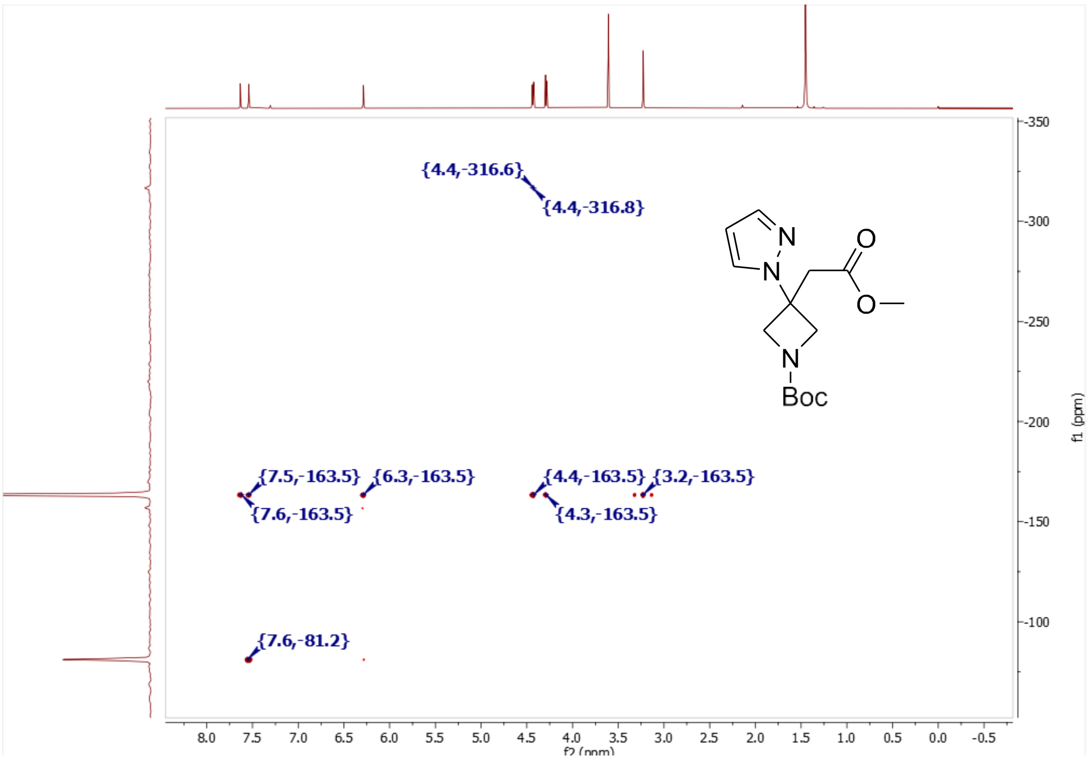

Figure S38. *tert*-Butyl 3-(2-methoxy-2-oxoethyl)-3-(1*H*-pyrazol-1-yl)azetidine-1-carboxylate (4j) ^{1}H-^{15}N HMBC spectrum (71 MHz, $CDCl_3$).

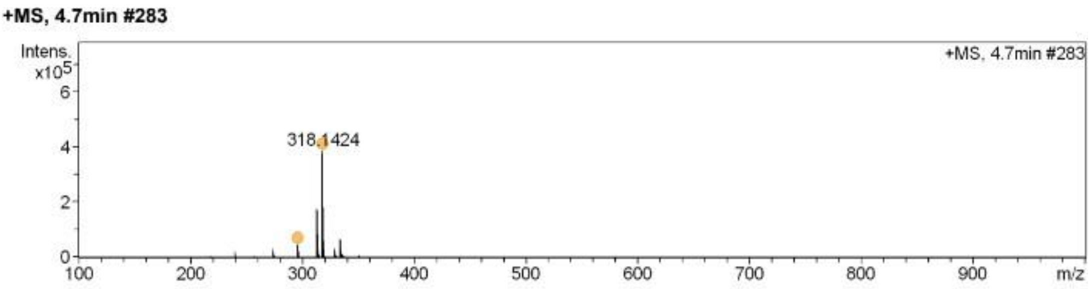

Meas. m/z	#	Ion Formula	m/z	err [ppm]	mSigma	# Sigma	Score	rdb	e⁻ Conf	N-Rule
296.1591	1	C14H22N3O4	296.1605	4.7	1.2	1	100.00	5.5	even	ok
318.1424	1	C14H21N3NaO4	318.1424	-0.2	2.6	1	100.00	5.5	even	ok

Figure S39. *tert*-Butyl 3-(2-methoxy-2-oxoethyl)-3-(1*H*-pyrazol-1-yl)azetidine-1-carboxylate (4j) HRMS (ESI-TOF).

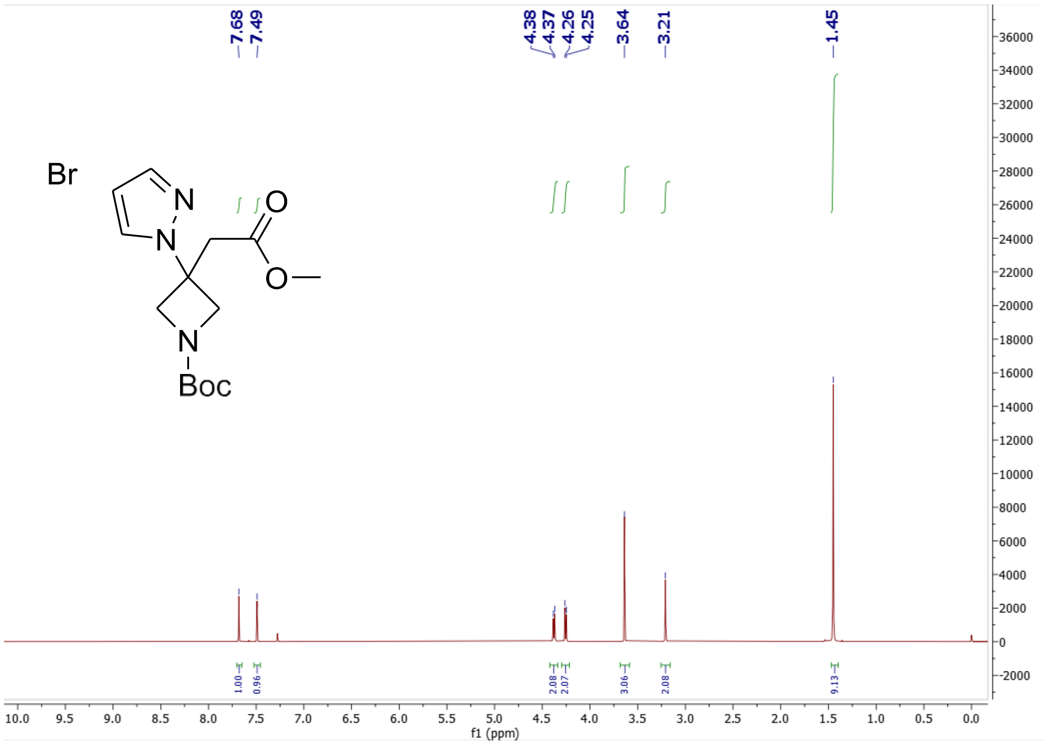

Figure S40. *tert*-Butyl 3-(4-bromo-1*H*-pyrazol-1-yl)-3-(2-methoxy-2-oxoethyl)azetidine-1-carboxylate (4k) ^{1}H NMR spectrum (700 MHz, $CDCl_3$).

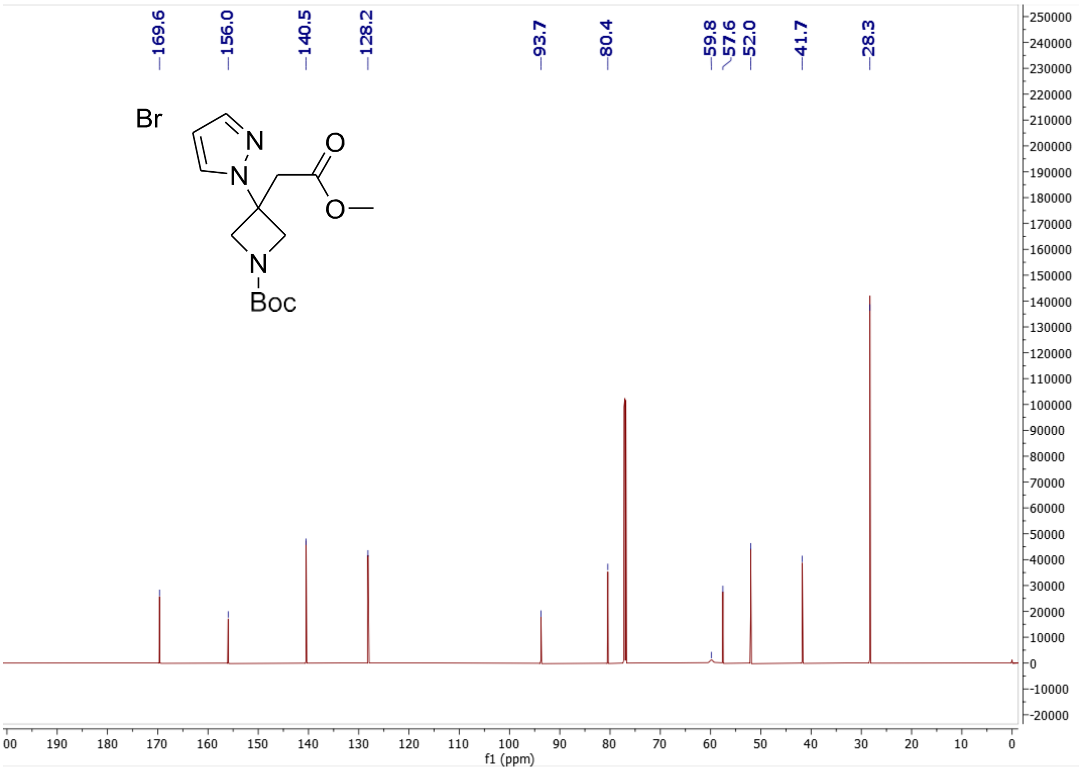

Figure S41. *tert*-Butyl 3-(4-bromo-1*H*-pyrazol-1-yl)-3-(2-methoxy-2-oxoethyl)azetidine-1-carboxylate (4k) ^{13}C NMR spectrum (176 MHz, $CDCl_3$).

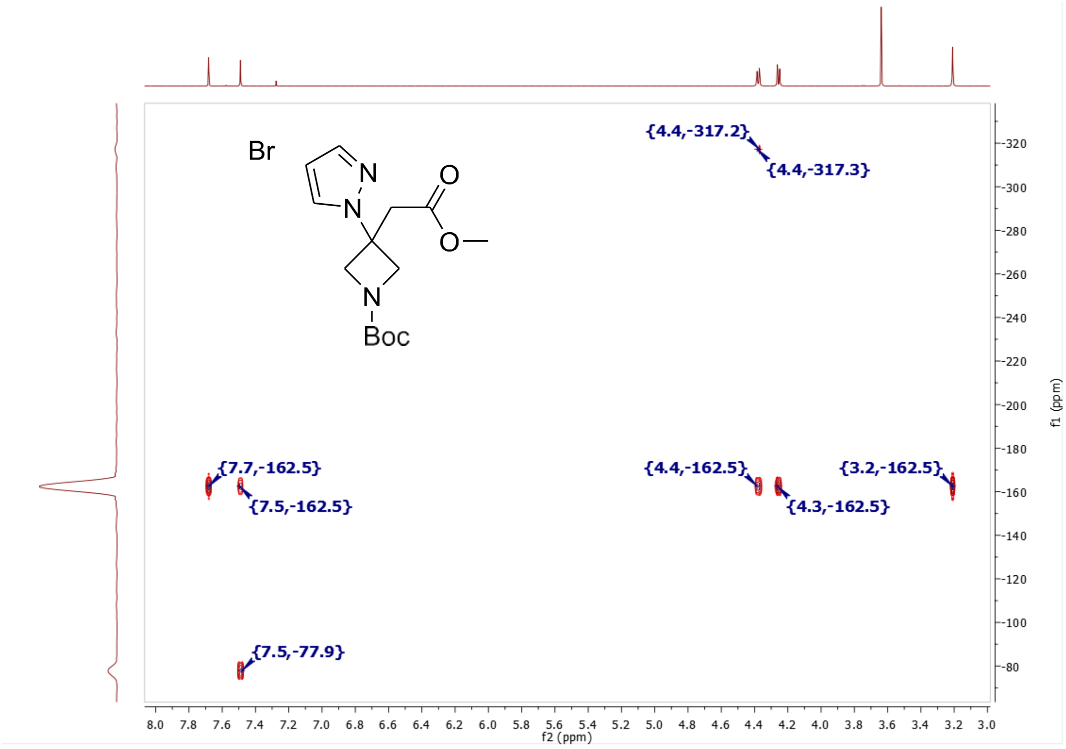

Figure S42. *tert*-Butyl 3-(4-bromo-1*H*-pyrazol-1-yl)-3-(2-methoxy-2-oxoethyl)azetidine-1-carboxylate (4k) ^{1}H-^{15}N HMBC spectrum (71 MHz, $CDCl_3$).

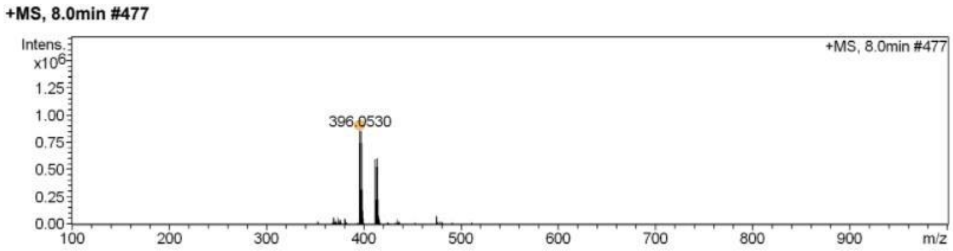

Meas. m/z	#	Ion Formula	m/z	err [ppm]	mSigma	# Sigma	Score	rdb	e⁻ Conf	N-Rule
396.0530	1	C14H20BrN3NaO4	396.0529	0.1	10.9	1	100.00	5.5	even	ok

Figure S43. *tert*-Butyl 3-(4-bromo-1*H*-pyrazol-1-yl)-3-(2-methoxy-2-oxoethyl)azetidine-1-carboxylate (4k) HRMS (ESI-TOF).

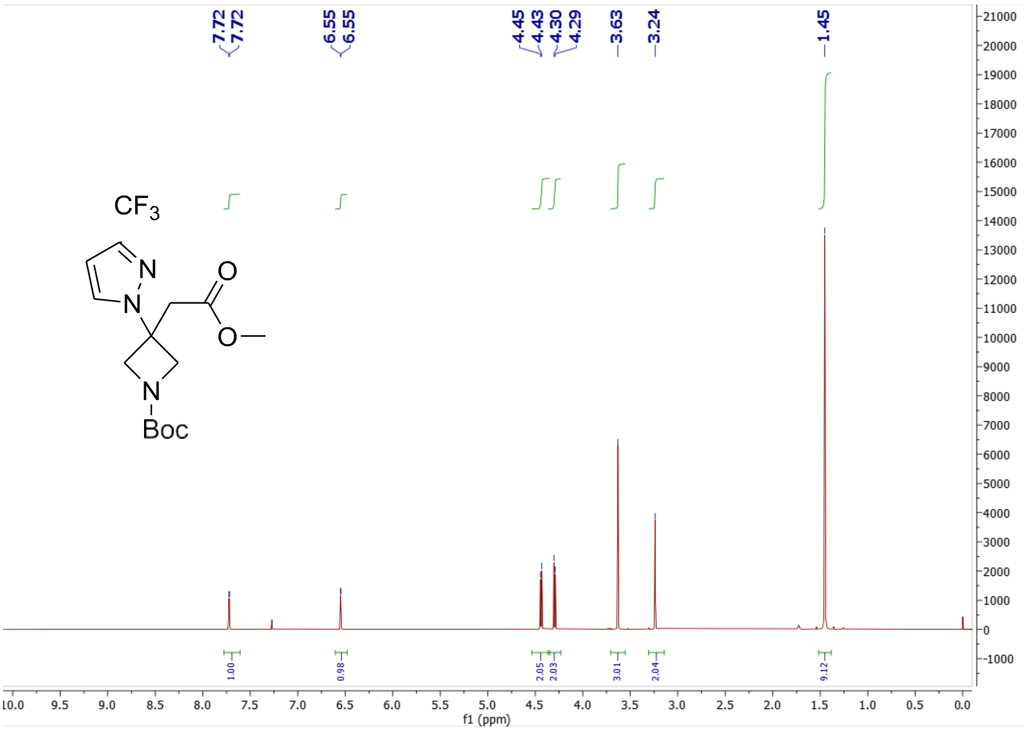

Figure S44. *tert*-Butyl 3-(2-methoxy-2-oxoethyl)-3-[3-(trifluoromethyl)-1*H*-pyrazol-1-yl]azetidine-1- carboxylate (4l) ^{1}H NMR spectrum (700 MHz, $CDCl_3$).

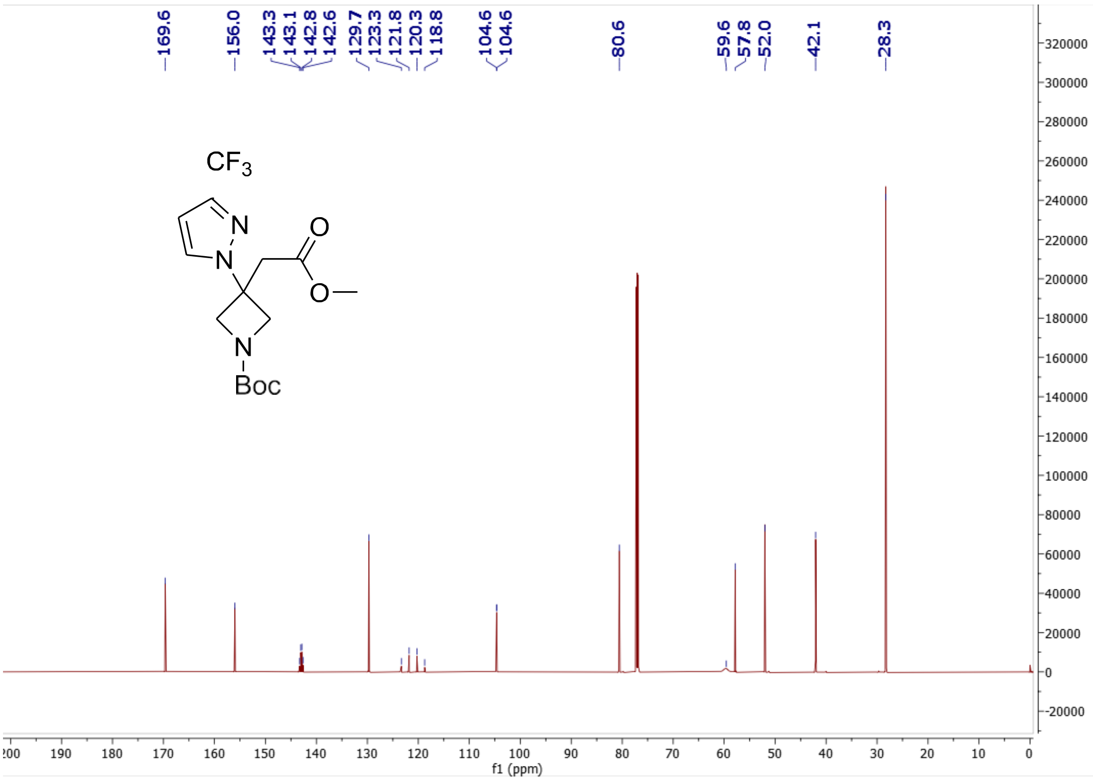

Figure S45. *tert*-Butyl 3-(2-methoxy-2-oxoethyl)-3-[3-(trifluoromethyl)-1*H*-pyrazol-1-yl]azetidine-1- carboxylate (4l) ^{13}C NMR spectrum (176 MHz, $CDCl_3$).

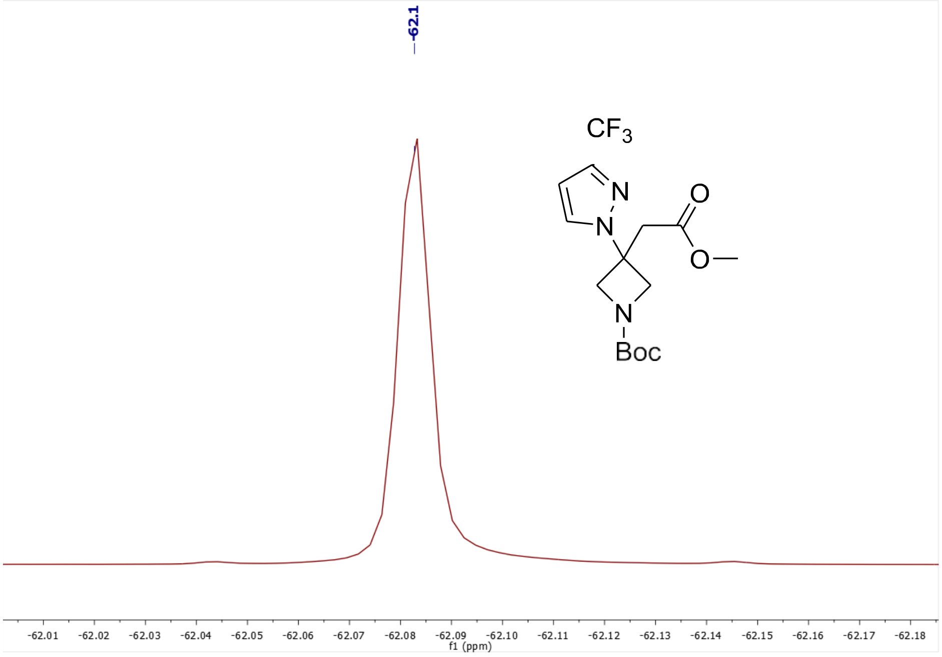

Figure S46. *tert*-Butyl 3-(2-methoxy-2-oxoethyl)-3-[3-(trifluoromethyl)-1*H*-pyrazol-1-yl]azetidine-1- carboxylate (4l) ^{19}F NMR spectrum (376 MHz, $CDCl_3$).

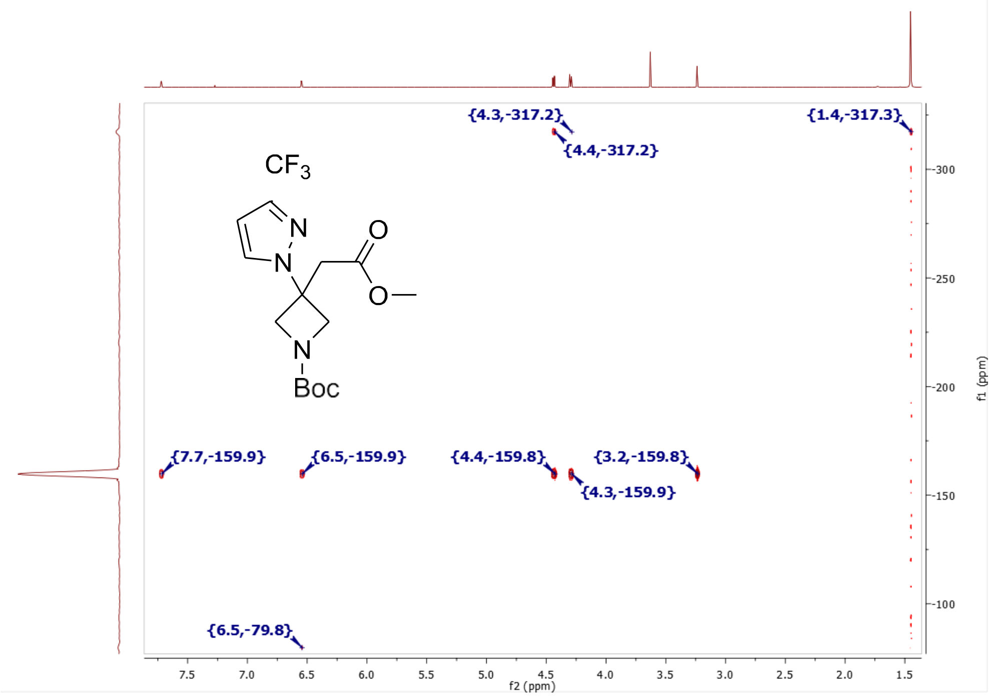

Figure S47. *tert*-Butyl 3-(2-methoxy-2-oxoethyl)-3-[3-(trifluoromethyl)-1*H*-pyrazol-1-yl]azetidine-1- carboxylate (4l) ^{1}H-^{15}N HMBC NMR spectrum (71 MHz, $CDCl_3$).

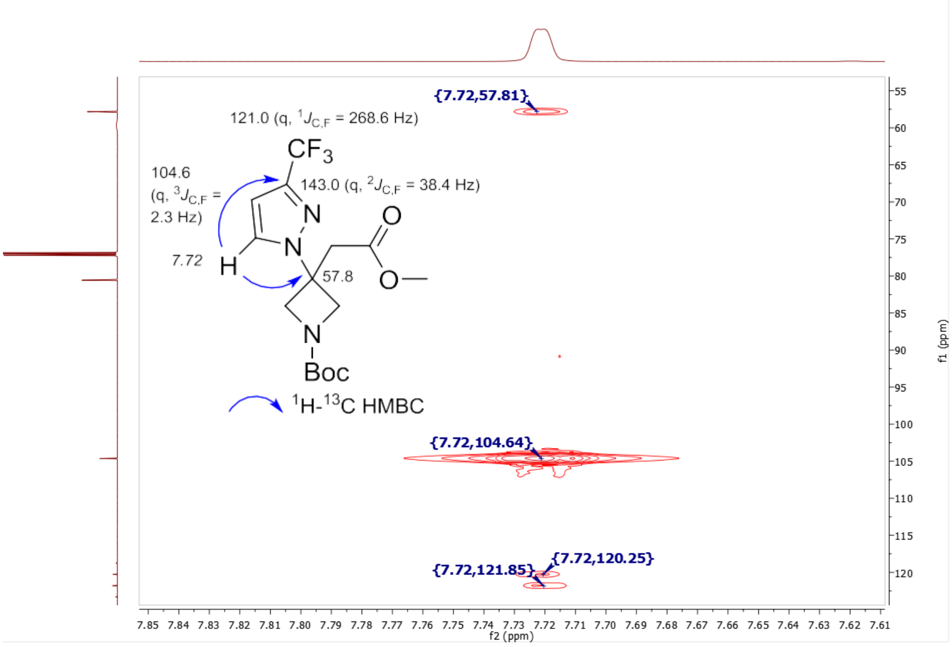

Figure S48. *tert*-Butyl 3-(2-methoxy-2-oxoethyl)-3-[3-(trifluoromethyl)-1*H*-pyrazol-1-yl]azetidine-1- carboxylate (4l) ^{1}H-^{13}C HMBC NMR spectrum (176 MHz, $CDCl_3$).

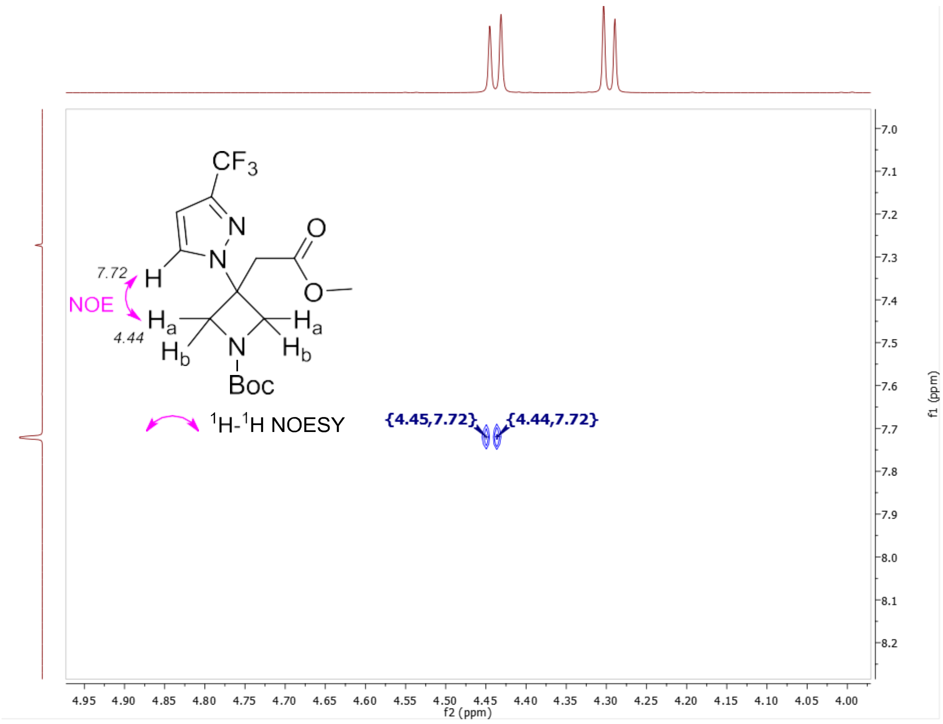

Figure S49. *tert*-Butyl 3-(2-methoxy-2-oxoethyl)-3-[3-(trifluoromethyl)-1*H*-pyrazol-1-yl]azetidine-1- carboxylate (4l) ^{1}H-^{1}H NOESY NMR spectrum (700 MHz, $CDCl_3$).

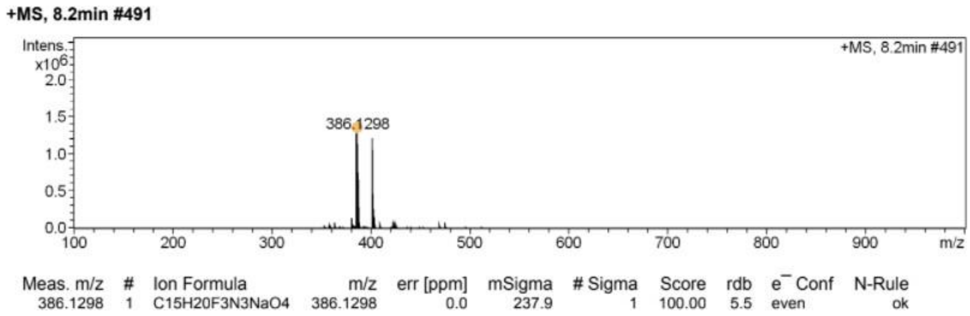

Figure S50. *tert*-Butyl 3-(2-methoxy-2-oxoethyl)-3-[3-(trifluoromethyl)-1*H*-pyrazol-1-yl]azetidine-1- carboxylate (4l) HRMS (ESI-TOF).

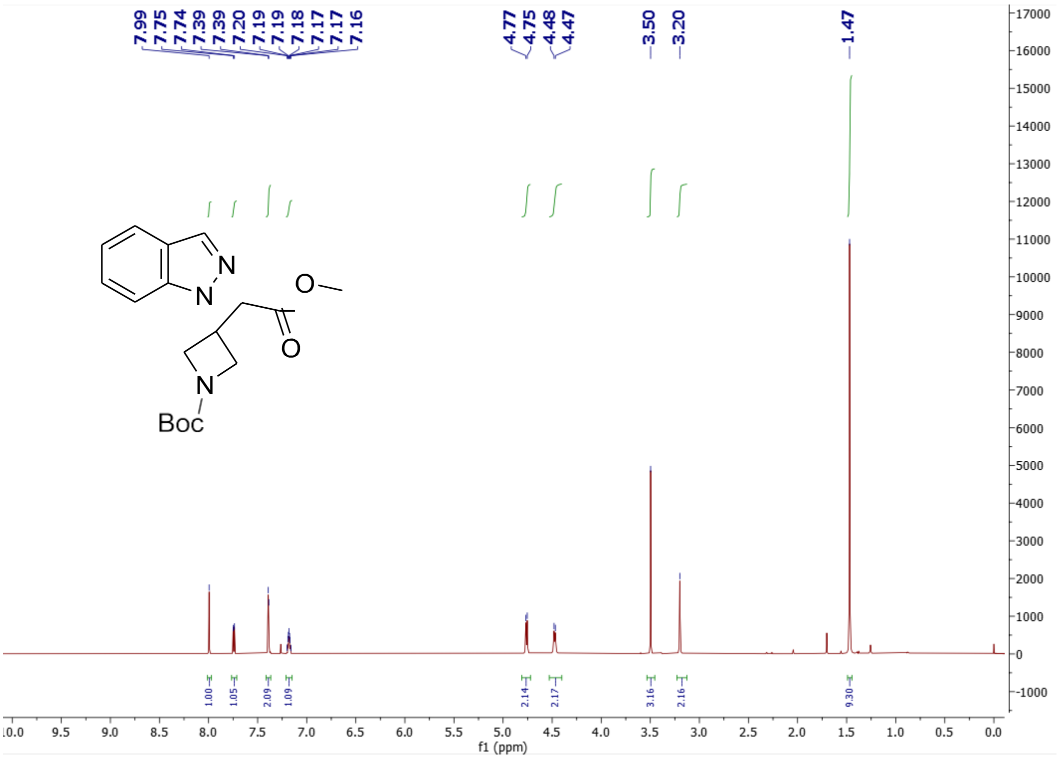

Figure S51. *tert*-butyl 3-(1*H*-indazol-1-yl)-3-(2-methoxy-2-oxoethyl)azetidine-1-carboxylate (4m) ^{1}H NMR spectrum (700 MHz, $CDCl_3$).

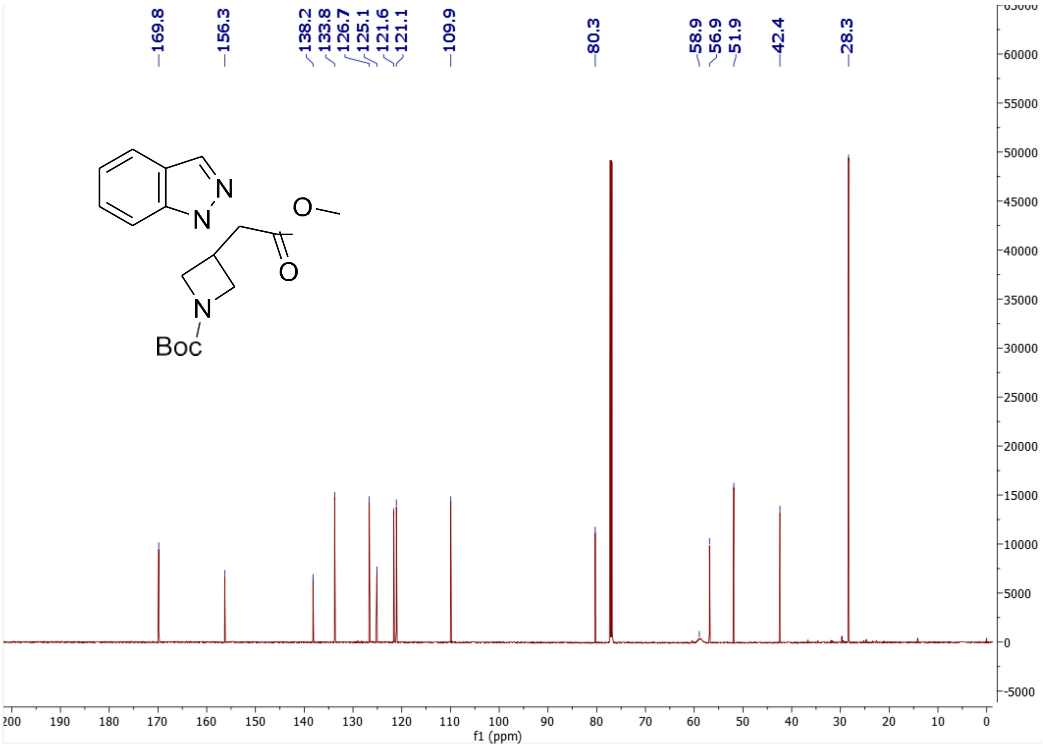

Figure S52. *tert*-butyl 3-(1*H*-indazol-1-yl)-3-(2-methoxy-2-oxoethyl)azetidine-1-carboxylate (4m) ^{13}C NMR spectrum (176 MHz, $CDCl_3$).

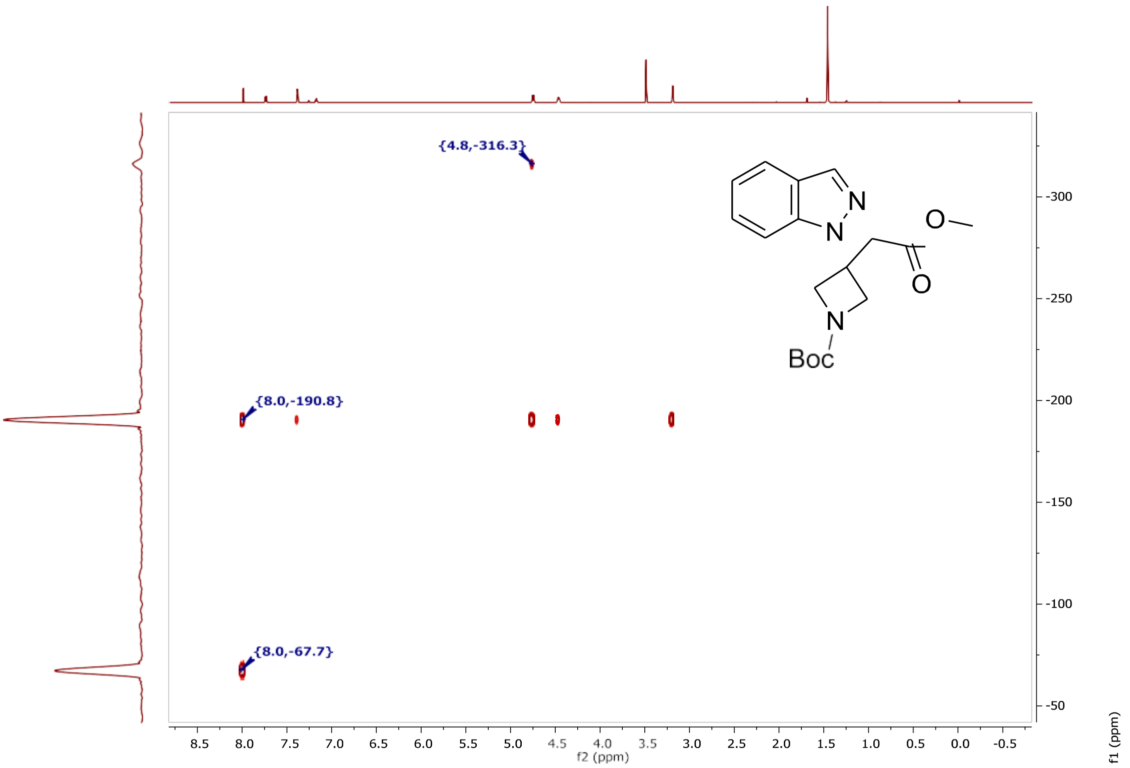

Figure S53. *tert*-butyl 3-(1*H*-indazol-1-yl)-3-(2-methoxy-2-oxoethyl)azetidine-1-carboxylate (4m) ^{1}H-^{15}N HMBC spectrum (71 MHz, $CDCl_3$).

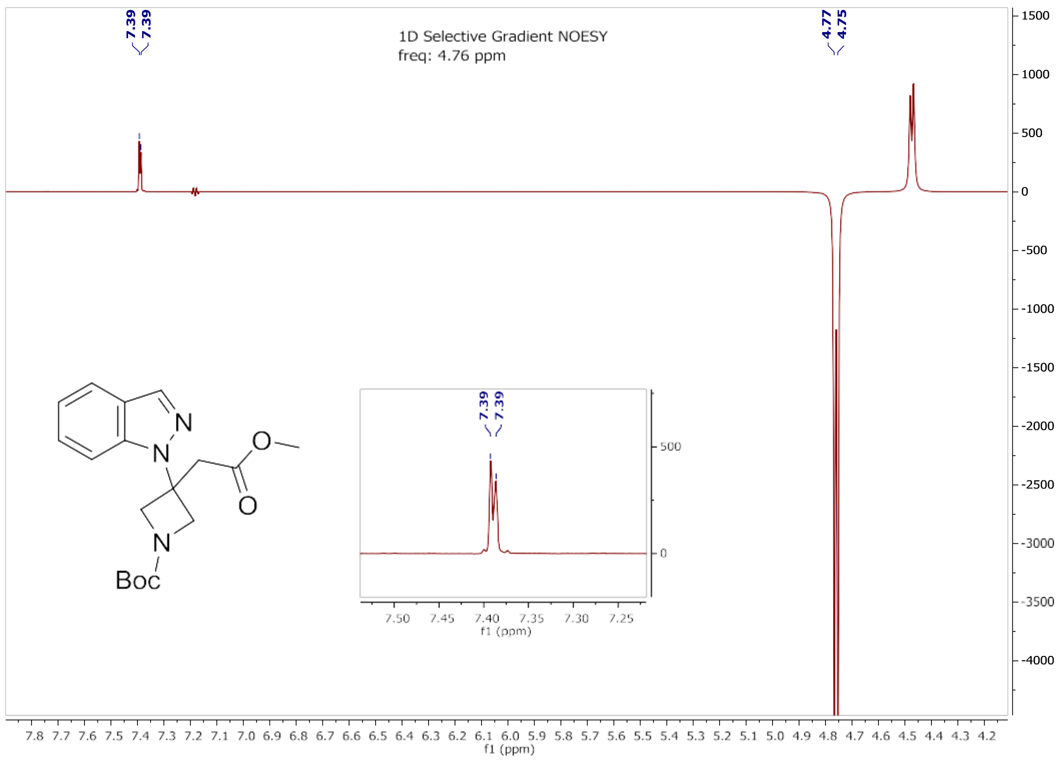

Figure S54. *tert*-butyl 3-(1*H*-indazol-1-yl)-3-(2-methoxy-2-oxoethyl)azetidine-1-carboxylate (4m) 1D Selective Gradient NOESY NMR spectrum (700 MHz, $CDCl_3$).

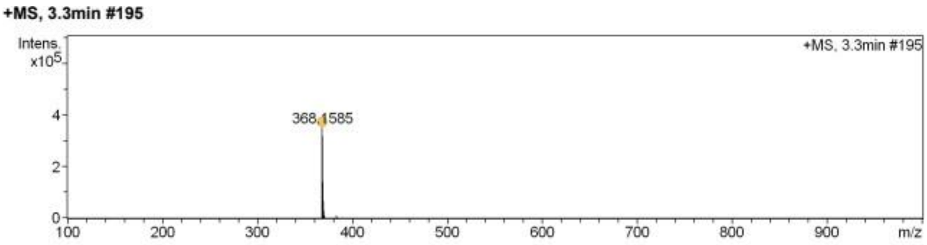

Meas. m/z	#	Ion Formula	m/z	err [ppm]	mSigma	# Sigma	Score	rdb	e⁻ Conf	N-Rule
368.1585	1	C18H23N3NaO4	368.1581	-1.3	1.9	1	100.00	8.5	even	ok

Figure S55. *tert*-butyl 3-(1*H*-indazol-1-yl)-3-(2-methoxy-2-oxoethyl)azetidine-1-carboxylate (4m) HRMS (ESI-TOF).

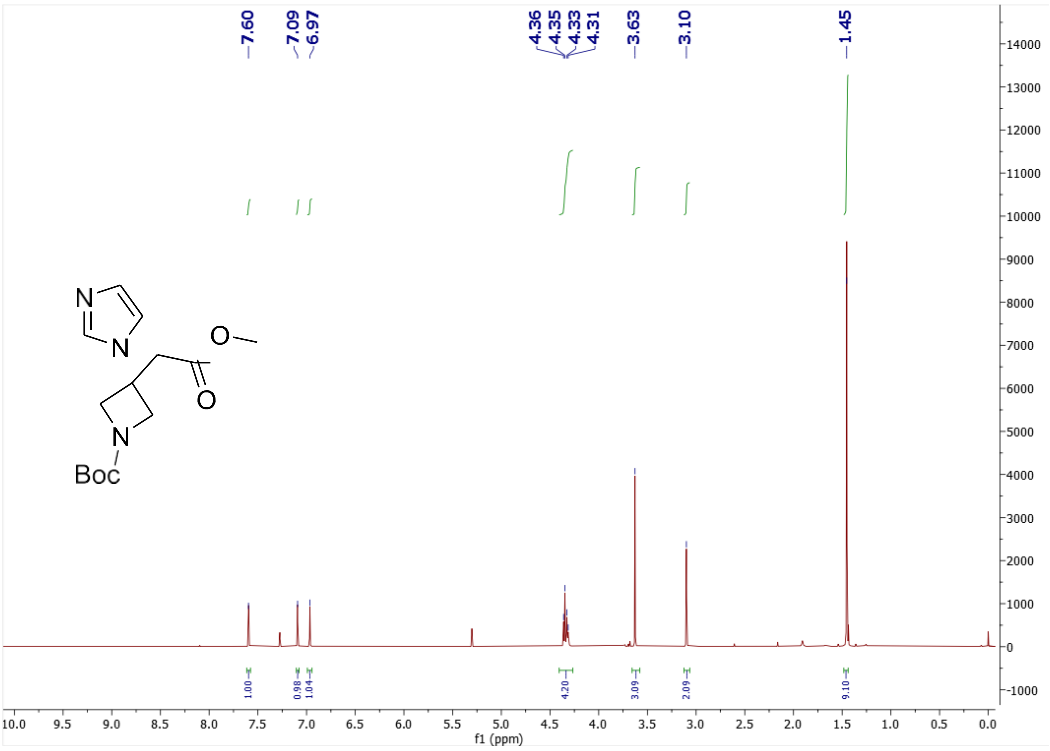

Figure S56. *tert*-Butyl 3-(1*H*-imidazol-1-yl)-3-(2-methoxy-2-oxoethyl)azetidine-1-carboxylate (4n) ^{1}H NMR spectrum (700 MHz, $CDCl_3$).

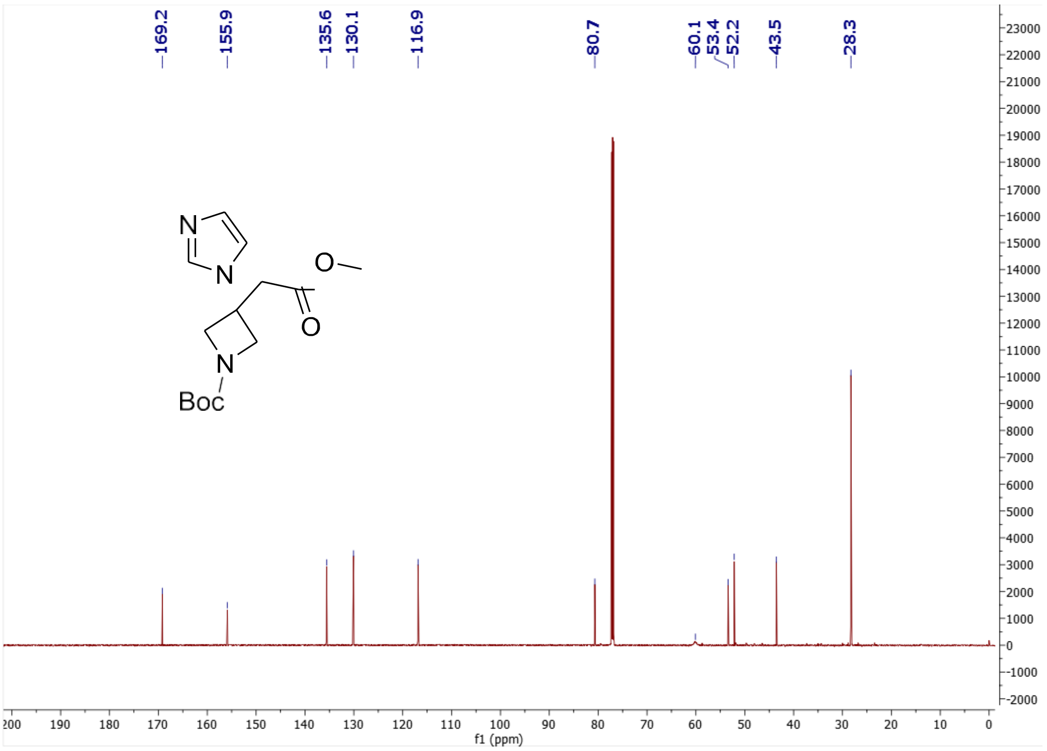

Figure S57. *tert*-Butyl 3-(1*H*-imidazol-1-yl)-3-(2-methoxy-2-oxoethyl)azetidine-1-carboxylate (4n) ^{13}C NMR spectrum (176 MHz, $CDCl_3$).

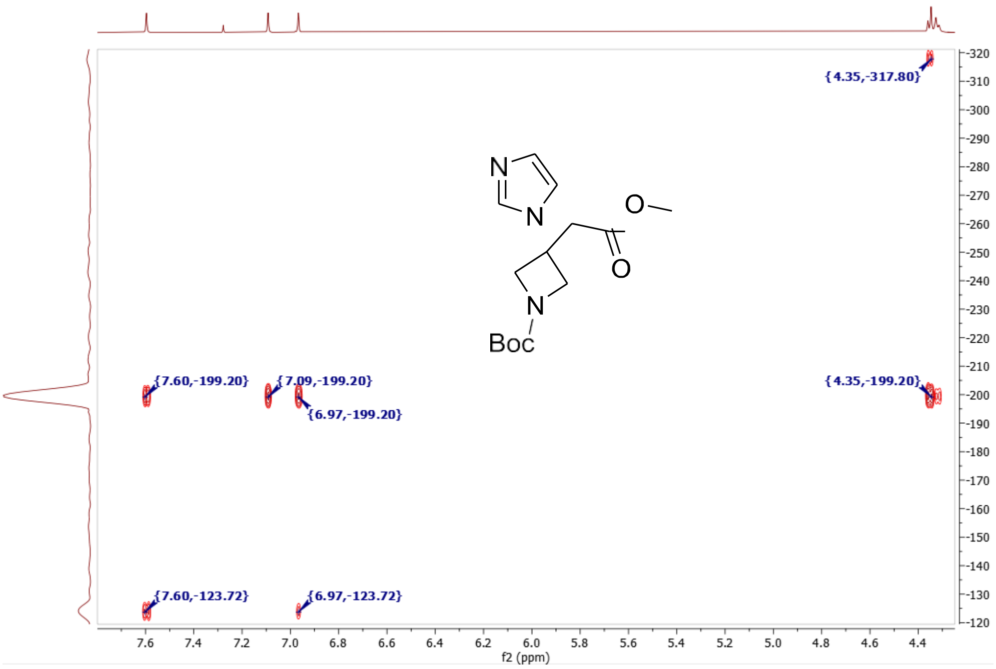

Figure S58. *tert*-Butyl 3-(1*H*-imidazol-1-yl)-3-(2-methoxy-2-oxoethyl)azetidine-1-carboxylate (4n) ^{1}H- ^{15}N HMBC spectrum (71 MHz, $CDCl_3$).

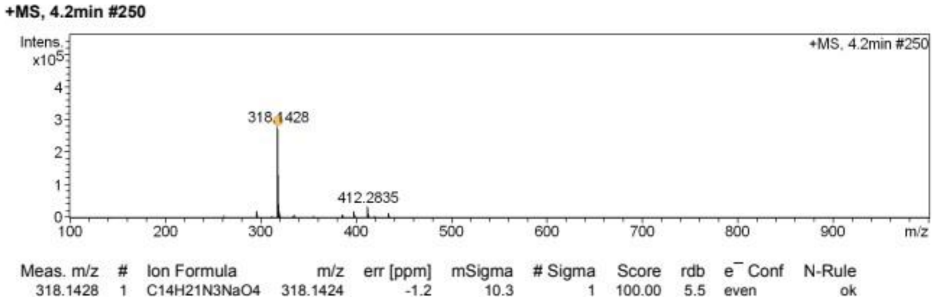

Figure S59. *tert*-Butyl 3-(1*H*-imidazol-1-yl)-3-(2-methoxy-2-oxoethyl)azetidine-1-carboxylate (4n) HRMS (ESI-TOF).

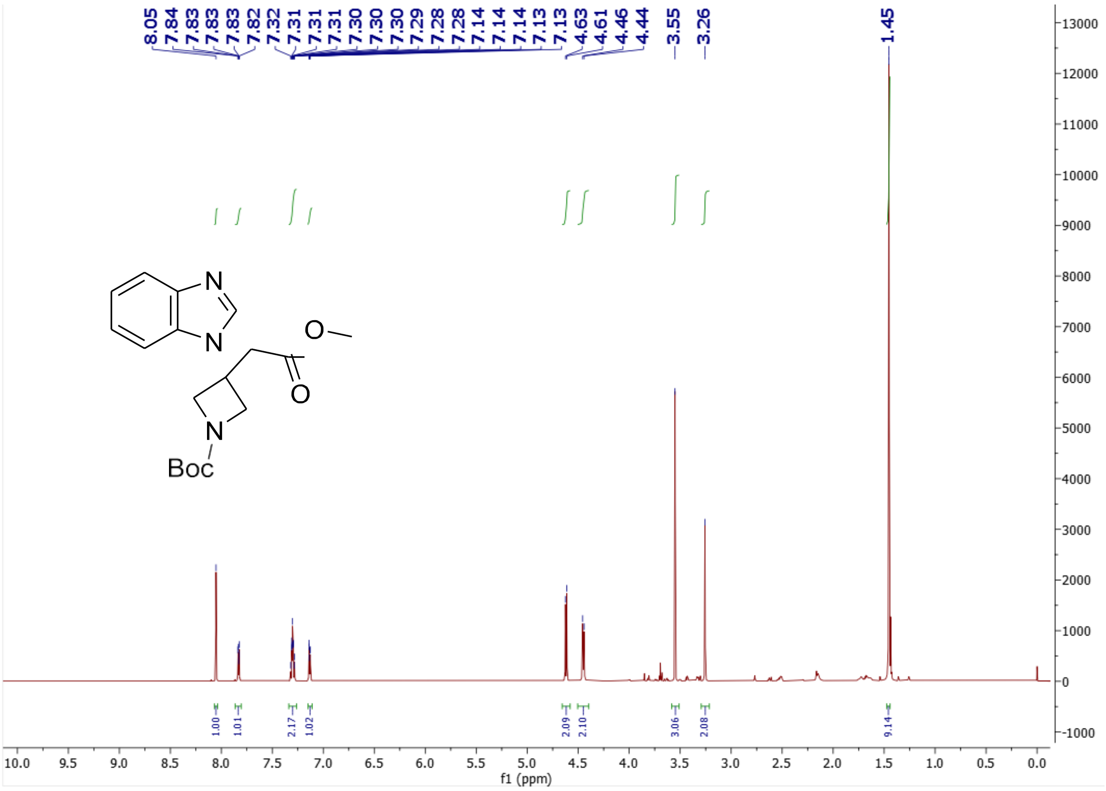

Figure S60. *tert*-Butyl 3-(1*H*-benzimidazol-1-yl)-3-(2-methoxy-2-oxoethyl)azetidine-1-carboxylate (4o) ^{1}H NMR spectrum (700 MHz, $CDCl_3$).

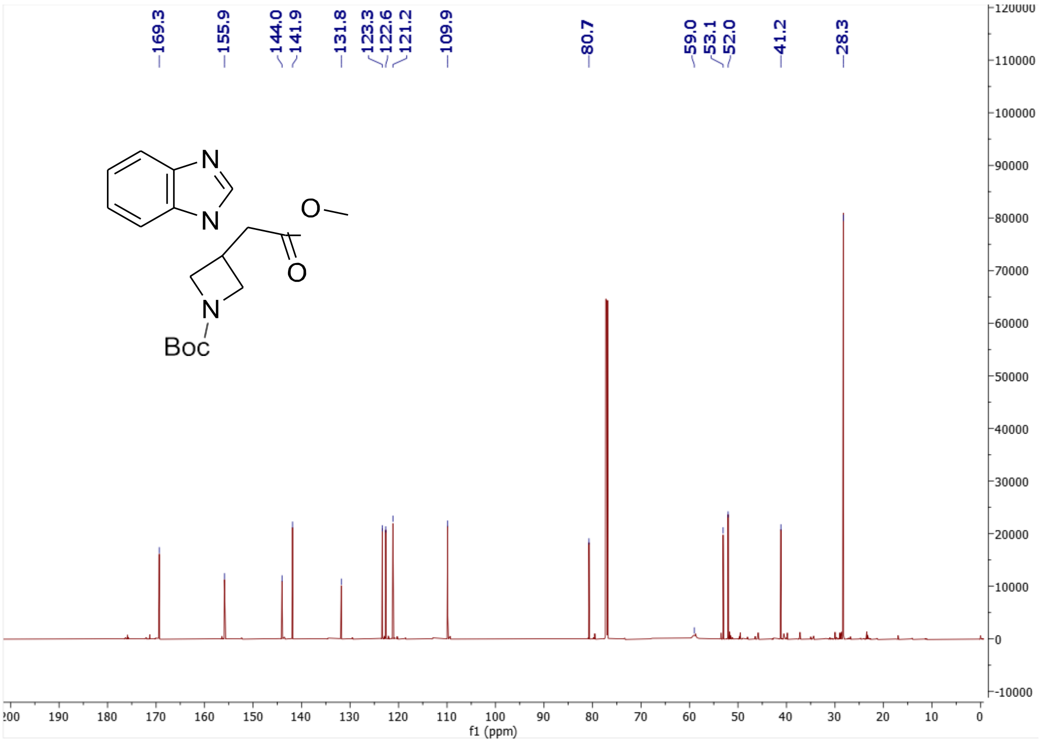

Figure S61. *tert*-Butyl 3-(1*H*-benzimidazol-1-yl)-3-(2-methoxy-2-oxoethyl)azetidine-1-carboxylate (4o) ^{13}C NMR spectrum (176 MHz, $CDCl_3$).

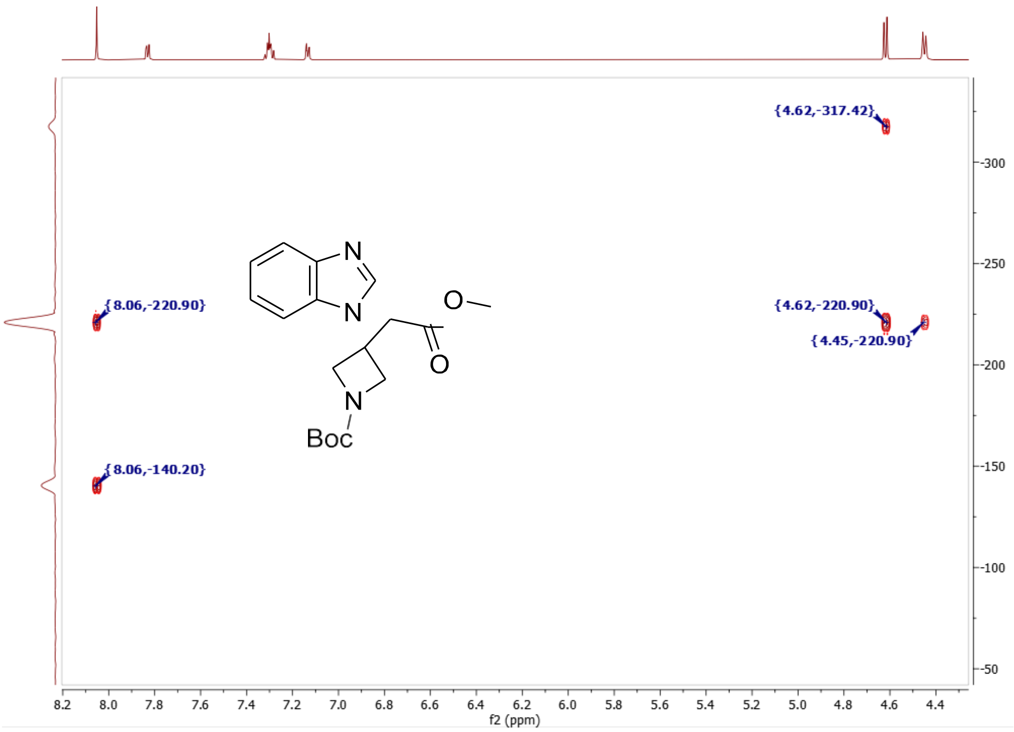

Figure S62. *tert*-Butyl 3-(1*H*-benzimidazol-1-yl)-3-(2-methoxy-2-oxoethyl)azetidine-1-carboxylate (4o) ^{1}H-^{15}N HMBC spectrum (71 MHz, $CDCl_3$).

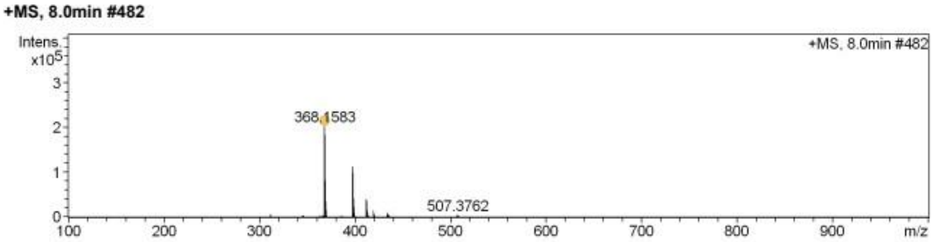

Meas. m/z	#	Ion Formula	m/z	err [ppm]	mSigma	# Sigma	Score	rdb	e⁻ Conf	N-Rule
368.1583	1	C18H23N3NaO4	368.1581	-0.5	11.4	1	100.00	8.5	even	ok

Figure S63. *tert*-Butyl 3-(1*H*-benzimidazol-1-yl)-3-(2-methoxy-2-oxoethyl)azetidine-1-carboxylate (4o) HRMS (ESI-TOF).

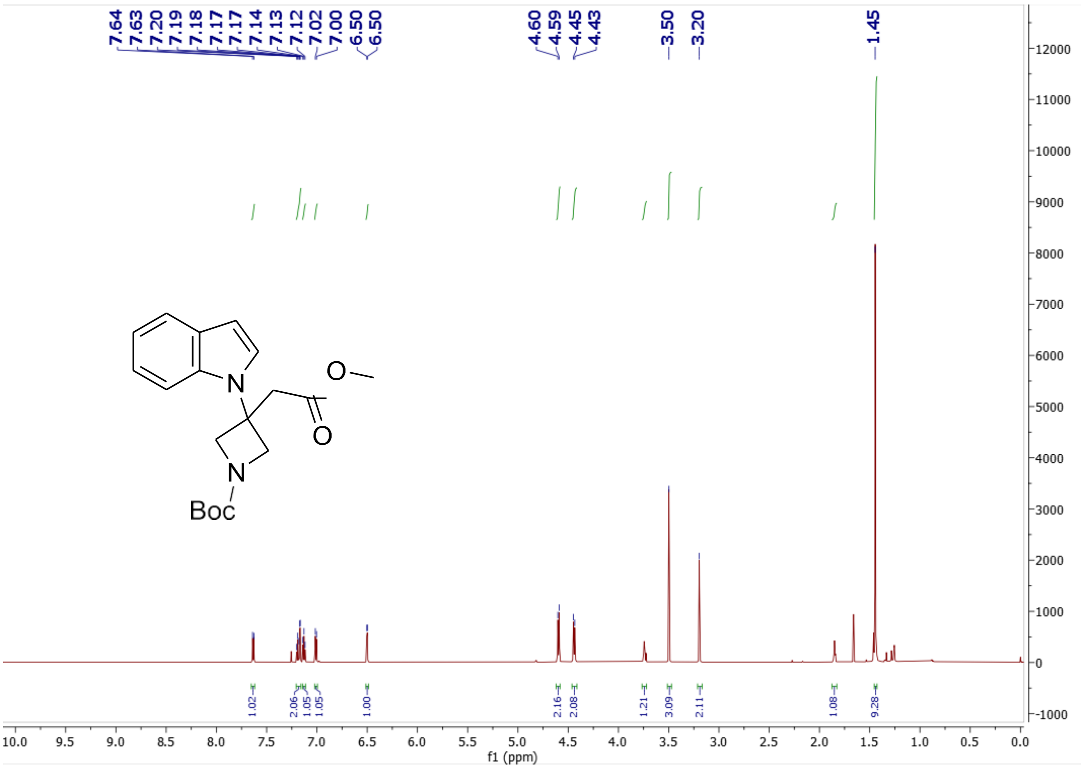

Figure S64. *tert*-Butyl 3-(1*H*-indol-1-yl)-3-(2-methoxy-2-oxoethyl)azetidine-1-carboxylate (4p) ^{1}H NMR spectrum (700 MHz, $CDCl_3$).

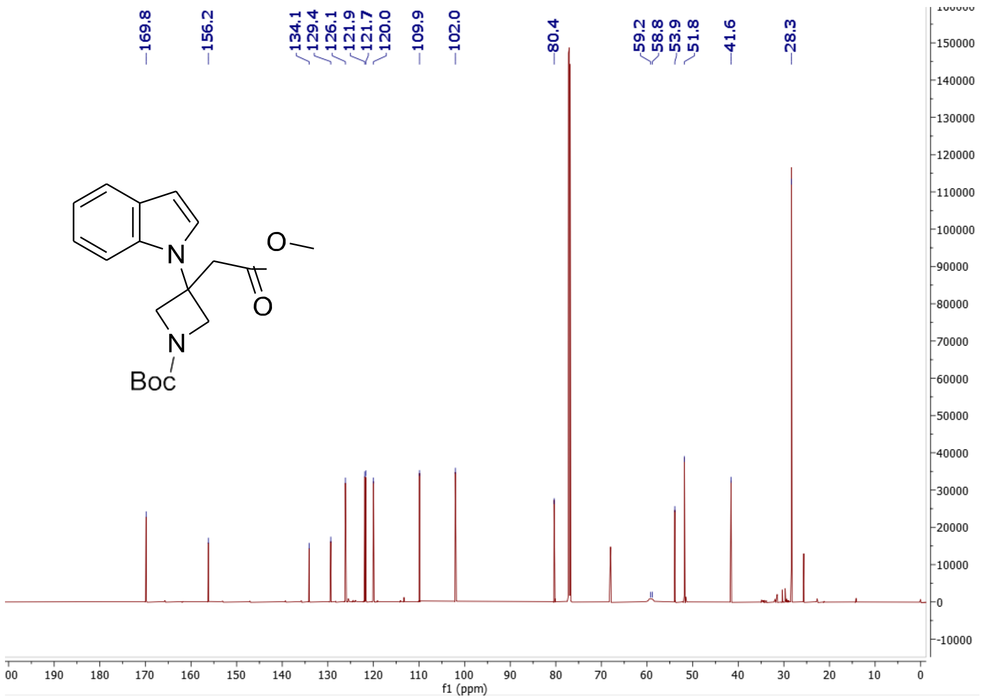

Figure S65. *tert*-Butyl 3-(1*H*-indol-1-yl)-3-(2-methoxy-2-oxoethyl)azetidine-1-carboxylate (4p) ^{13}C NMR spectrum (176 MHz, $CDCl_3$).

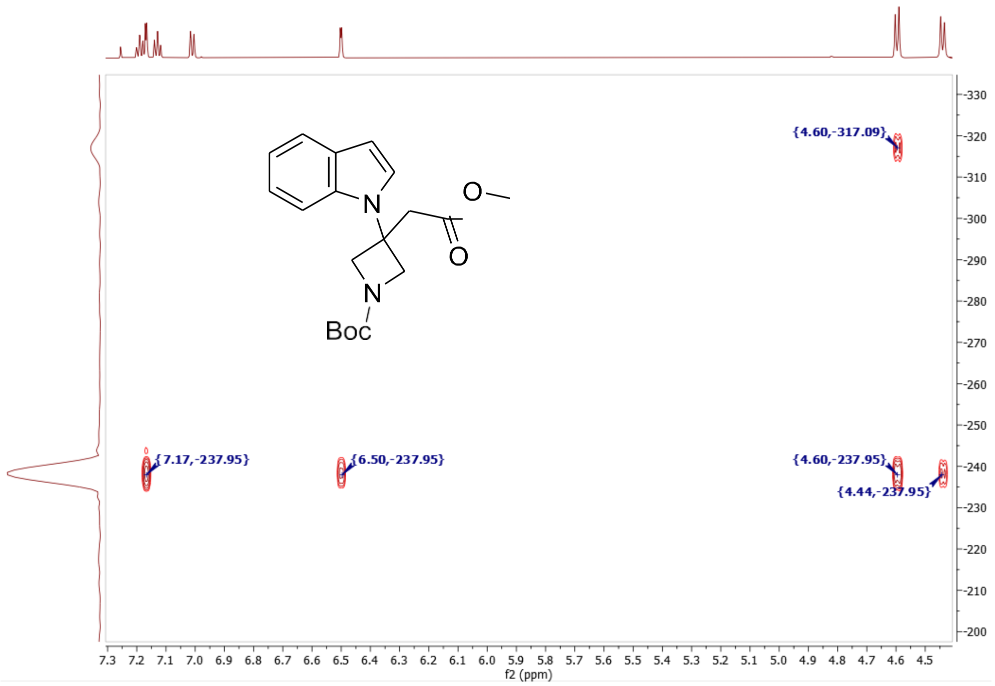

Figure S66. *tert*-Butyl 3-(1*H*-indol-1-yl)-3-(2-methoxy-2-oxoethyl)azetidine-1-carboxylate (4p) ^{1}H-^{15}N HMBC spectrum (71 MHz, $CDCl_3$).

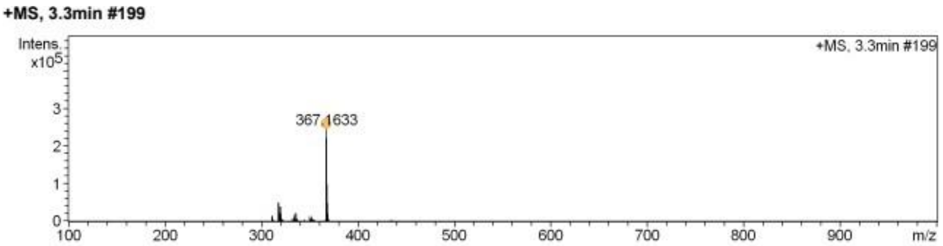

Meas. m/z	#	Ion Formula	m/z	err [ppm]	mSigma	# Sigma	Score	rdb	e⁻ Conf	N-Rule
367.1633	1	C19H24N2NaO4	367.1628	1.3	0.8	1	100.00	8.5	even	ok

Figure S67. *tert*-Butyl 3-(1*H*-indol-1-yl)-3-(2-methoxy-2-oxoethyl)azetidine-1-carboxylate (4p) HRMS (ESI-TOF).

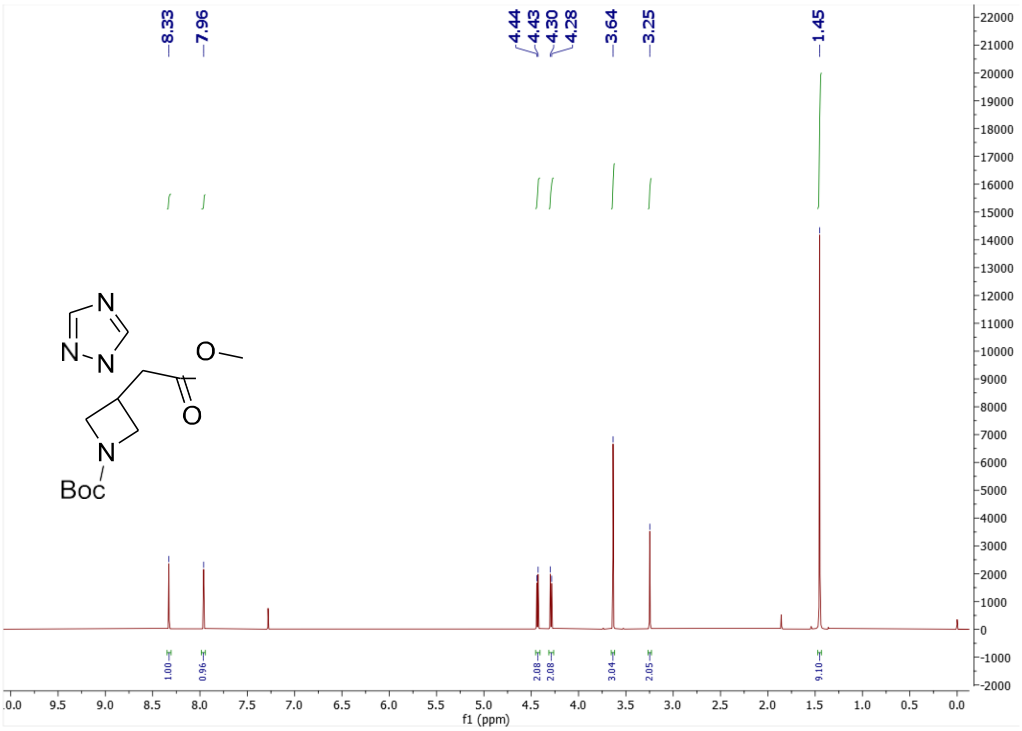

Figure S68. *tert*-Butyl 3-(2-methoxy-2-oxoethyl)-3-(1*H*-1,2,4-triazol-1-yl)azetidine-1-carboxylate (4q) ^{1}H NMR spectrum (700 MHz, $CDCl_3$).

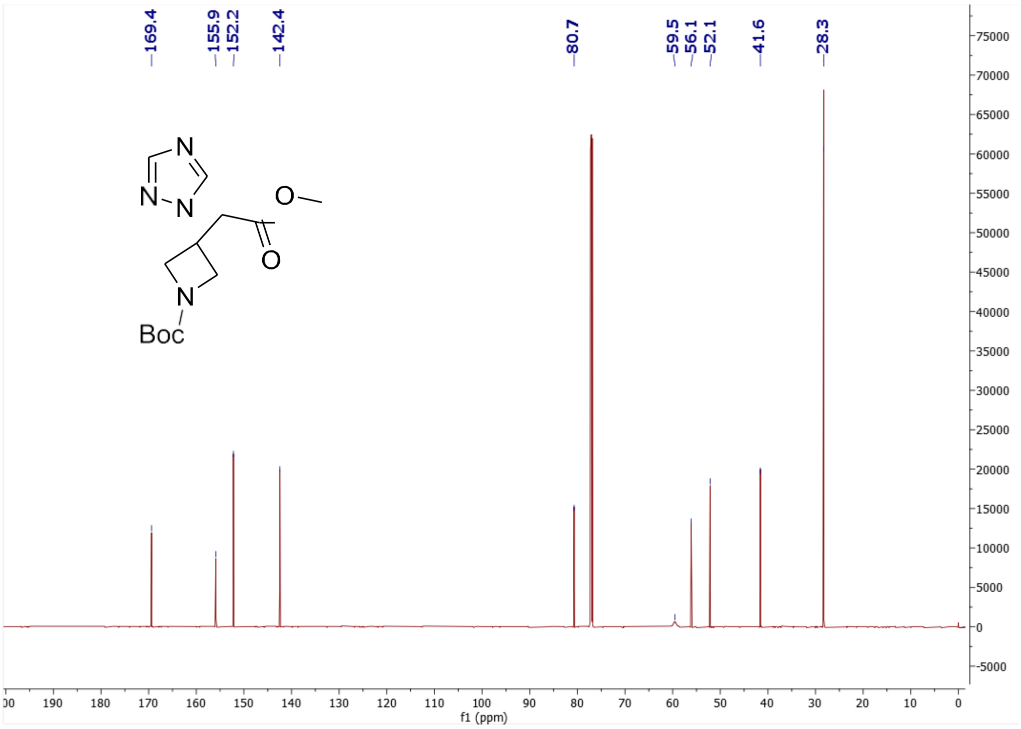

Figure S69. *tert*-Butyl 3-(2-methoxy-2-oxoethyl)-3-(1*H*-1,2,4-triazol-1-yl)azetidine-1-carboxylate (4q) ^{13}C NMR spectrum (176 MHz, $CDCl_3$).

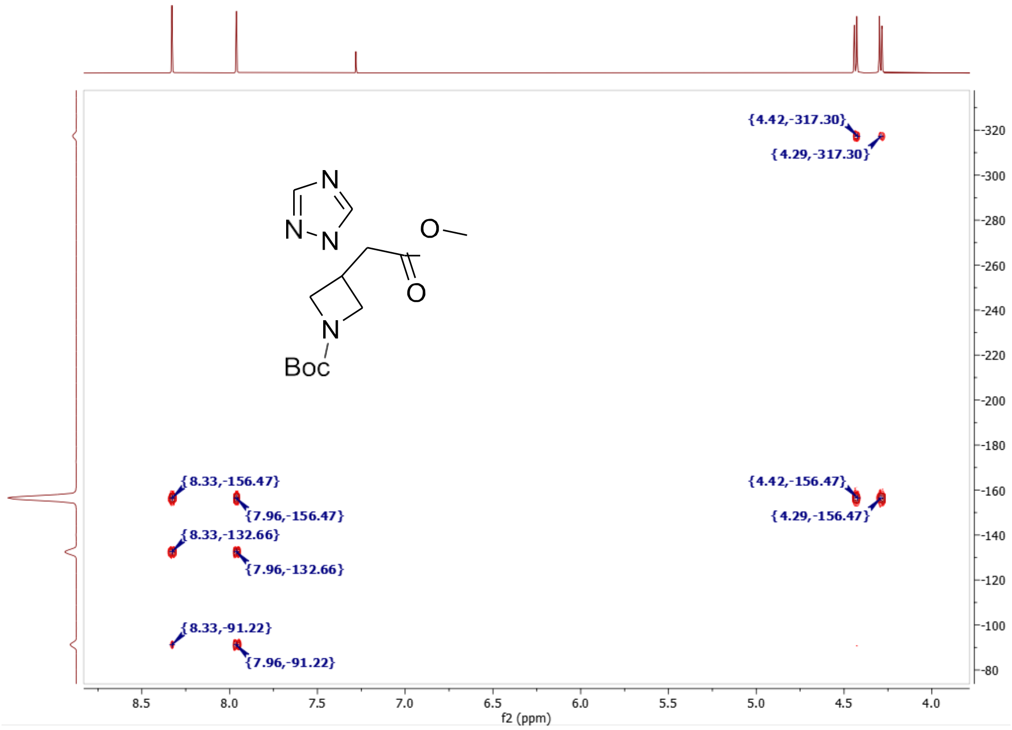

Figure S70. *tert*-Butyl 3-(2-methoxy-2-oxoethyl)-3-(1*H*-1,2,4-triazol-1-yl)azetidine-1-carboxylate (4q) ^{1}H-^{15}N HMBC spectrum (71 MHz, $CDCl_3$).

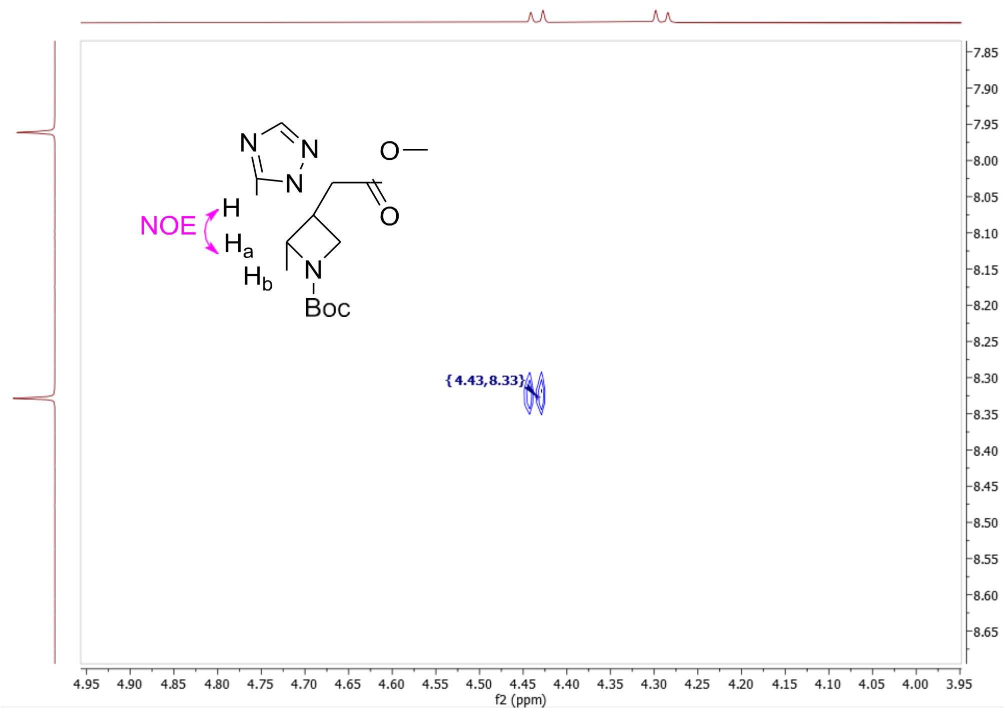

Figure S71. *tert*-Butyl 3-(2-methoxy-2-oxoethyl)-3-(1*H*-1,2,4-triazol-1-yl)azetidine-1-carboxylate (4q) ^{1}H-^{1}H NOESY NMR spectrum (700 MHz, $CDCl_3$).

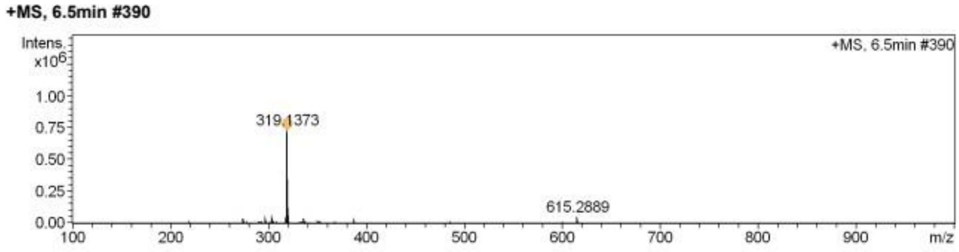

Meas. m/z	#	Ion Formula	m/z	err [ppm]	mSigma	# Sigma	Score	rdb	e⁻ Conf	N-Rule
319.1373	1	C13H20N4NaO4	319.1377	1.1	8.2	1	100.00	5.5	even	ok

Figure S72. *tert*-Butyl 3-(2-methoxy-2-oxoethyl)-3-(1*H*-1,2,4-triazol-1-yl)azetidine-1-carboxylate (4q) HRMS (ESI-TOF).

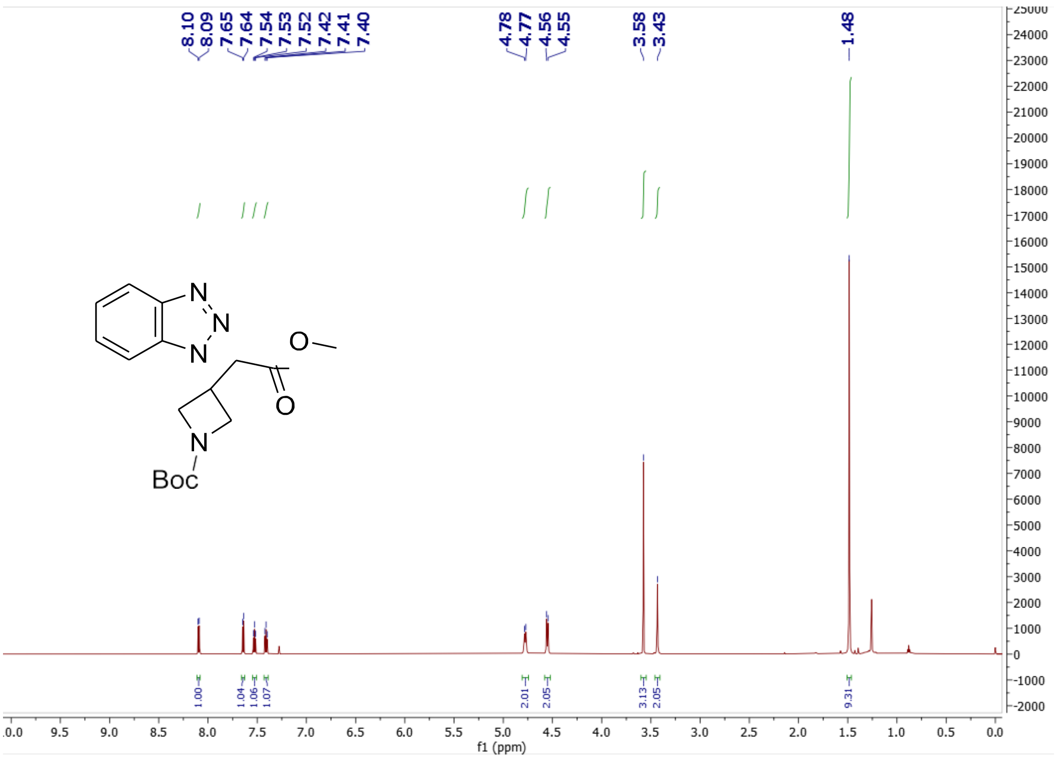

Figure S73. *tert*-Butyl 3-(2-methoxy-2-oxoethyl)-3-(1*H*-1,2,4-triazol-1-yl)azetidine-1-carboxylate (4r) ^{1}H NMR spectrum (700 MHz, $CDCl_3$).

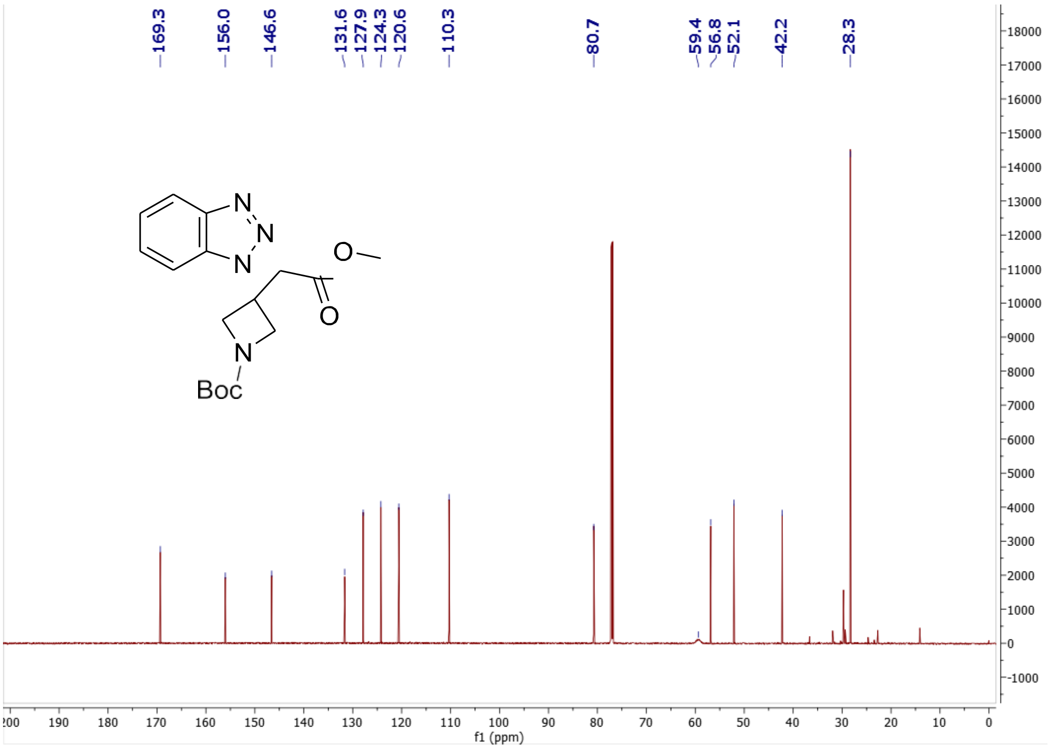

Figure S74. *tert*-Butyl 3-(1*H*-benzotriazol-1-yl)-3-(2-methoxy-2-oxoethyl)azetidine-1-carboxylate (4r) ^{13}C NMR spectrum (176 MHz, $CDCl_3$).

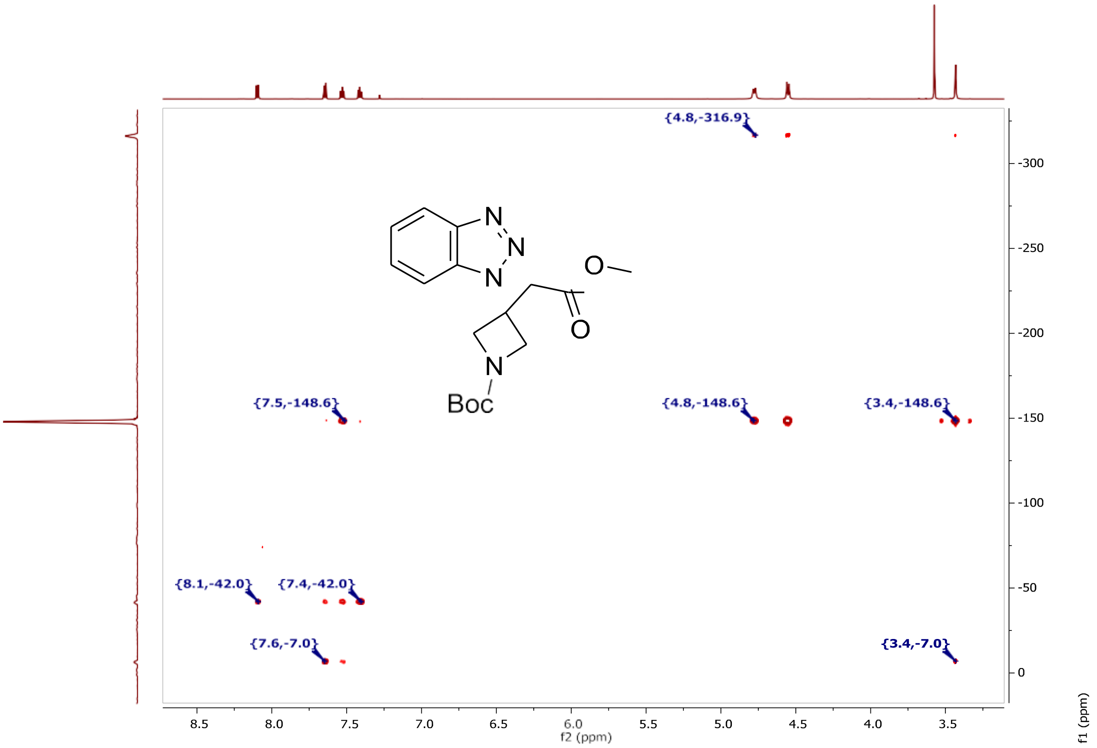

Figure S75. *tert*-Butyl 3-(1*H*-benzotriazol-1-yl)-3-(2-methoxy-2-oxoethyl)azetidine-1-carboxylate (4r) ^{1}H-^{15}N HMBC spectrum (71 MHz, $CDCl_3$).

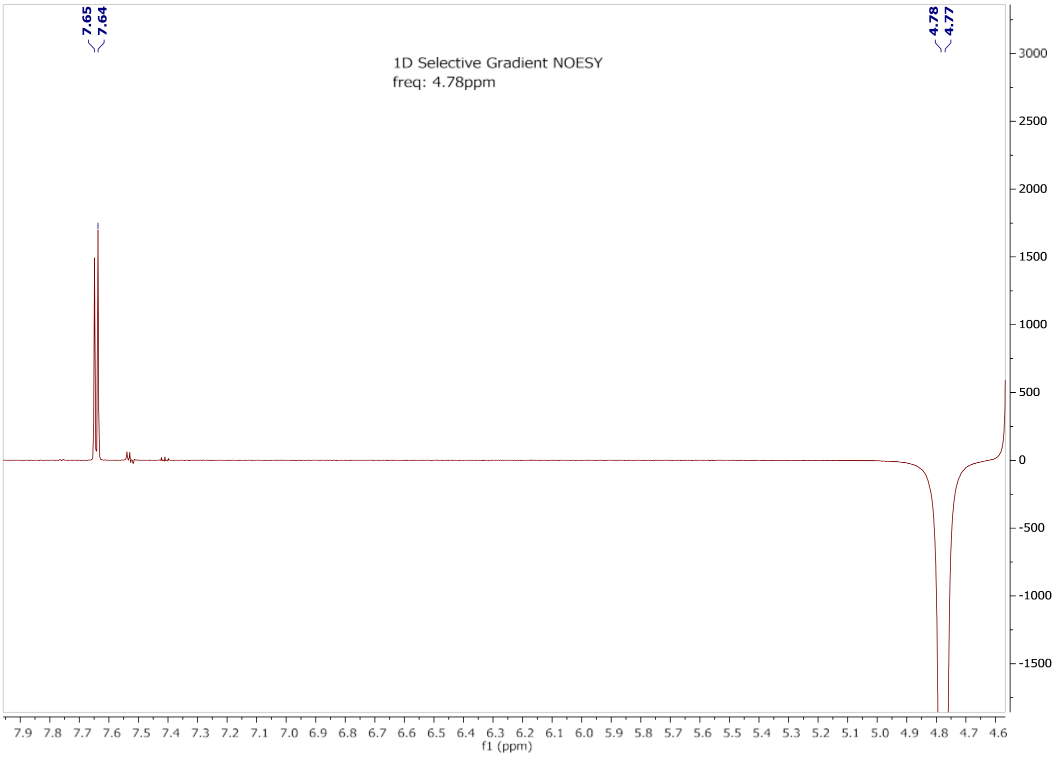

Figure S76. *tert*-Butyl 3-(1*H*-benzotriazol-1-yl)-3-(2-methoxy-2-oxoethyl)azetidine-1-carboxylate (4r) 1D Selective Gradient NOESY NMR spectrum (700 MHz, $CDCl_3$).

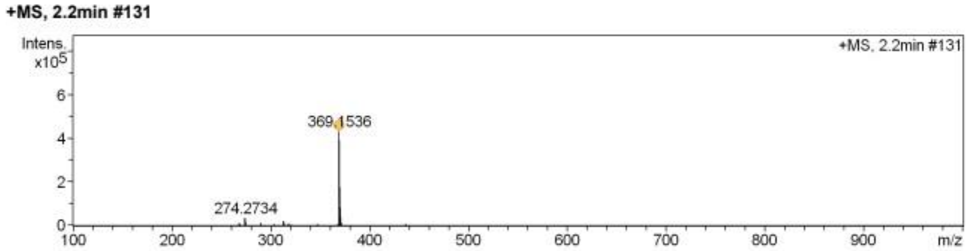

Meas. m/z	#	Ion Formula	m/z	err [ppm]	mSigma	# Sigma	Score	rdb	e⁻ Conf	N-Rule
369.1536	1	C17H22N4NaO4	369.1533	-0.7	6.4	1	100.00	8.5	even	ok

Figure S77. *tert*-Butyl 3-(1*H*-benzotriazol-1-yl)-3-(2-methoxy-2-oxoethyl)azetidine-1-carboxylate (4r) HRMS (ESI-TOF).

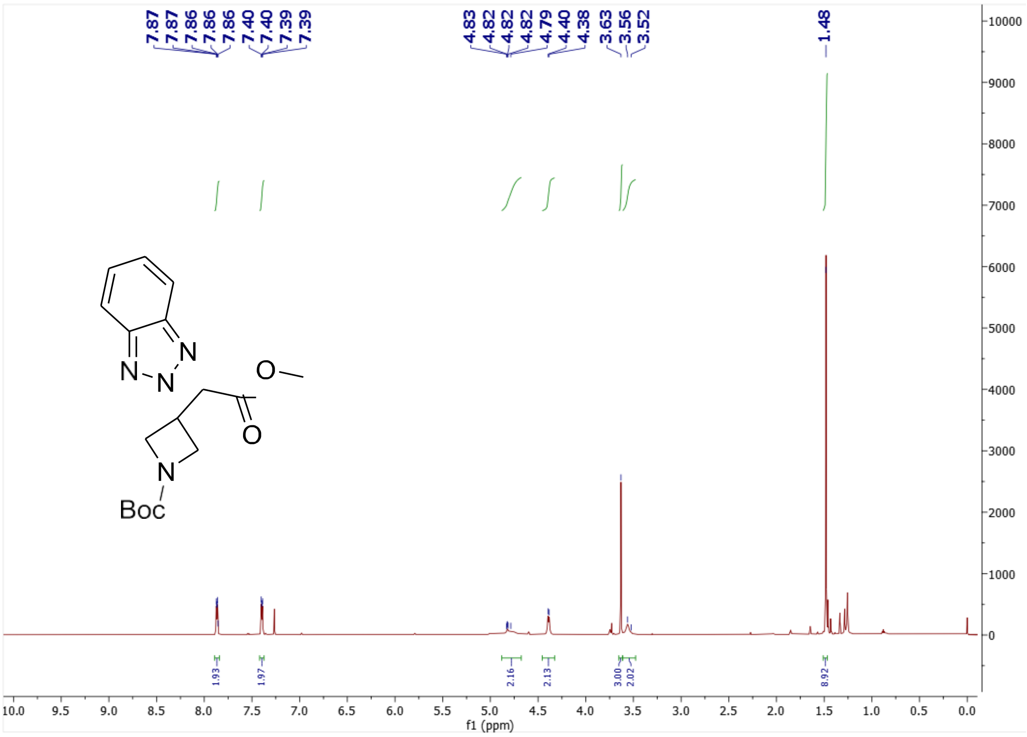

Figure S78. *tert*-Butyl 3-(2*H*-benzotriazol-2-yl)-3-(2-methoxy-2-oxoethyl)azetidine-1-carboxylate (4s) ^{1}H NMR spectrum (700 MHz, $CDCl_3$).

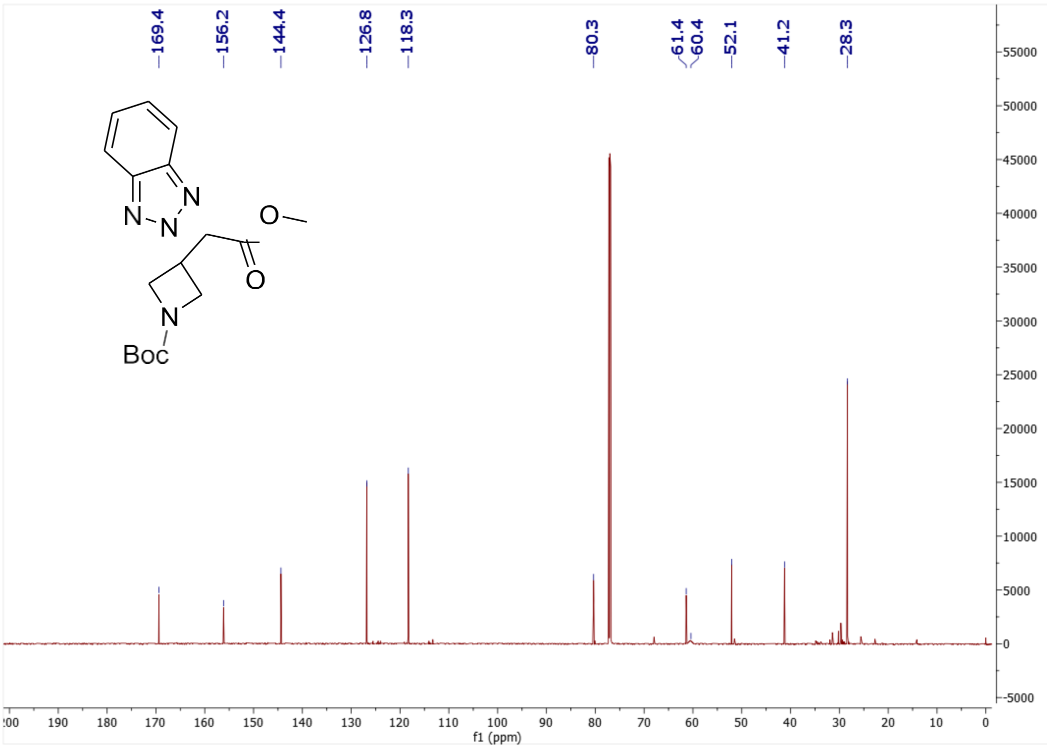

Figure S79. *tert*-Butyl 3-(2*H*-benzotriazol-2-yl)-3-(2-methoxy-2-oxoethyl)azetidine-1-carboxylate (4s) ^{13}C NMR spectrum (176 MHz, $CDCl_3$).

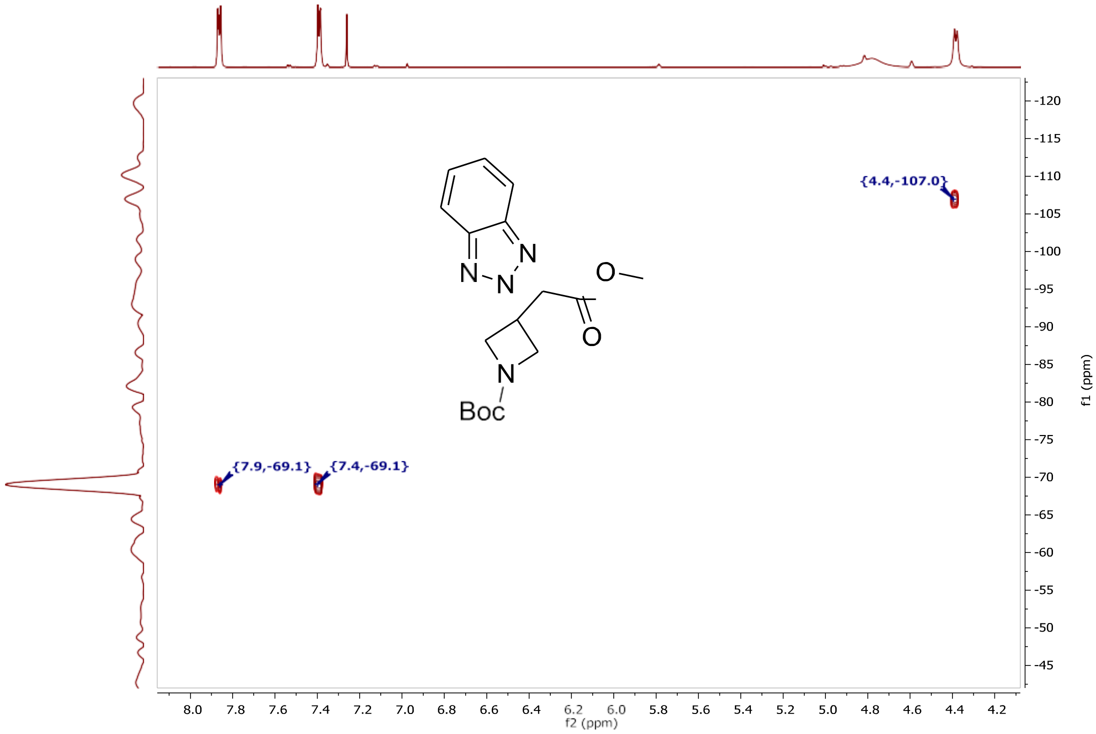

Figure S80. *tert*-Butyl 3-(2*H*-benzotriazol-2-yl)-3-(2-methoxy-2-oxoethyl)azetidine-1-carboxylate (4s) ^{1}H-^{15}N HMBC spectrum (71 MHz, $CDCl_3$).

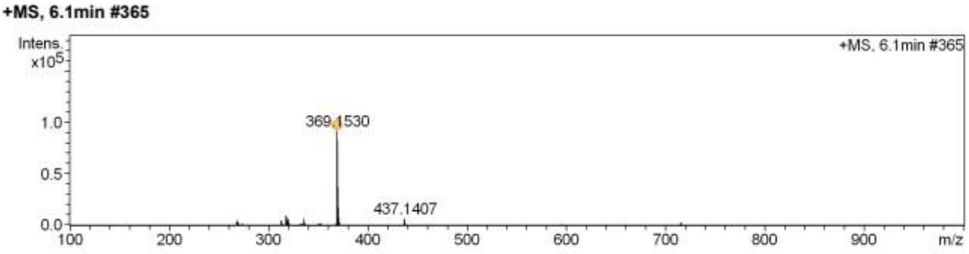

Meas. m/z	#	Ion Formula	m/z	err [ppm]	mSigma	# Sigma	Score	rdb	e⁻ Conf	N-Rule
369.1530	1	C17H22N4NaO4	369.1533	-0.9	2.5	1	100.00	8.5	even	ok

Figure S81. *tert*-Butyl 3-(2*H*-benzotriazol-2-yl)-3-(2-methoxy-2-oxoethyl)azetidine-1-carboxylate (4s) HRMS (ESI-TOF).

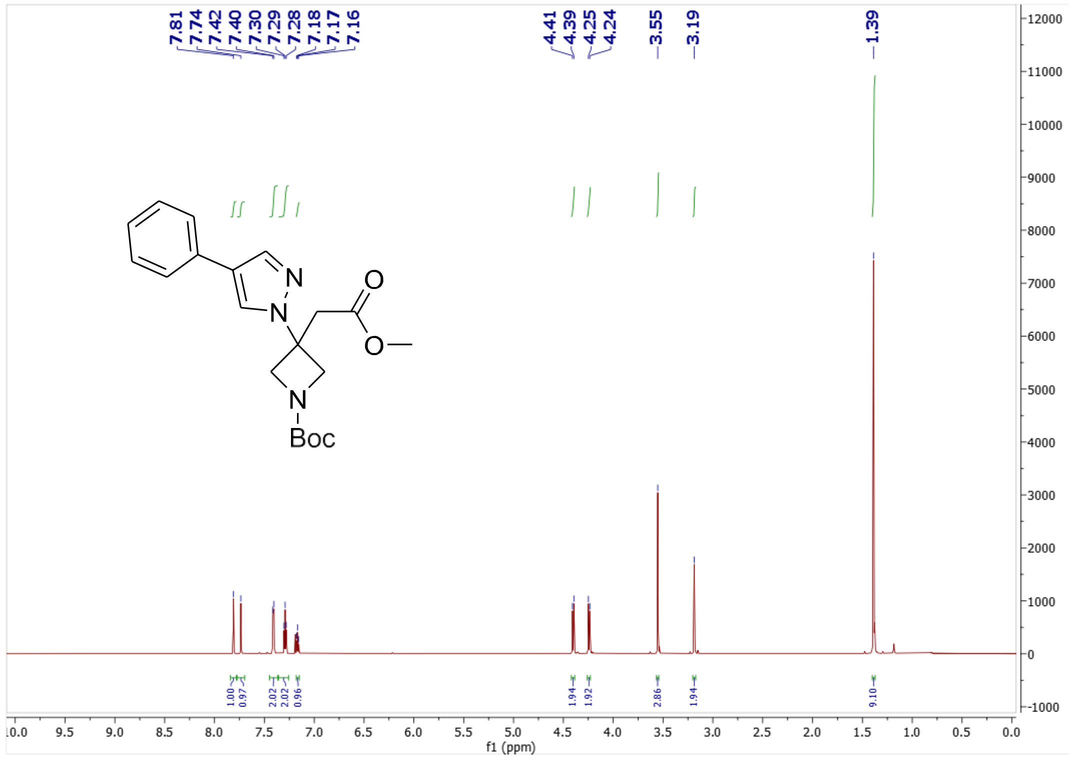

Figure S82. *tert*-Butyl 3-(2-methoxy-2-oxoethyl)-3-(4-phenyl-1*H*-pyrazol-1-yl)azetidine-1-carboxylate (5a) ^{1}H NMR spectrum (700 MHz, $CDCl_3$).

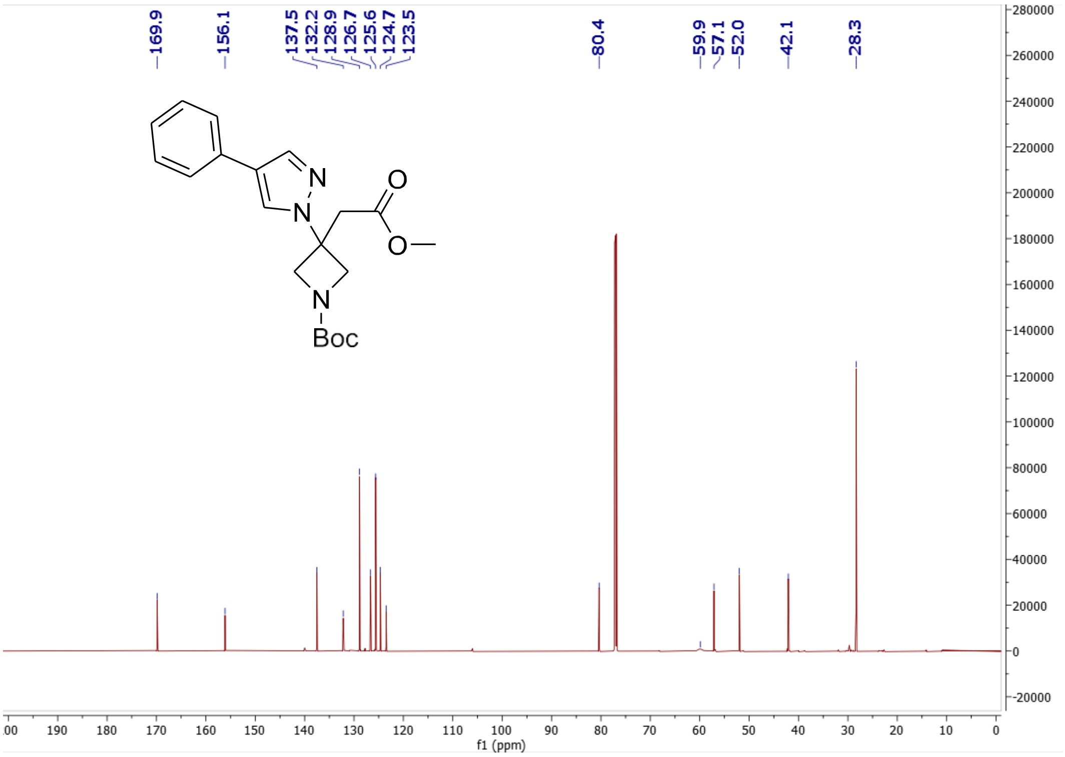

Figure S83. *tert*-Butyl 3-(2-methoxy-2-oxoethyl)-3-(4-phenyl-1*H*-pyrazol-1-yl)azetidine-1- carboxylate (5a) ^{13}C NMR spectrum (176 MHz, $CDCl_3$).

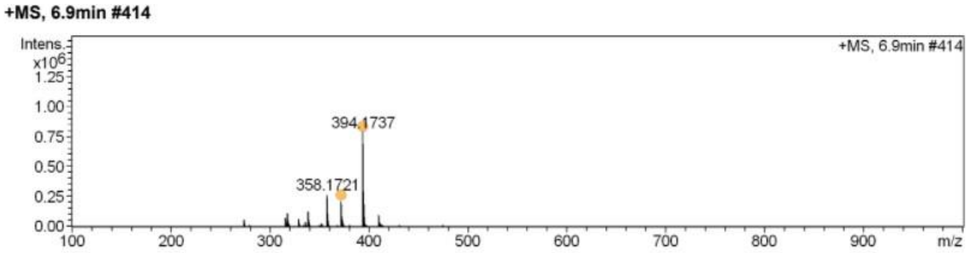

Meas. m/z	#	Ion Formula	m/z	err [ppm]	mSigma	# Sigma	Score	rdb	e⁻ Conf	N-Rule
372.1901	1	C20H26N3O4	372.1918	4.5	17.1	1	100.00	9.5	even	ok
394.1737	1	C20H25N3NaO4	394.1737	-0.0	18.9	1	100.00	9.5	even	ok

Figure S84. *tert*-Butyl 3-(2-methoxy-2-oxoethyl)-3-(4-phenyl-1*H*-pyrazol-1-yl)azetidine-1- carboxylate (5a) HRMS (ESI-TOF).

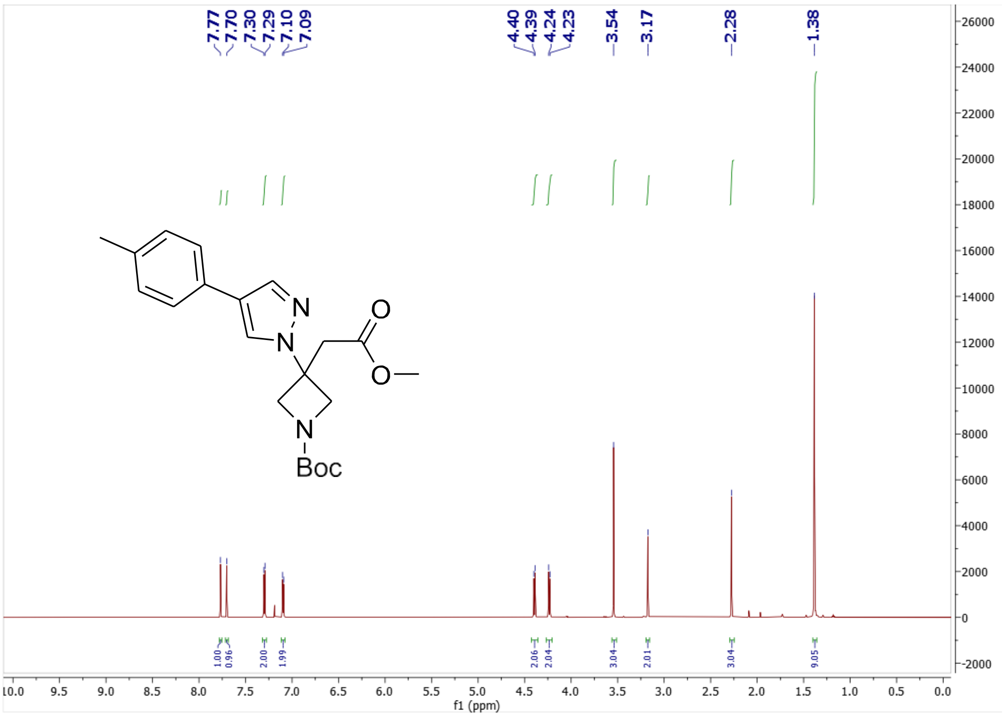

Figure S85. *tert*-Butyl 3-(2-methoxy-2-oxoethyl)-3-[4-(4-methylphenyl)-1*H*-pyrazol-1-yl]azetidine-1- carboxylate (5b) ^{1}H NMR spectrum (700 MHz, $CDCl_3$).

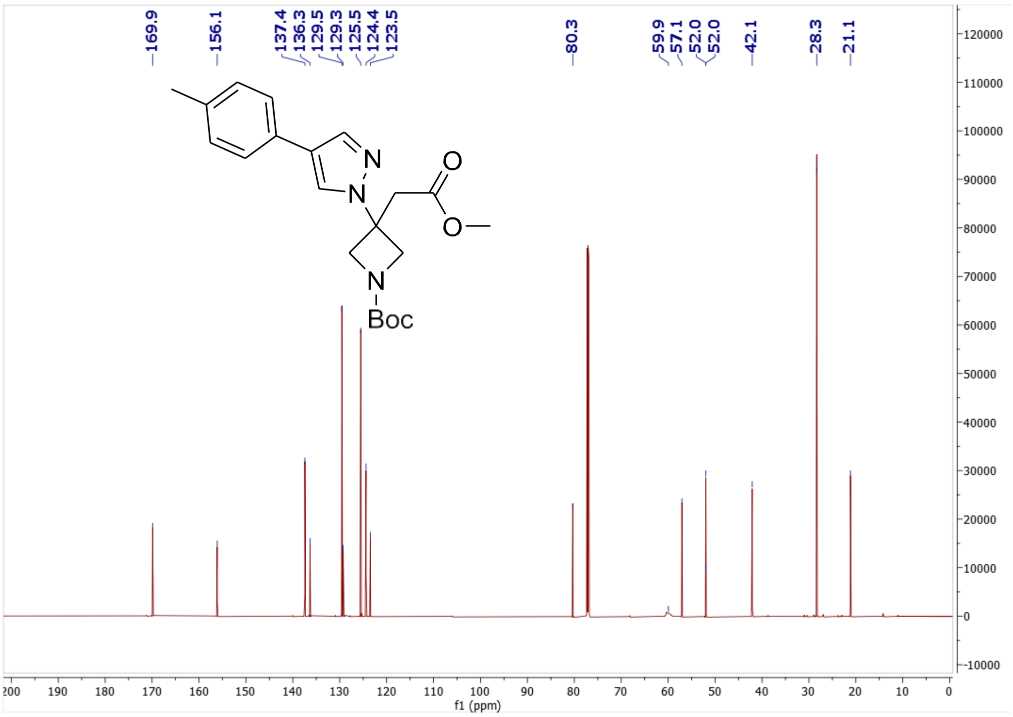

Figure S86. *tert*-Butyl 3-(2-methoxy-2-oxoethyl)-3-[4-(4-methylphenyl)-1*H*-pyrazol-1-yl]azetidine-1- carboxylate (5b) ^{13}C NMR spectrum (176 MHz, $CDCl_3$).

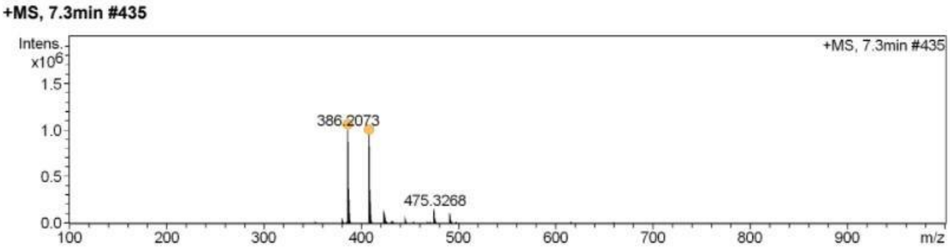

Meas. m/z	#	Ion Formula	m/z	err [ppm]	mSigma	# Sigma	Score	rdb	e⁻ Conf	N-Rule
386.2073	1	C21H28N3O4	386.2074	-0.4	22.5	1	100.00	9.5	even	ok
408.1894	1	C21H27N3NaO4	408.1894	0.0	16.3	1	100.00	9.5	even	ok

Figure S87. *tert*-Butyl 3-(2-methoxy-2-oxoethyl)-3-[4-(4-methylphenyl)-1*H*-pyrazol-1-yl]azetidine-1-carboxylate (5b) HRMS (ESI-TOF).

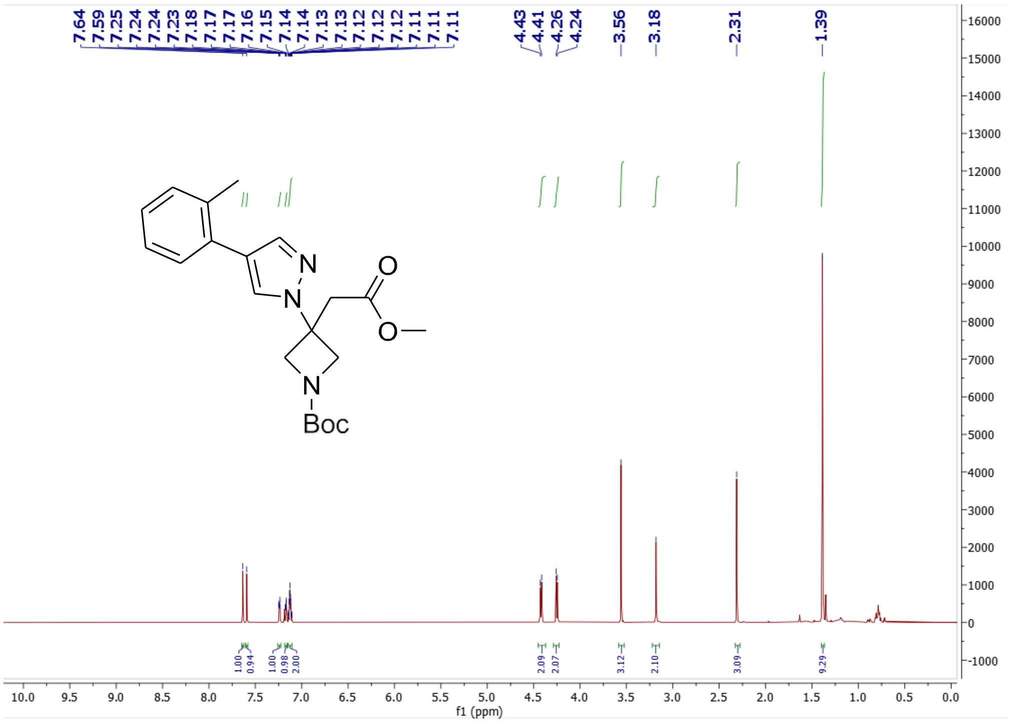

Figure S88. *tert*-Butyl 3-(2-methoxy-2-oxoethyl)-3-[4-(2-methylphenyl)-1*H*-pyrazol-1-yl]azetidine-1-carboxylate (5c) ^{1}H NMR spectrum (700 MHz, $CDCl_3$).

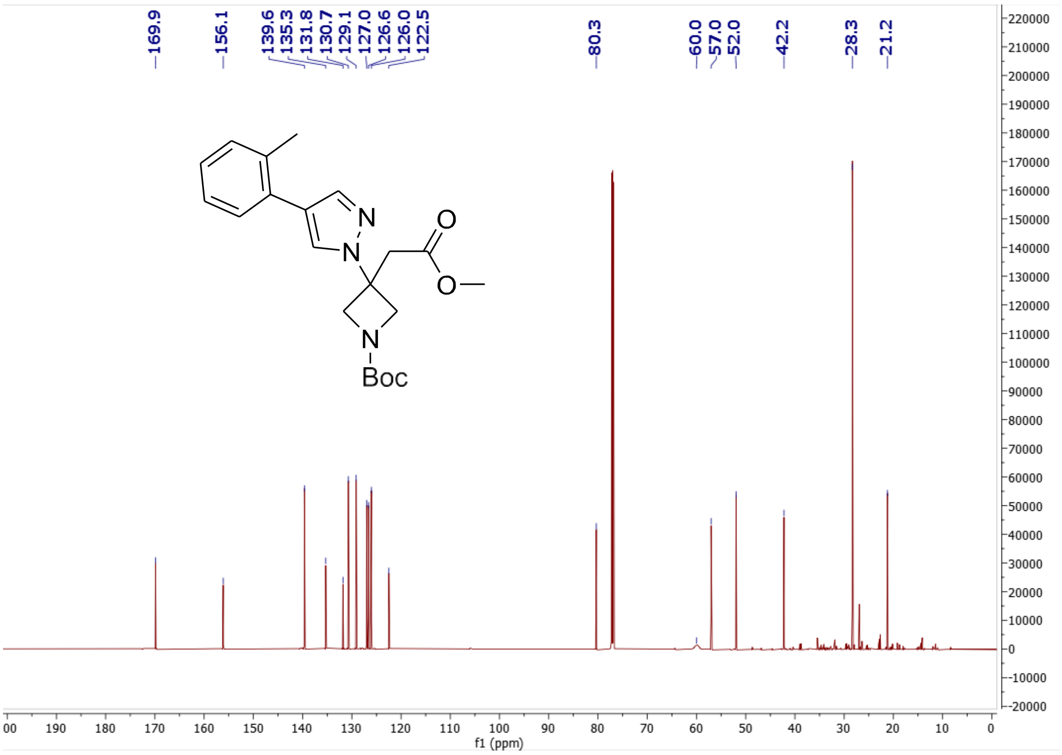

Figure S89. *tert*-Butyl 3-(2-methoxy-2-oxoethyl)-3-[4-(2-methylphenyl)-1*H*-pyrazol-1-yl]azetidine-1-carboxylate (5c) ^{13}C NMR spectrum (176 MHz, $CDCl_3$).

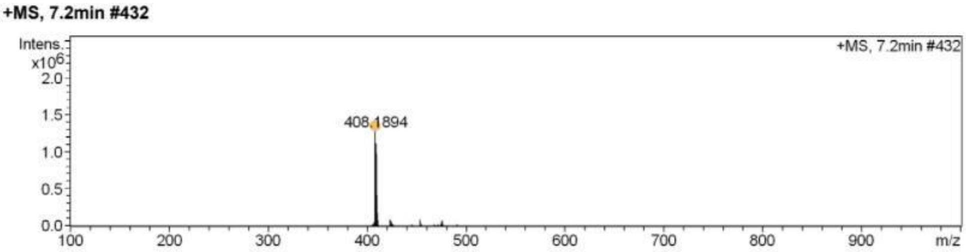

Meas. m/z	#	Ion Formula	m/z	err [ppm]	mSigma	# Sigma	Score	rdb	e⁻ Conf	N-Rule
408.1894	1	C21H27N3NaO4	408.1894	0.1	324.6	1	100.00	9.5	even	ok

Figure S90. *tert*-Butyl 3-(2-methoxy-2-oxoethyl)-3-[4-(2-methylphenyl)-1*H*-pyrazol-1-yl]azetidine-1-carboxylate (5c) HRMS (ESI-TOF).

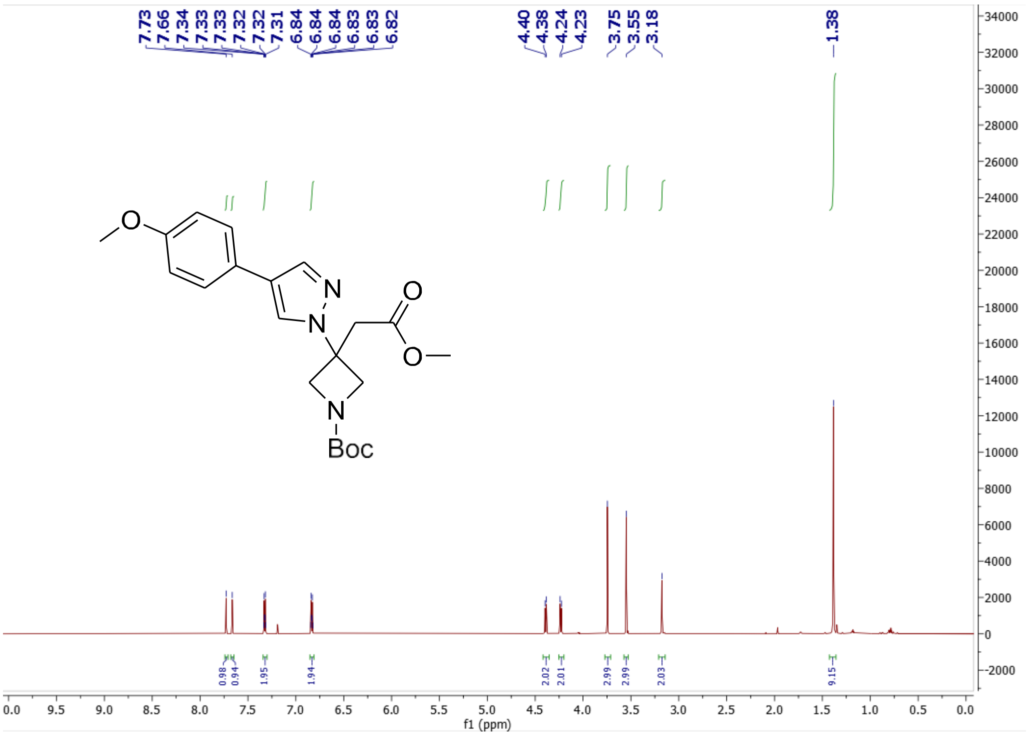

Figure S91. *tert*-Butyl 3-(2-methoxy-2-oxoethyl)-3-[4-(4-methoxyphenyl)-1*H*-pyrazol-1-yl]azetidine-1-carboxylate (5d) ^{1}H NMR spectrum (700 MHz, $CDCl_3$).

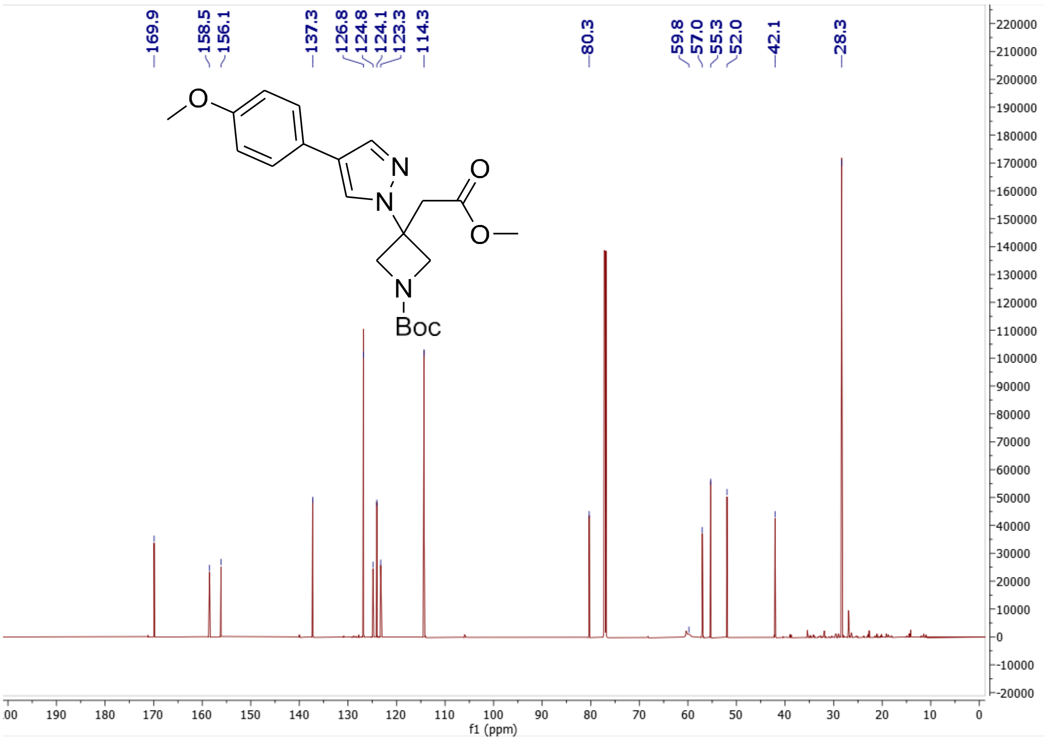

Figure S92. *tert*-Butyl 3-(2-methoxy-2-oxoethyl)-3-[4-(4-methoxyphenyl)-1*H*-pyrazol-1-yl]azetidine-1-carboxylate (5d) ^{13}C NMR spectrum (176 MHz, $CDCl_3$).

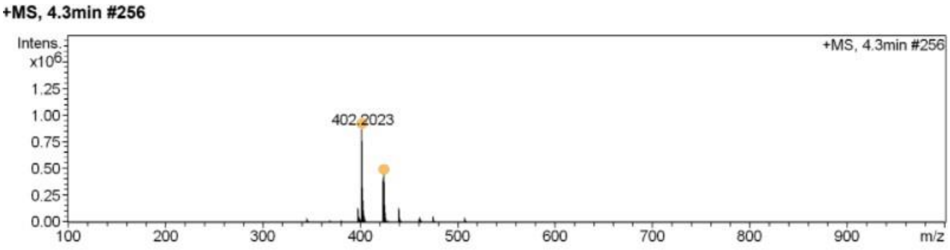

Meas. m/z	#	Ion Formula	m/z	err [ppm]	mSigma	# Sigma	Score	rdb	e⁻ Conf	N-Rule
402.2023	1	C21H28N3O5	402.2023	0.2	11.1	1	100.00	9.5	even	ok
424.1841	1	C21H27N3NaO5	424.1843	0.4	2.3	1	100.00	9.5	even	ok

Figure S93. *tert*-Butyl 3-(2-methoxy-2-oxoethyl)-3-[4-(4-methoxyphenyl)-1*H*-pyrazol-1-yl]azetidine-1-carboxylate (5d) HRMS (ESI-TOF).

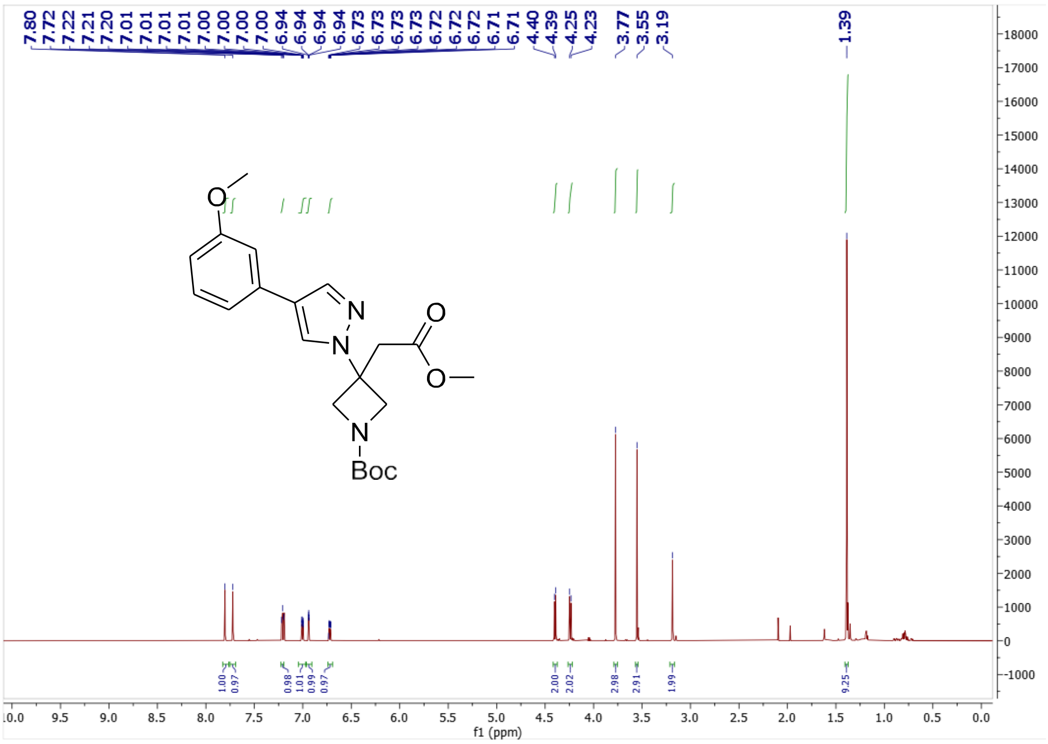

Figure S94. *tert*-Butyl 3-(2-methoxy-2-oxoethyl)-3-[4-(3-methoxyphenyl)-1*H*-pyrazol-1-yl]azetidine-1-carboxylate (5e) ^{1}H NMR spectrum (700 MHz, $CDCl_3$).

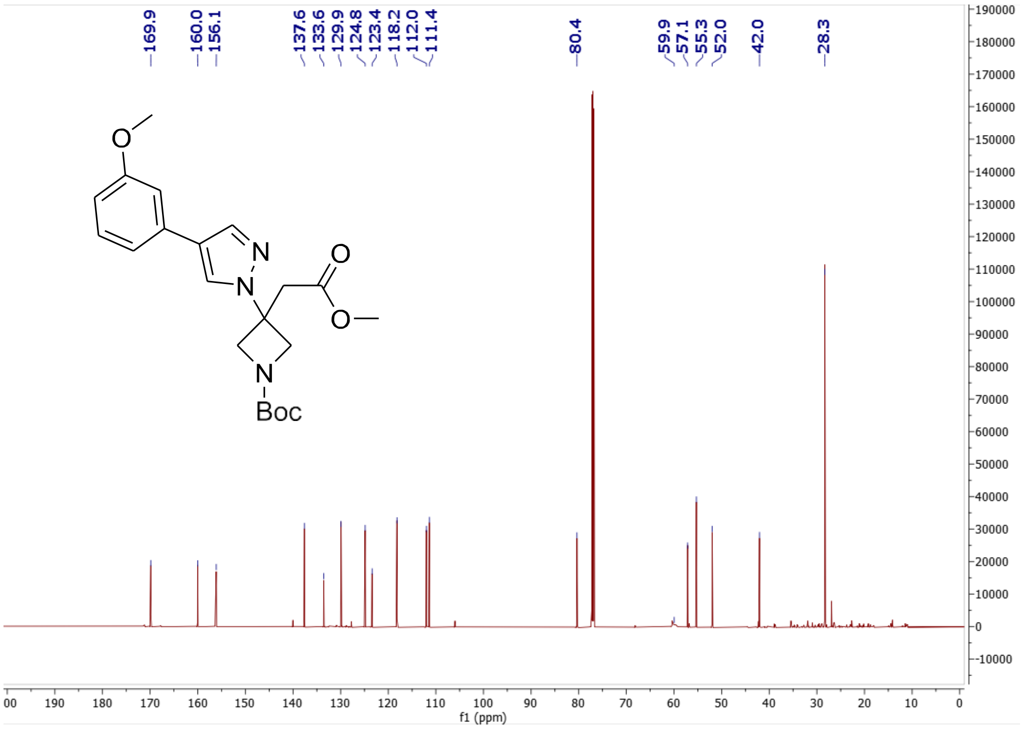

Figure S95. *tert*-Butyl 3-(2-methoxy-2-oxoethyl)-3-[4-(3-methoxyphenyl)-1*H*-pyrazol-1-yl]azetidine-1-carboxylate (5e) ^{13}C NMR spectrum (176 MHz, $CDCl_3$).

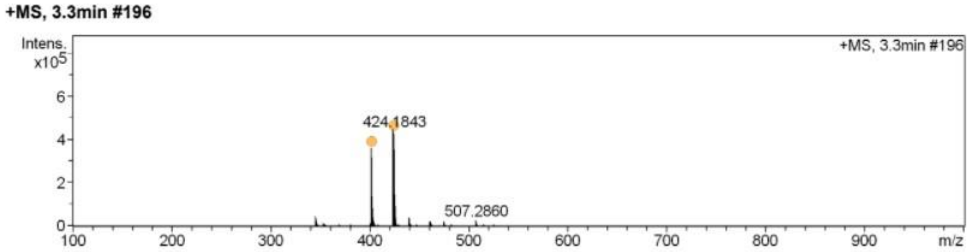

Meas. m/z	#	Ion Formula	m/z	err [ppm]	mSigma	# Sigma	Score	rdb	e⁻ Conf	N-Rule
402.2016	1	C21H28N3O5	402.2023	-1.9	5.6	1	100.00	9.5	even	ok
424.1843	1	C21H27N3NaO5	424.1843	0.1	4.1	1	100.00	9.5	even	ok

Figure S96. *tert*-Butyl 3-(2-methoxy-2-oxoethyl)-3-[4-(3-methoxyphenyl)-1*H*-pyrazol-1-yl]azetidine-1-carboxylate (5e) HRMS (ESI-TOF).

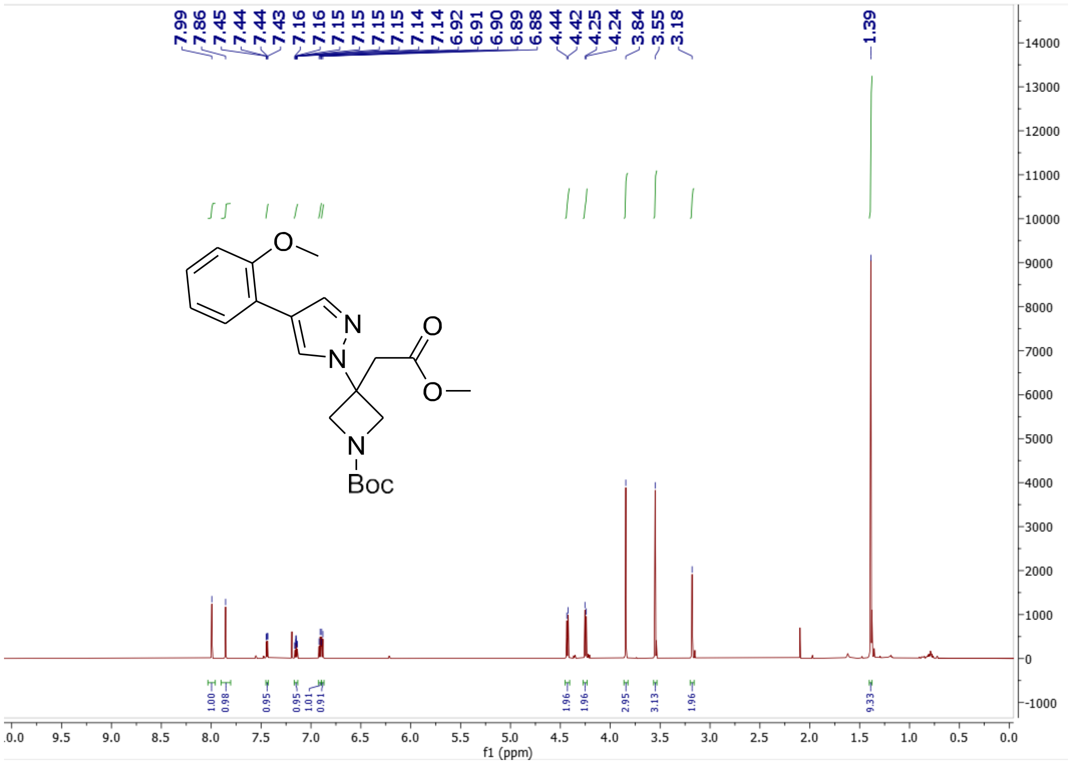

Figure S97. *tert*-Butyl3-(2-methoxy-2-oxoethyl)-3-[4-(2-methoxyphenyl)-1*H*-pyrazol-1-yl]azetidine-1-carboxylate (5f) ^{1}H NMR spectrum (700 MHz, $CDCl_3$).

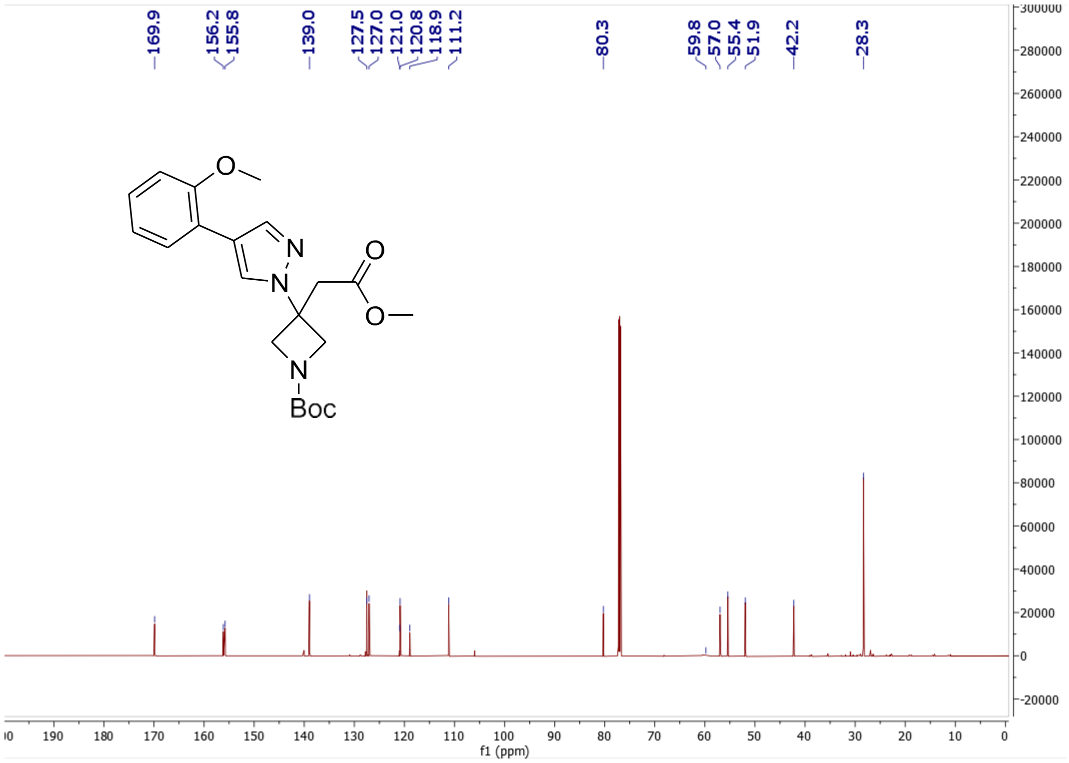

Figure S98. *tert*-Butyl3-(2-methoxy-2-oxoethyl)-3-[4-(2-methoxyphenyl)-1*H*-pyrazol-1-yl]azetidine-1-carboxylate (5f) ^{13}C NMR spectrum (176 MHz, $CDCl_3$).

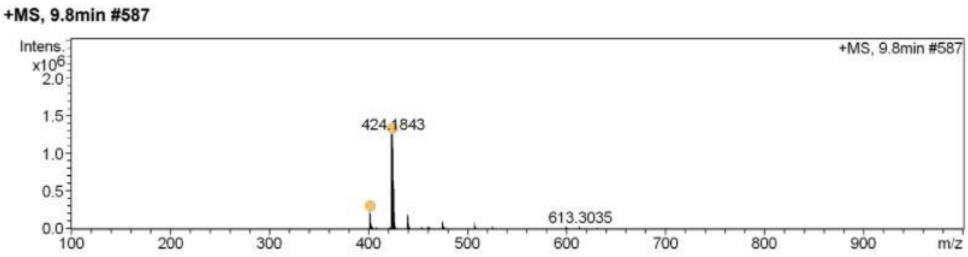

Meas. m/z	#	Ion Formula	m/z	err [ppm]	mSigma	# Sigma	Score	rdb	e⁻ Conf	N-Rule
402.2005	1	C21H28N3O5	402.2023	4.5	19.7	1	100.00	9.5	even	ok
424.1843	1	C21H27N3NaO5	424.1843	0.1	135.4	1	100.00	9.5	even	ok

Figure S99. *tert*-Butyl 3-(2-methoxy-2-oxoethyl)-3-[4-(2-methoxyphenyl)-1*H*-pyrazol-1-yl]azetidine-1-carboxylate (5f) HRMS (ESI-TOF).

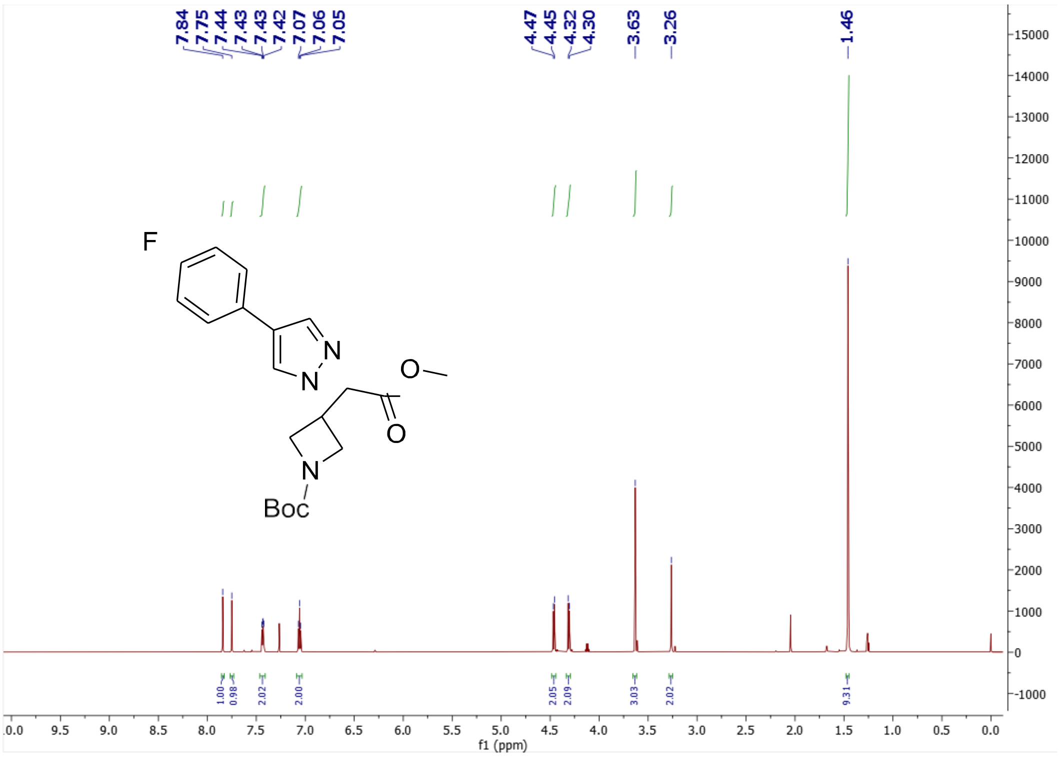

Figure S100. *tert*-Butyl 3-[4-(4-fluorophenyl)-1*H*-pyrazol-1-yl]-3-(2-methoxy-2-oxoethyl)azetidine-1- carboxylate (5g) ^{1}H NMR spectrum (700 MHz, $CDCl_3$).

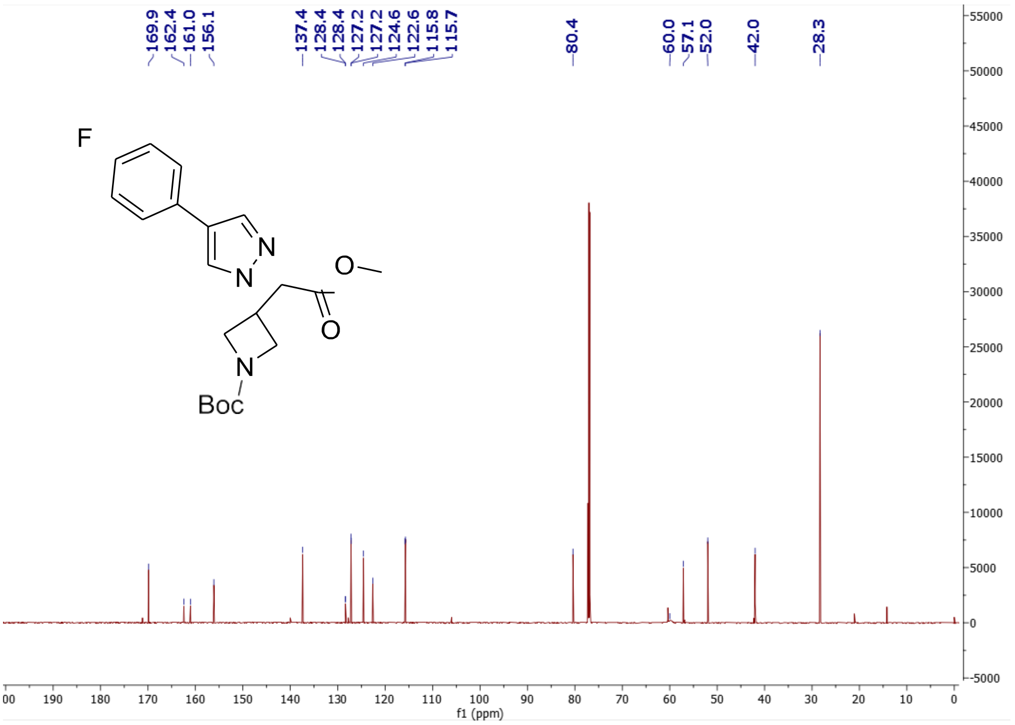

Figure S101. *tert*-Butyl 3-[4-(4-fluorophenyl)-1*H*-pyrazol-1-yl]-3-(2-methoxy-2-oxoethyl)azetidine-1- carboxylate (5g) ^{13}C NMR spectrum (176 MHz, $CDCl_3$).

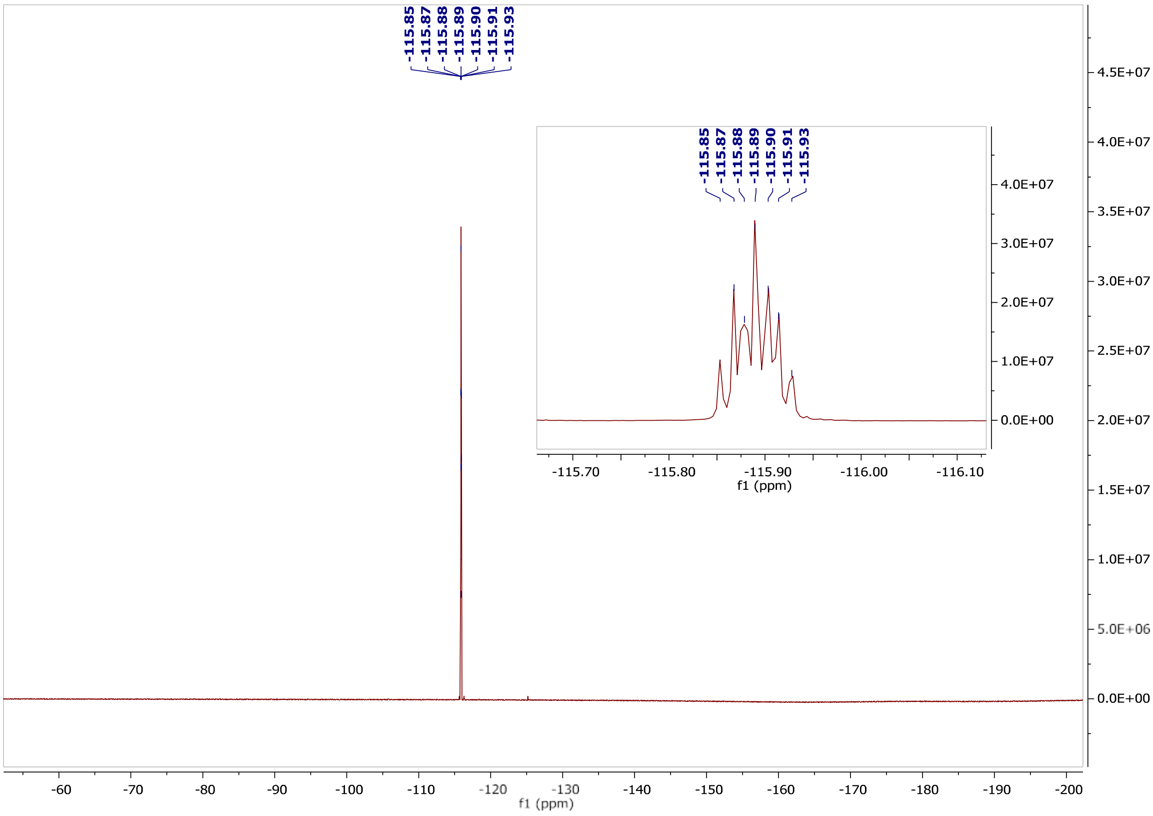

Figure S102. *tert*-Butyl 3-[4-(4-fluorophenyl)-1*H*-pyrazol-1-yl]-3-(2-methoxy-2-oxoethyl)azetidine-1- carboxylate (5g) ^{19}F NMR spectrum (376 MHz, $CDCl_3$).

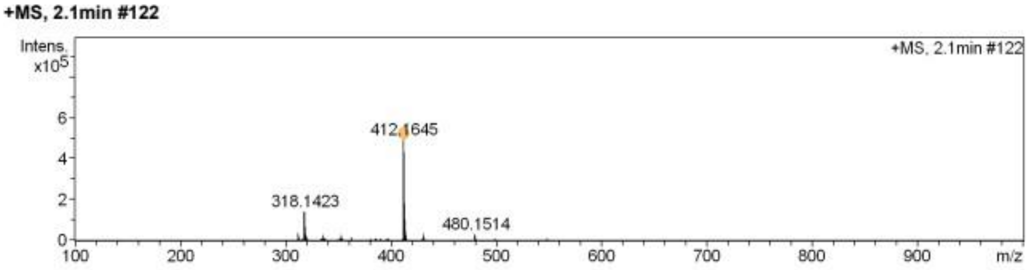

Meas. m/z	#	Ion Formula	m/z	err [ppm]	mSigma	# Sigma	Score	rdb	e⁻ Conf	N-Rule
412.1645	1	C20H24FN3NaO4	412.1643	0.5	14.7	2	100.00	9.5	even	ok

Figure S103. *tert*-Butyl 3-[4-(4-fluorophenyl)-1*H*-pyrazol-1-yl]-3-(2-methoxy-2-oxoethyl)azetidine-1- carboxylate (5g) HRMS (ESI-TOF).

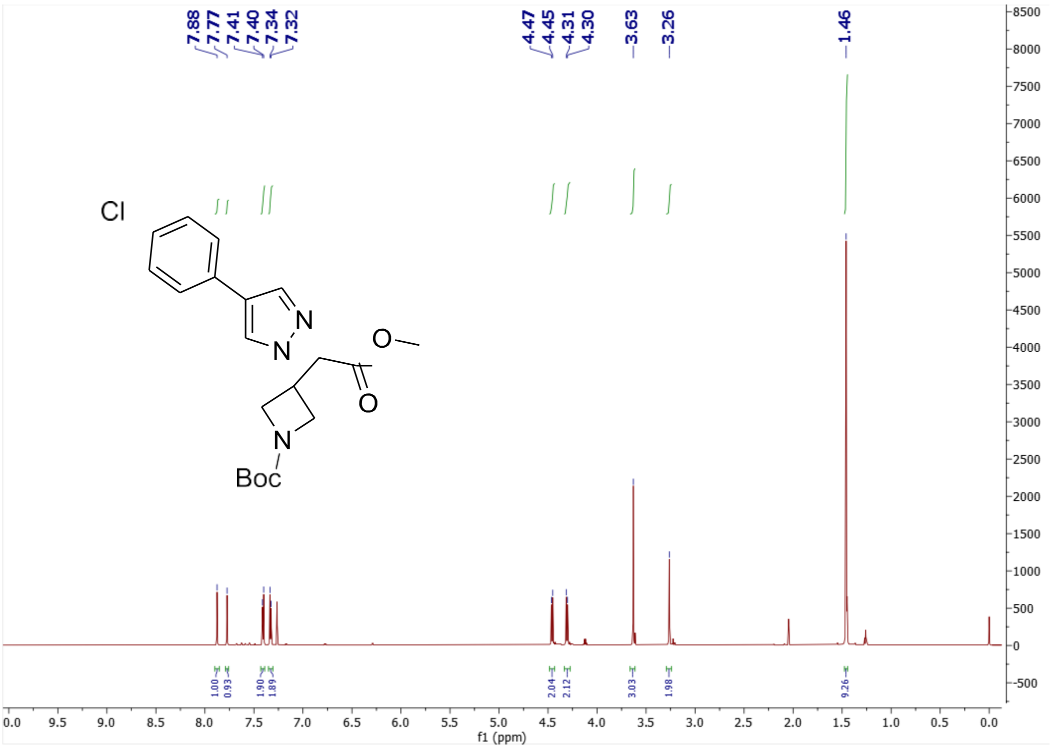

Figure S104. *tert*-Butyl 3-[4-(4-chlorophenyl)-1*H*-pyrazol-1-yl]-3-(2-methoxy-2-oxoethyl)azetidine-1- carboxylate (5h) ^{1}H NMR spectrum (700 MHz, $CDCl_3$).

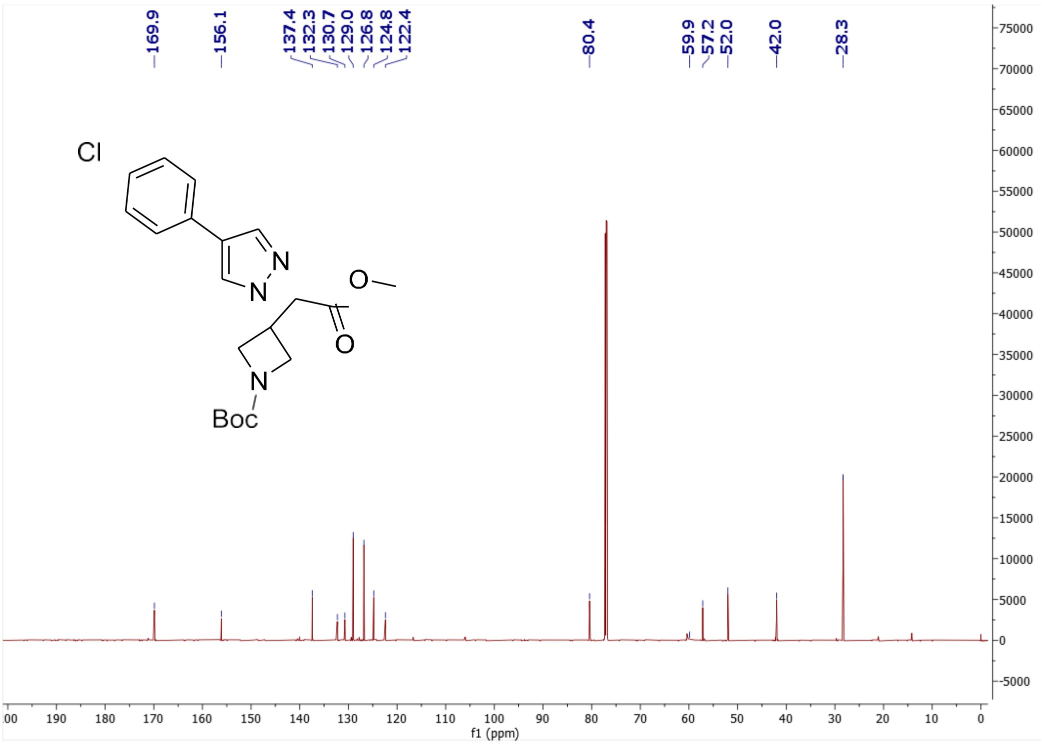

Figure S105. *tert*-Butyl 3-[4-(4-chlorophenyl)-1*H*-pyrazol-1-yl]-3-(2-methoxy-2-oxoethyl)azetidine-1- carboxylate (5h) ^{13}C NMR spectrum (176 MHz, $CDCl_3$).

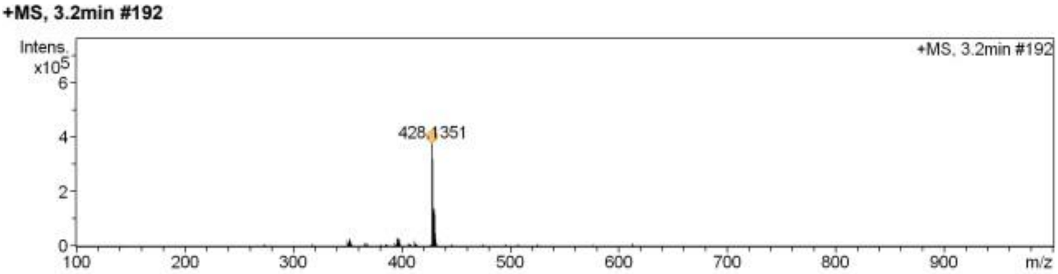

Meas. m/z	#	Ion Formula	m/z	err [ppm]	mSigma	# Sigma	Score	rdb	e⁻ Conf	N-Rule
428.1351	1	C20H24ClN3NaO4	428.1348	-0.8	11.1	1	100.00	9.5	even	ok

Figure S106. *tert*-Butyl 3-[4-(4-chlorophenyl)-1*H*-pyrazol-1-yl]-3-(2-methoxy-2-oxoethyl)azetidine-1- carboxylate (5h) HRMS (ESI-TOF).

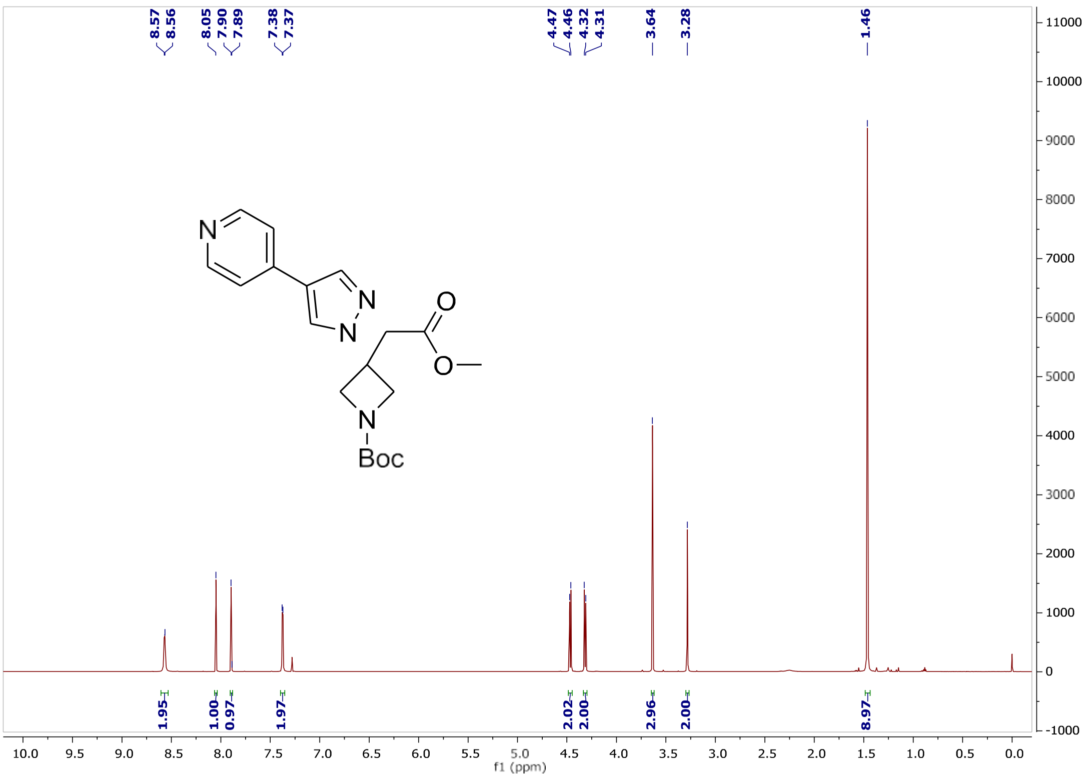

Figure S107. *tert*-Butyl 3-(2-methoxy-2-oxoethyl)-3-[4-(pyridin-4-yl)-1*H*-pyrazol-1-yl]azetidine-1-carboxylate (5i) ^{1}H NMR spectrum (700 MHz, $CDCl_3$).

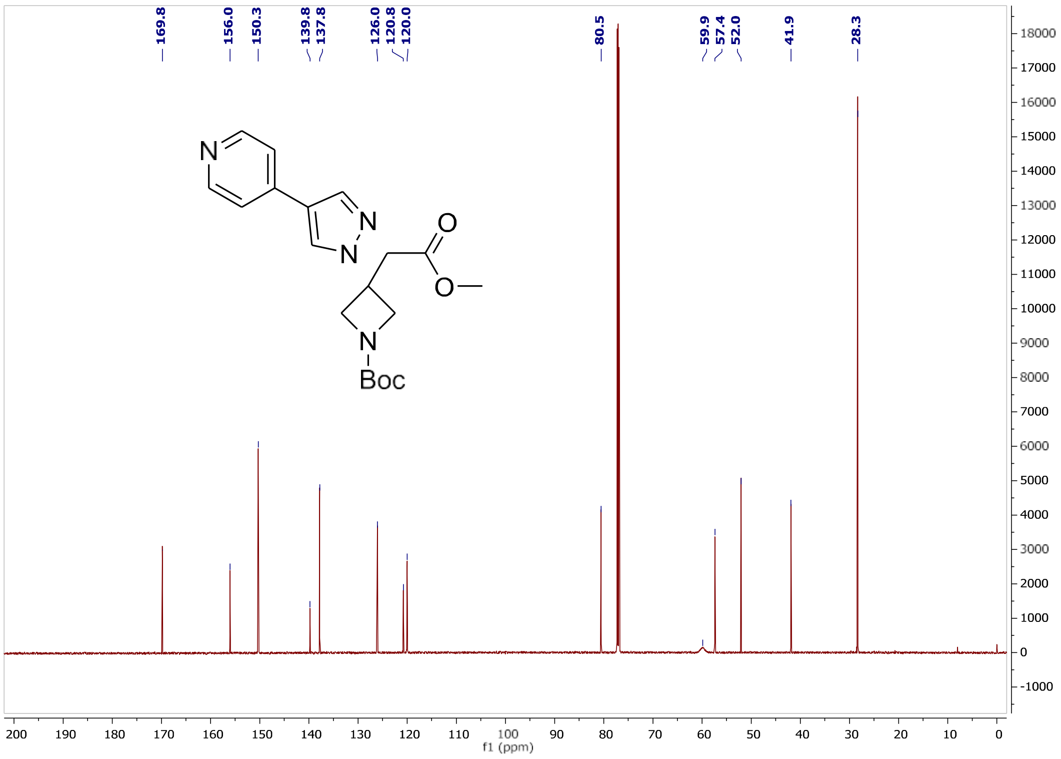

Figure S108. *tert*-Butyl 3-(2-methoxy-2-oxoethyl)-3-[4-(pyridin-4-yl)-1*H*-pyrazol-1-yl]azetidine-1-carboxylate (5i) ^{13}C NMR spectrum (176 MHz, $CDCl_3$).

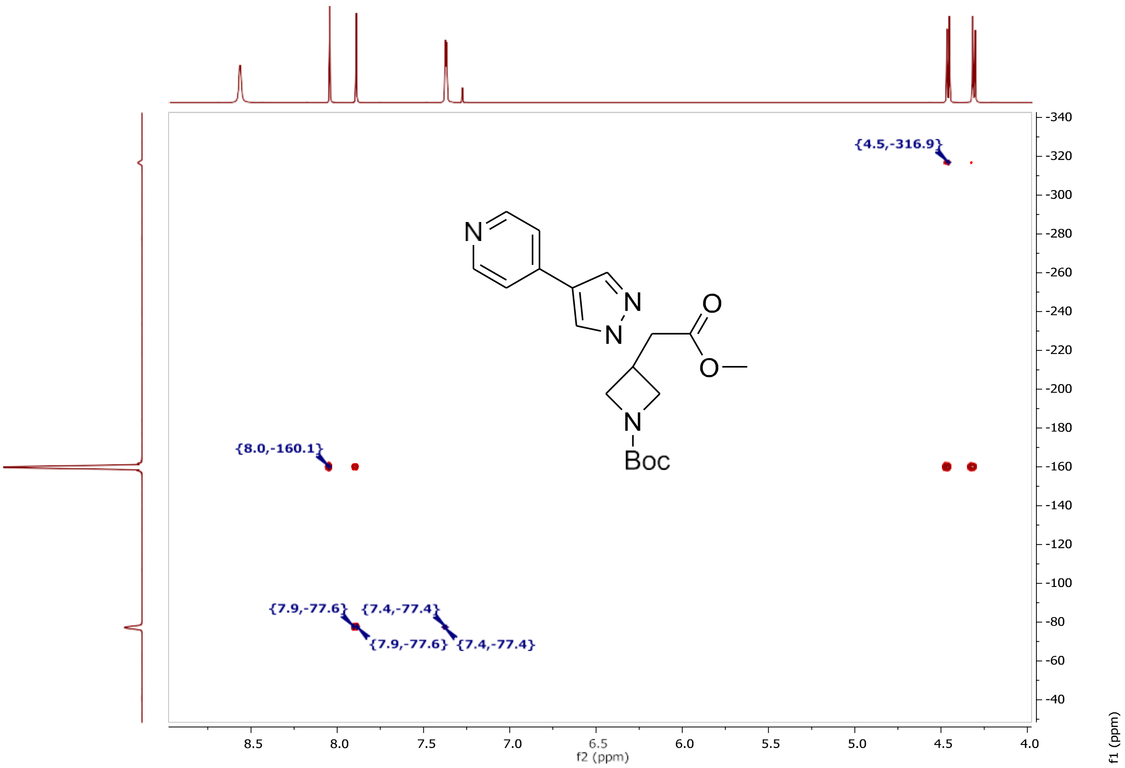

Figure S109. *tert*-Butyl 3-(2-methoxy-2-oxoethyl)-3-[4-(pyridin-4-yl)-1*H*-pyrazol-1-yl]azetidine-1-carboxylate (5i) ^{1}H-^{15}N HMBC spectrum (71 MHz, $CDCl_3$).

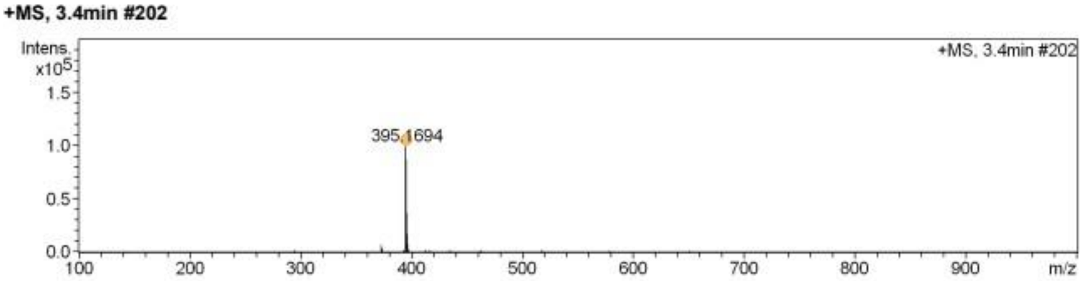

Meas. m/z	#	Ion Formula	m/z	err [ppm]	mSigma	# Sigma	Score	rdb	e⁻ Conf	N-Rule
395.1694	1	C19H24N4NaO4	395.1690	-1.0	13.1	1	100.00	9.5	even	ok

Figure S110. *tert*-Butyl 3-(2-methoxy-2-oxoethyl)-3-[4-(pyridin-4-yl)-1*H*-pyrazol-1-yl]azetidine-1-carboxylate (5i) HRMS (ESI-TOF).

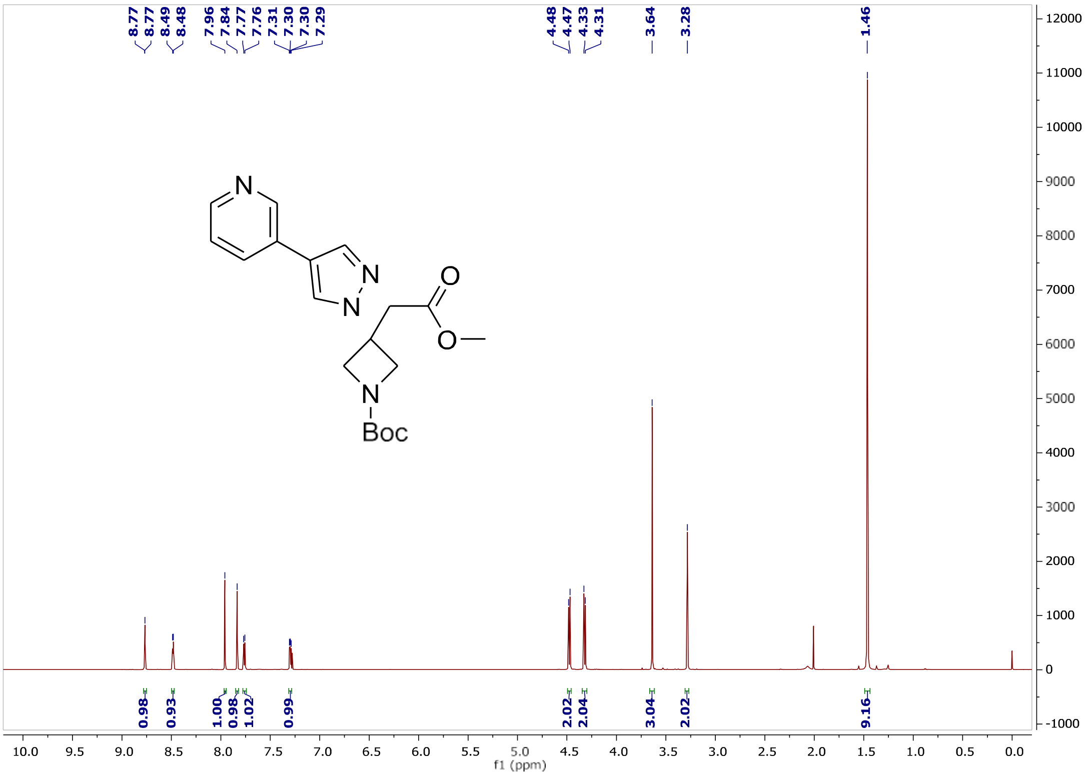

Figure S111. *tert*-Butyl 3-(2-methoxy-2-oxoethyl)-3-[4-(pyridin-3-yl)-1*H*-pyrazol-1-yl]azetidine-1-carboxylate (5j) ^{1}H NMR spectrum (700 MHz, $CDCl_3$).

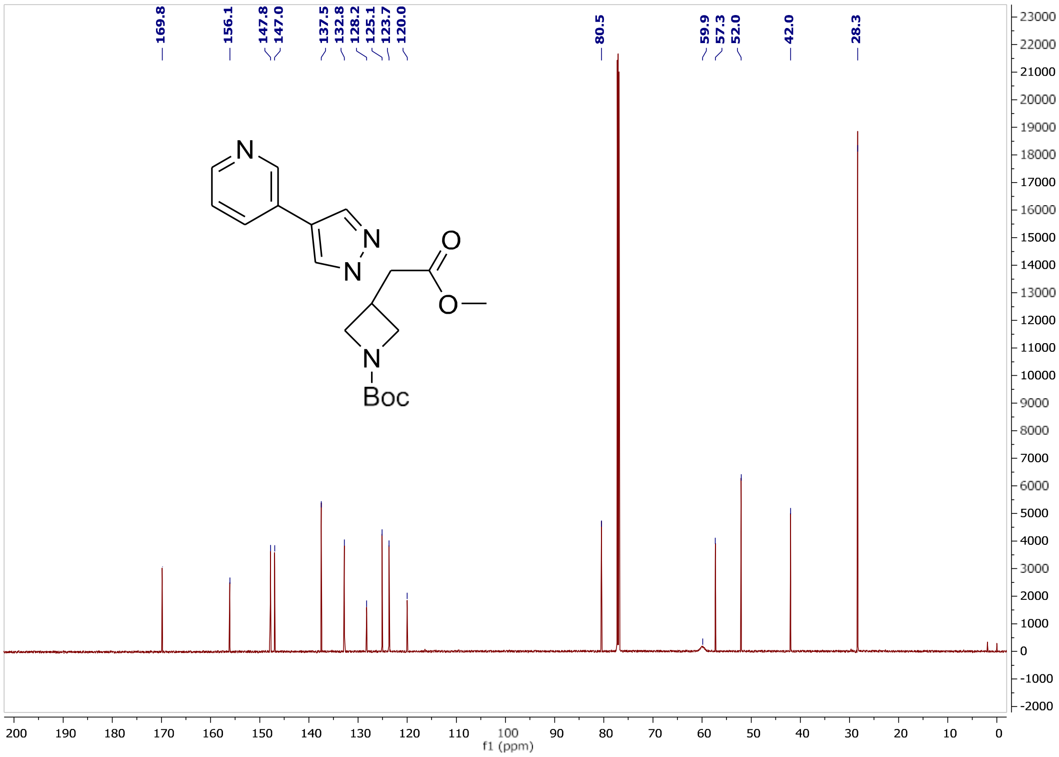

Figure S112. *tert*-Butyl 3-(2-methoxy-2-oxoethyl)-3-[4-(pyridin-3-yl)-1*H*-pyrazol-1-yl]azetidine-1-carboxylate (5j) ^{13}C NMR spectrum (176 MHz, $CDCl_3$).

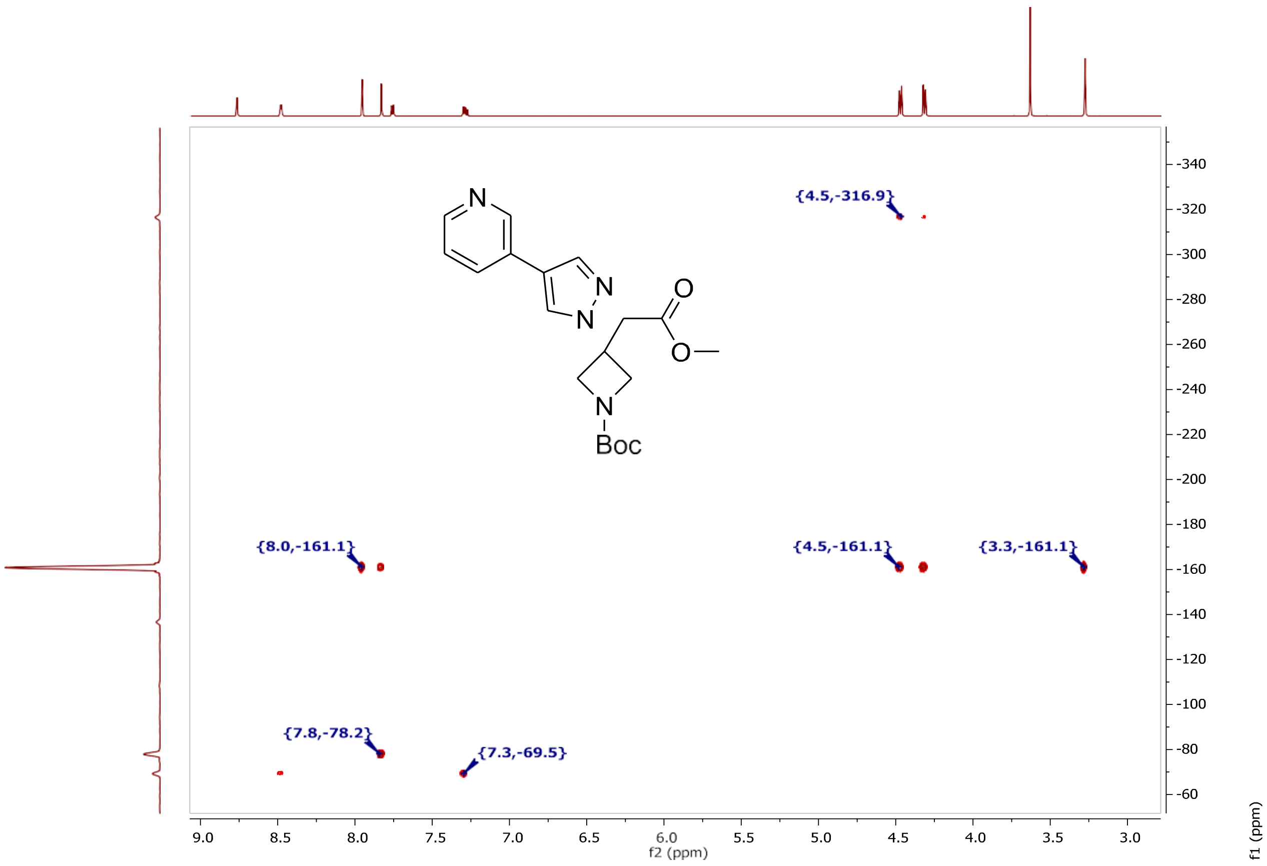

Figure S113. *tert*-Butyl 3-(2-methoxy-2-oxoethyl)-3-[4-(pyridin-3-yl)-1*H*-pyrazol-1-yl]azetidine-1-carboxylate (5j) ^{1}H-^{15}N HMBC spectrum (71 MHz, $CDCl_3$).

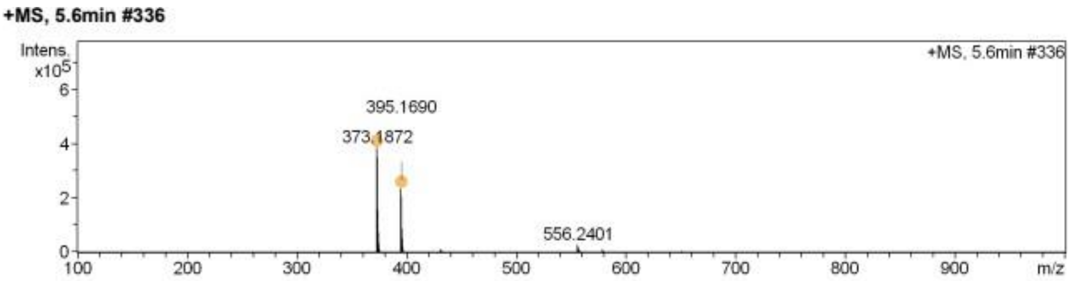

Meas. m/z	#	Ion Formula	m/z	err [ppm]	mSigma	# Sigma	Score	rdb	e⁻ Conf	N-Rule
373.1872	1	C19H25N4O4	373.1870	0.4	7.1	1	100.00	9.5	even	ok
395.1690	1	C19H24N4NaO4	395.1690	-0.1	9.0	1	100.00	9.5	even	ok

Figure S114. *tert*-Butyl 3-(2-methoxy-2-oxoethyl)-3-[4-(pyridin-3-yl)-1*H*-pyrazol-1-yl]azetidine-1-carboxylate (5j) HRMS (ESI-TOF).

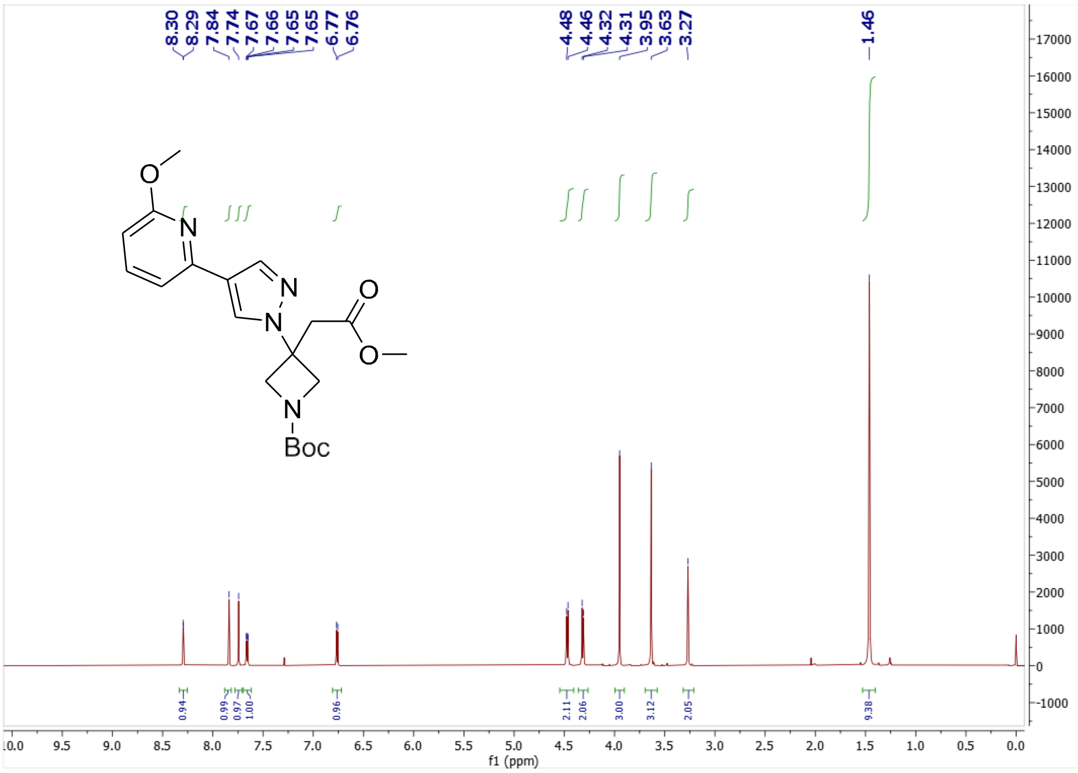

Figure S115. *tert*-Butyl 3-(2-methoxy-2-oxoethyl)-3-[4-(6-methoxypyridin-2-yl)-1*H*-pyrazol-1-yl]azetidine-1-carboxylate (5k) ^{1}H NMR spectrum (700 MHz, $CDCl_3$).

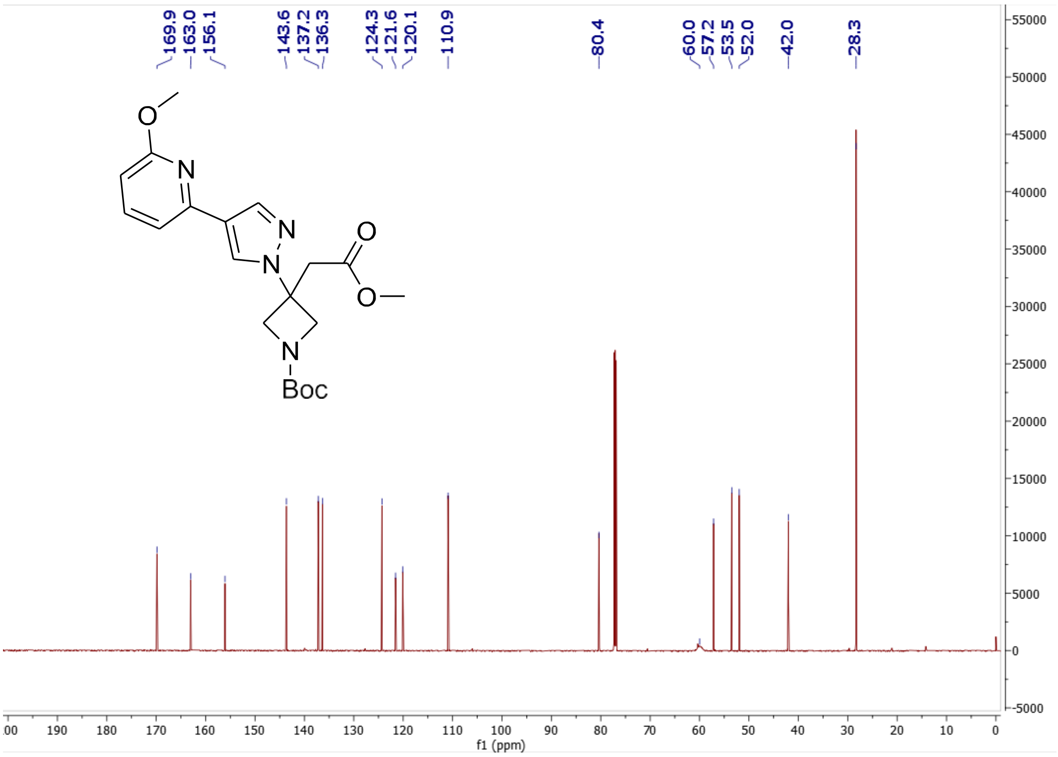

Figure S116. *tert*-Butyl 3-(2-methoxy-2-oxoethyl)-3-[4-(6-methoxypyridin-2-yl)-1*H*-pyrazol-1-yl]azetidine-1-carboxylate (5k) ^{13}C NMR spectrum (176 MHz, $CDCl_3$).

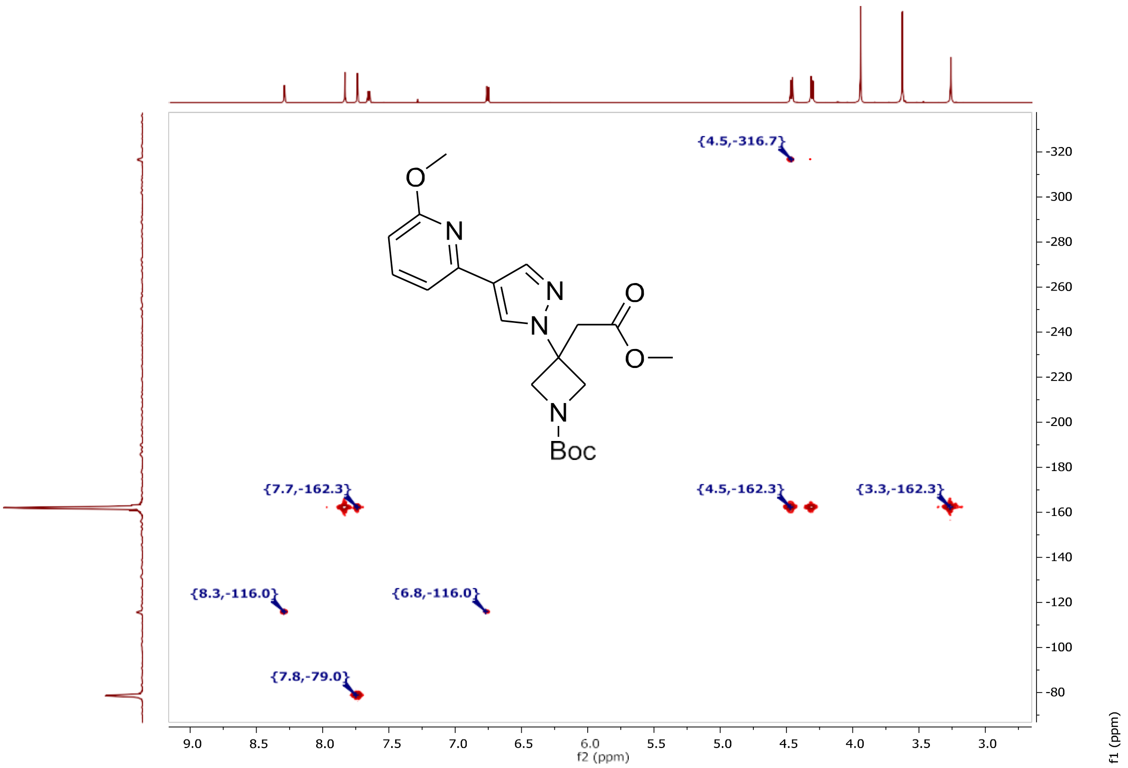

Figure S117. *tert*-Butyl 3-(2-methoxy-2-oxoethyl)-3-[4-(6-methoxypyridin-2-yl)-1*H*-pyrazol-1-yl]azetidine-1-carboxylate (5k) ^{1}H-^{15}N HMBC spectrum (71 MHz, $CDCl_3$).

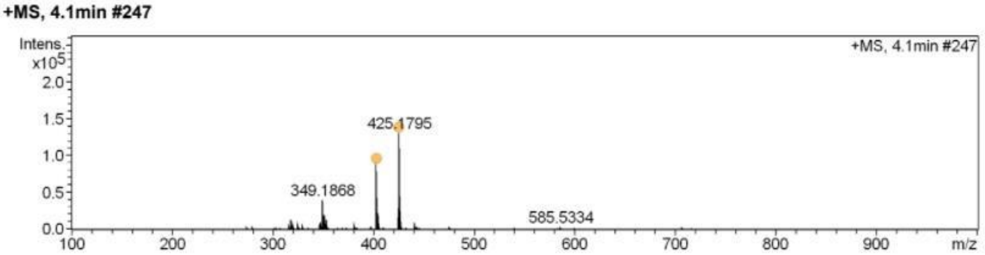

Meas. m/z	#	Ion Formula	m/z	err [ppm]	mSigma	# Sigma	Score	rdb	e⁻ Conf	N-Rule
403.1969	1	C20H27N4O5	403.1976	-1.8	10.3	1	100.00	9.5	even	ok
425.1795	1	C20H26N4NaO5	425.1795	0.0	2.4	1	100.00	9.5	even	ok

Figure S118. *tert*-Butyl 3-(2-methoxy-2-oxoethyl)-3-[4-(6-methoxypyridin-2-yl)-1*H*-pyrazol-1-yl]azetidine-1-carboxylate (5k) HRMS (ESI-TOF).

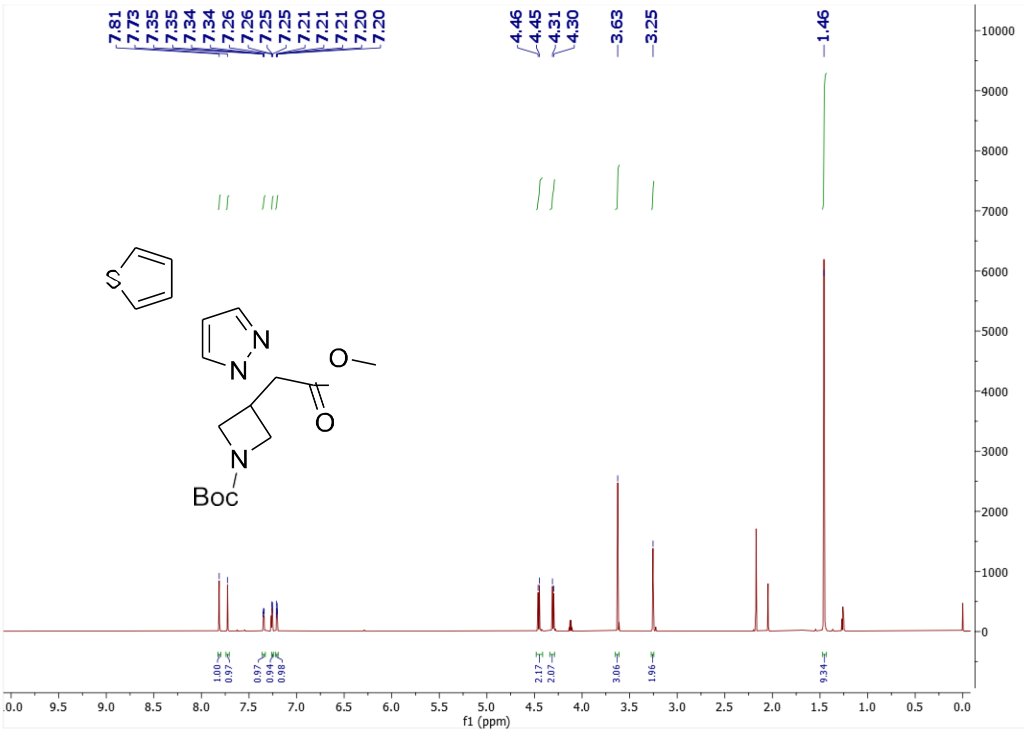

Figure S119. *tert*-Butyl 3-(2-methoxy-2-oxoethyl)-3-[4-(thiophen-3-yl)-1*H*-pyrazol-1-yl]azetidine-1-carboxylate (5l) ^{1}H NMR spectrum (700 MHz, $CDCl_3$).

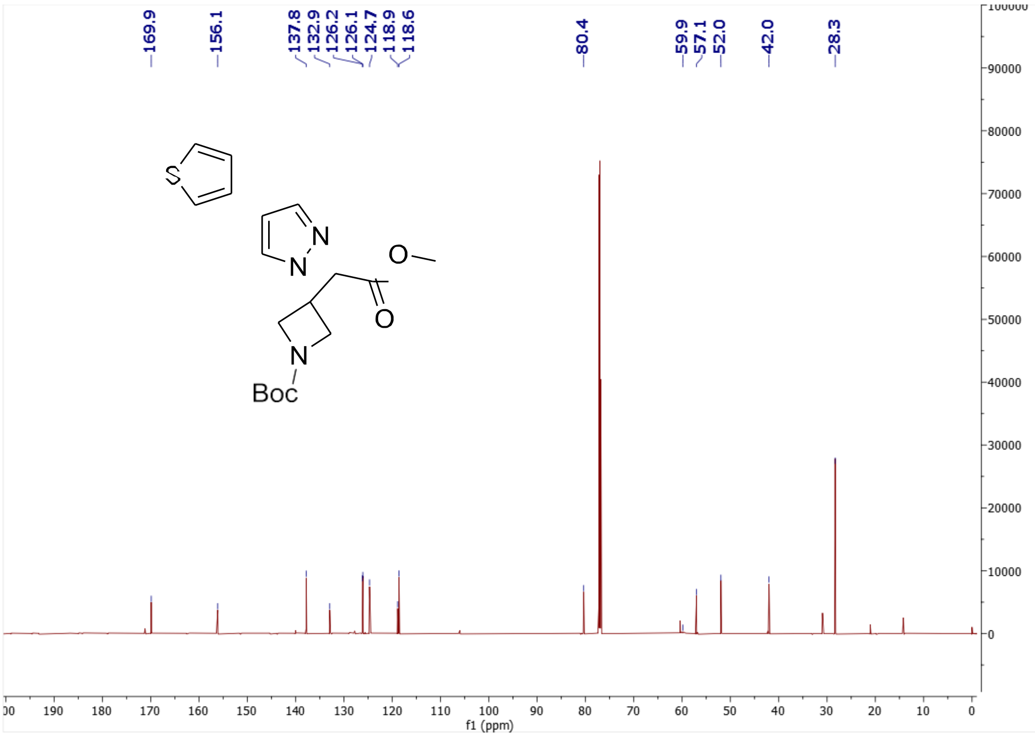

Figure S120. *tert*-Butyl 3-(2-methoxy-2-oxoethyl)-3-[4-(thiophen-3-yl)-1*H*-pyrazol-1-yl]azetidine-1-carboxylate (5l) ^{13}C NMR spectrum (176 MHz, $CDCl_3$).

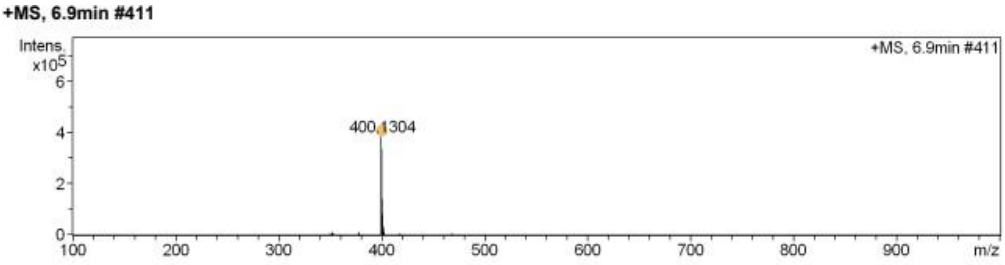

Meas. m/z	#	Ion Formula	m/z	err [ppm]	mSigma	# Sigma	Score	rdb	e⁻ Conf	N-Rule
400.1304	1	C18H23N3NaO4S	400.1301	-0.7	15.8	1	100.00	8.5	even	ok

Figure S121. *tert*-Butyl 3-(2-methoxy-2-oxoethyl)-3-[4-(thiophen-3-yl)-1*H*-pyrazol-1-yl]azetidine-1-carboxylate (5l) HRMS (ESI-TOF).

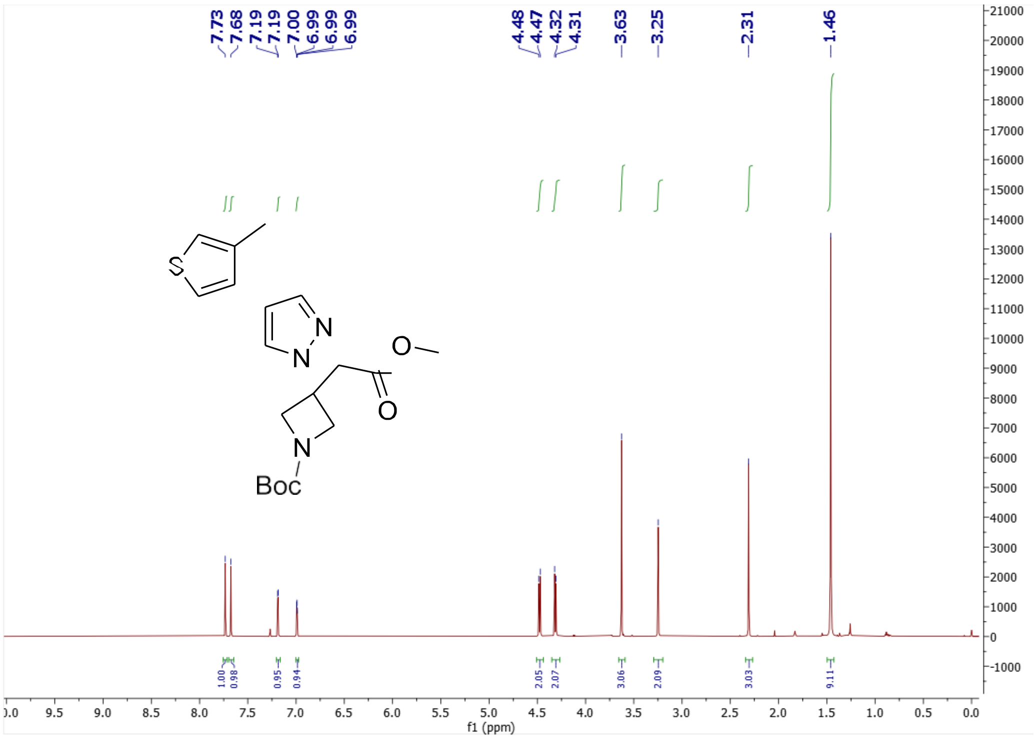

Figure S122. *tert*-Butyl 3-(2-methoxy-2-oxoethyl)-3-[4-(4-methylthiophen-3-yl)-1*H*-pyrazol-1-yl]azetidine-1-carboxylate (5m) ^{1}H NMR spectrum (700 MHz, $CDCl_3$).

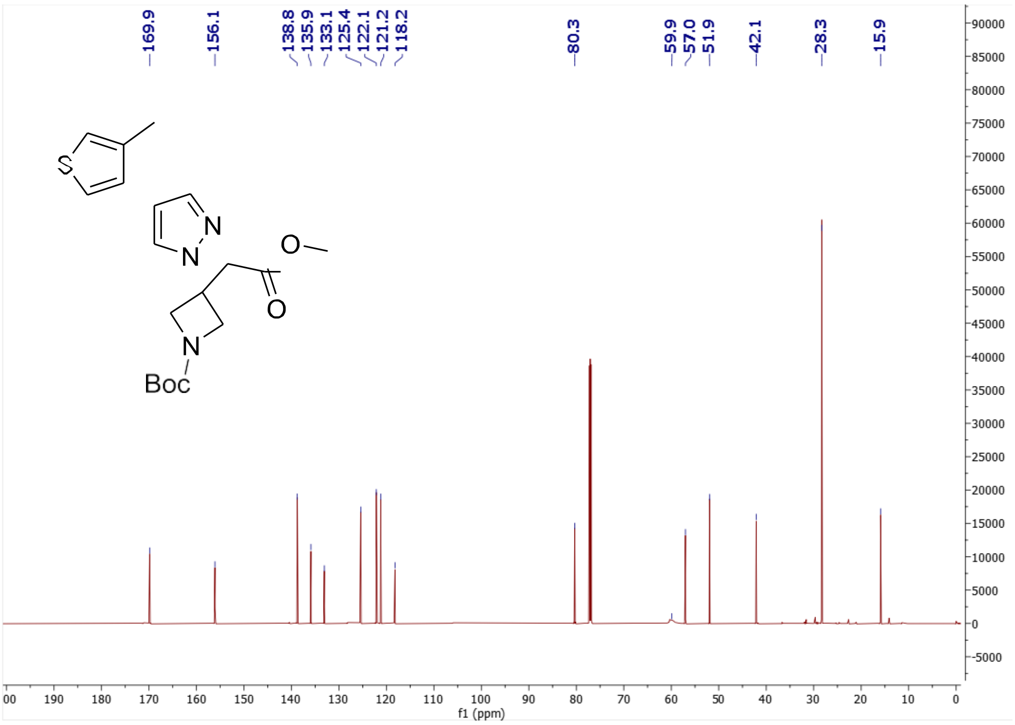

Figure S123. *tert*-Butyl 3-(2-methoxy-2-oxoethyl)-3-[4-(4-methylthiophen-3-yl)-1*H*-pyrazol-1-yl]azetidine-1-carboxylate (5m) ^{13}C NMR spectrum (176 MHz, $CDCl_3$).

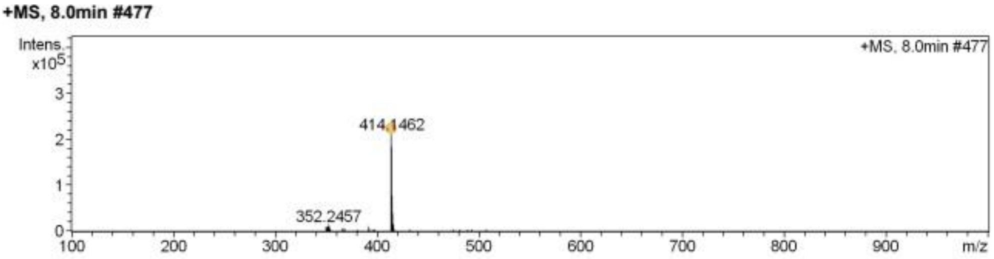

Meas. m/z	#	Ion Formula	m/z	err [ppm]	mSigma	# Sigma	Score	rdb	e⁻ Conf	N-Rule
414.1462	1	C19H25N3NaO4S	414.1458	-1.1	4.3	1	100.00	8.5	even	ok

Figure S124. *tert*-Butyl 3-(2-methoxy-2-oxoethyl)-3-[4-(4-methylthiophen-3-yl)-1*H*-pyrazol-1-yl]azetidine-1-carboxylate (5m) HRMS (ESI-TOF).

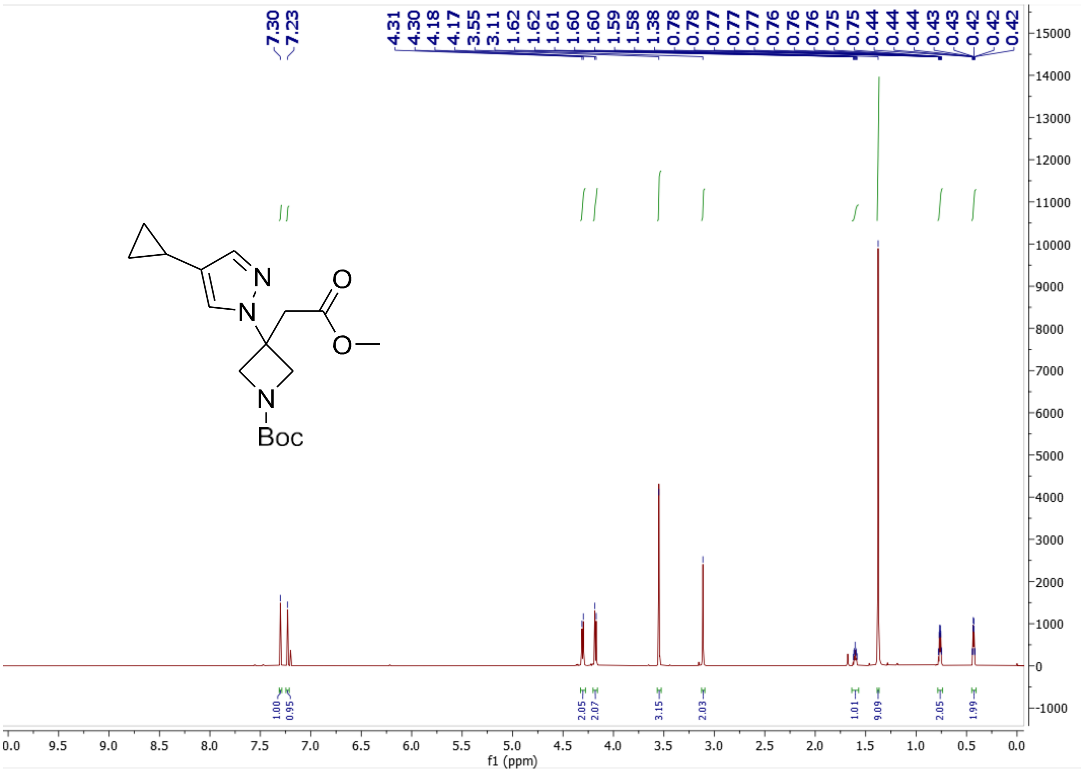

Figure S125. *tert*-butyl 3-(4-cyclopropyl-1*H*-pyrazol-1-yl)-3-(2-methoxy-2-oxoethyl)azetidine-1- carboxylate (5n) ^{1}H NMR spectrum (700 MHz, $CDCl_3$).

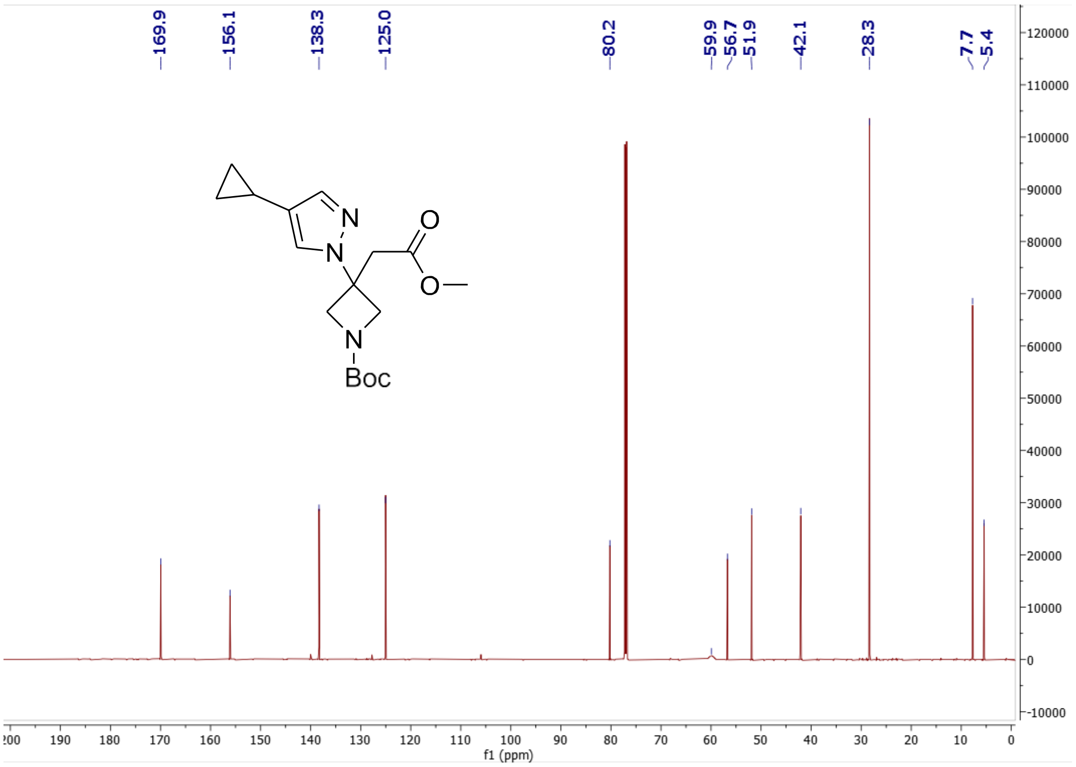

Figure S126. *tert*-butyl 3-(4-cyclopropyl-1*H*-pyrazol-1-yl)-3-(2-methoxy-2-oxoethyl)azetidine-1- carboxylate (5n) ^{13}C NMR spectrum (176 MHz, $CDCl_3$).

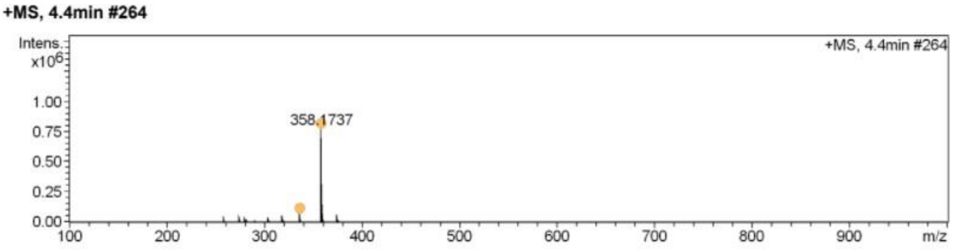

Meas. m/z	#	Ion Formula	m/z	err [ppm]	mSigma	# Sigma	Score	rdb	e⁻ Conf	N-Rule
336.1910	1	C17H26N3O4	336.1918	2.4	3.6	1	100.00	6.5	even	ok
358.1737	1	C17H25N3NaO4	358.1737	0.2	5.2	1	100.00	6.5	even	ok

Figure S127. *tert*-butyl 3-(4-cyclopropyl-1*H*-pyrazol-1-yl)-3-(2-methoxy-2-oxoethyl)azetidine-1- carboxylate (5n) HRMS (ESI-TOF).

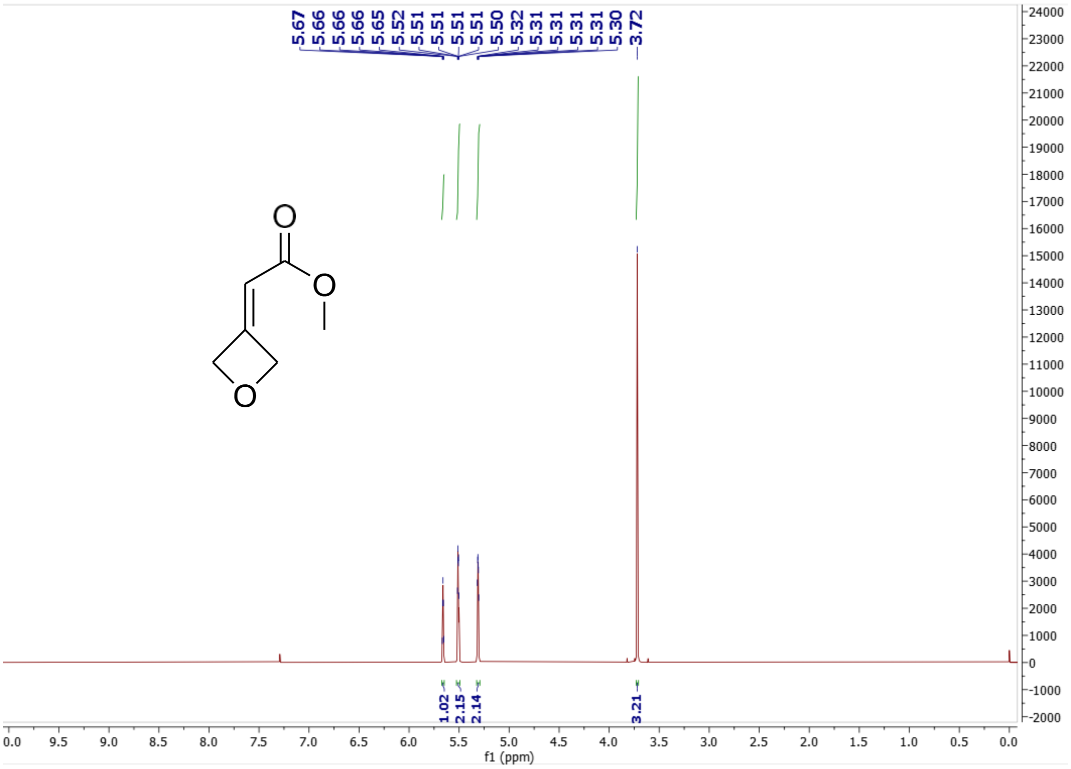

Figure S128. Methyl 2-(oxetan-3-ylidene)acetate (7) ^{1}H NMR spectrum (700 MHz, $CDCl_3$).

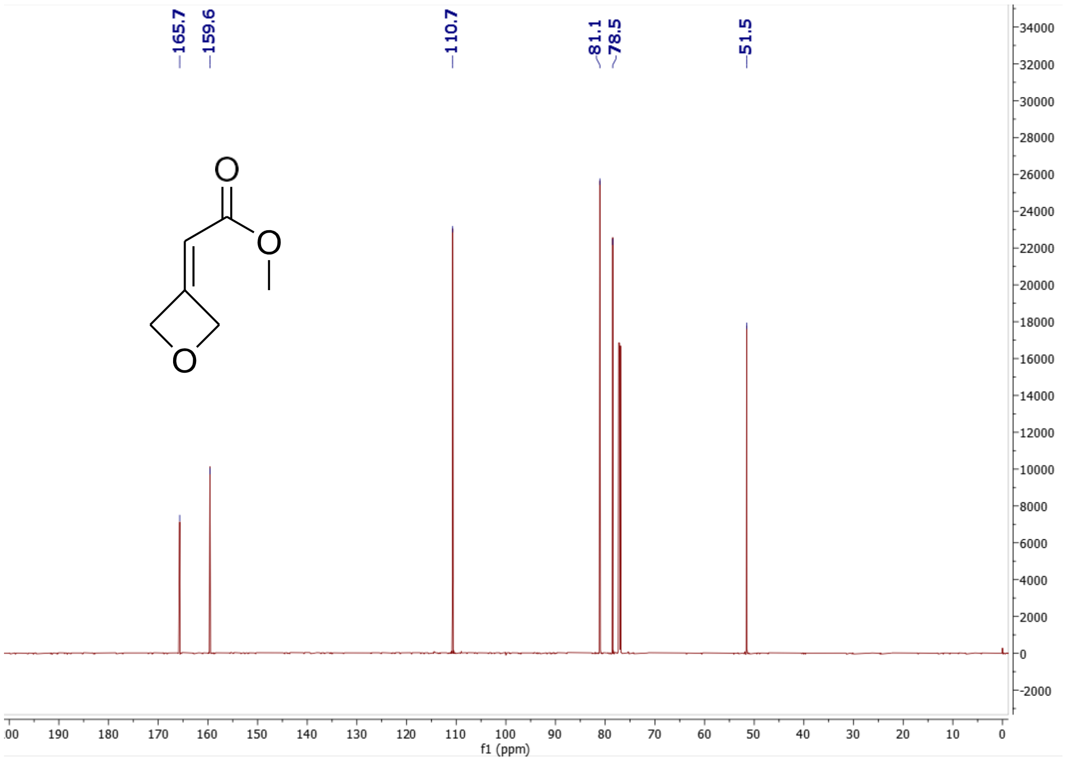

Figure S129. Methyl 2-(oxetan-3-ylidene)acetate (7) ^{13}C NMR spectrum (176 MHz, $CDCl_3$).

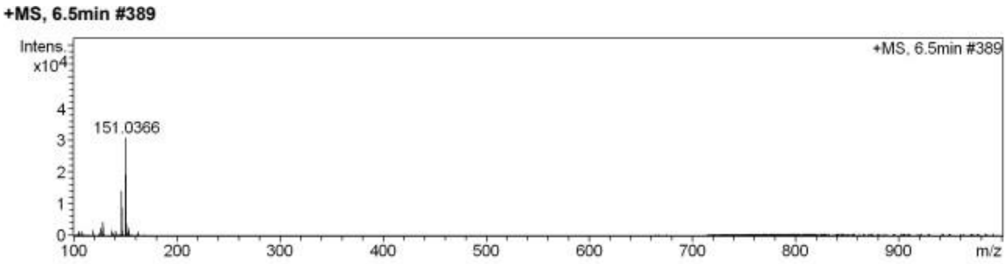

Meas. m/z	#	Ion Formula	m/z	err [ppm]	mSigma	# Sigma	Score	rdb	e⁻ Conf	N-Rule
151.0366	1	C6H8NaO3	151.0366	-0.1	4.1	1	100.00	2.5	even	ok

Figure S130. Methyl 2-(oxetan-3-ylidene)acetate (7) HRMS (ESI-TOF).

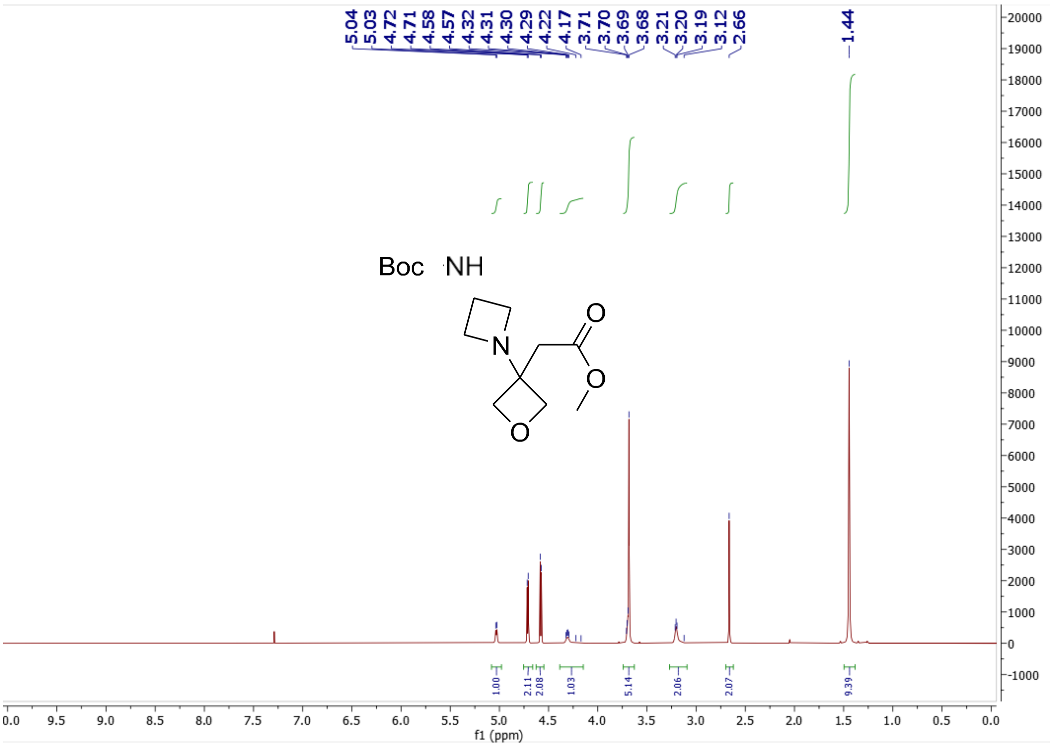

Figure S131. Methyl (3-{3-[(*tert*-butoxycarbonyl)amino]azetidin-1-yl}oxetan-3-yl)acetate (8a) ^{1}H NMR spectrum (700 MHz, $CDCl_3$).

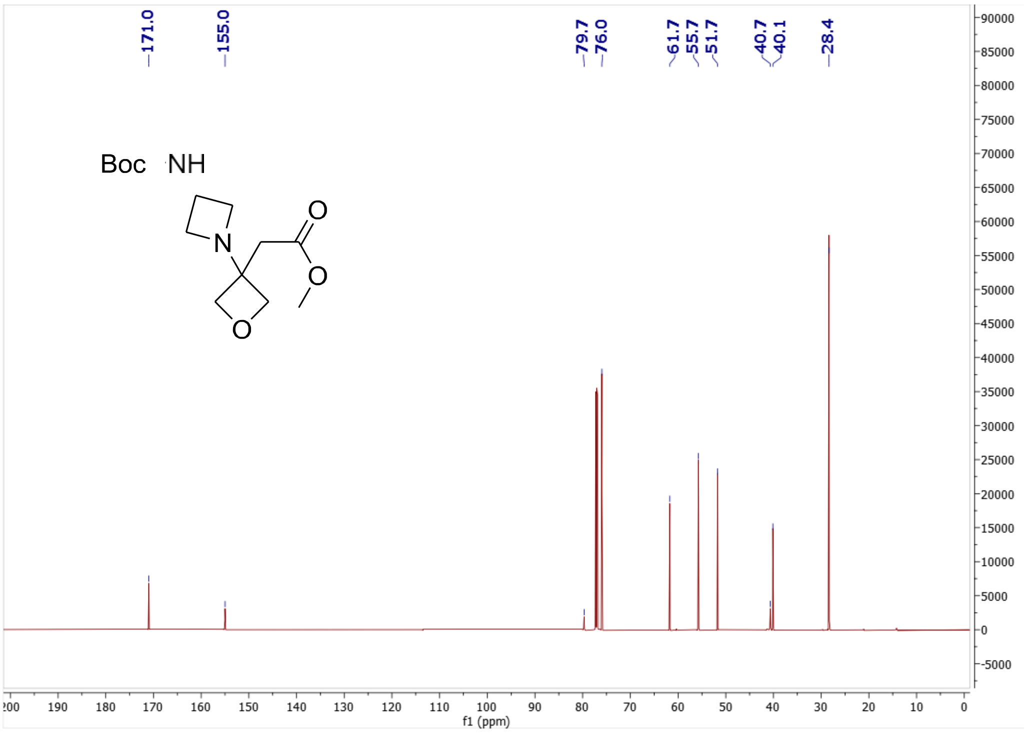

Figure S132. Methyl (3-{3-[(*tert*-butoxycarbonyl)amino]azetidin-1-yl}oxetan-3-yl)acetate (8a) ^{13}C NMR spectrum (176 MHz, $CDCl_3$).

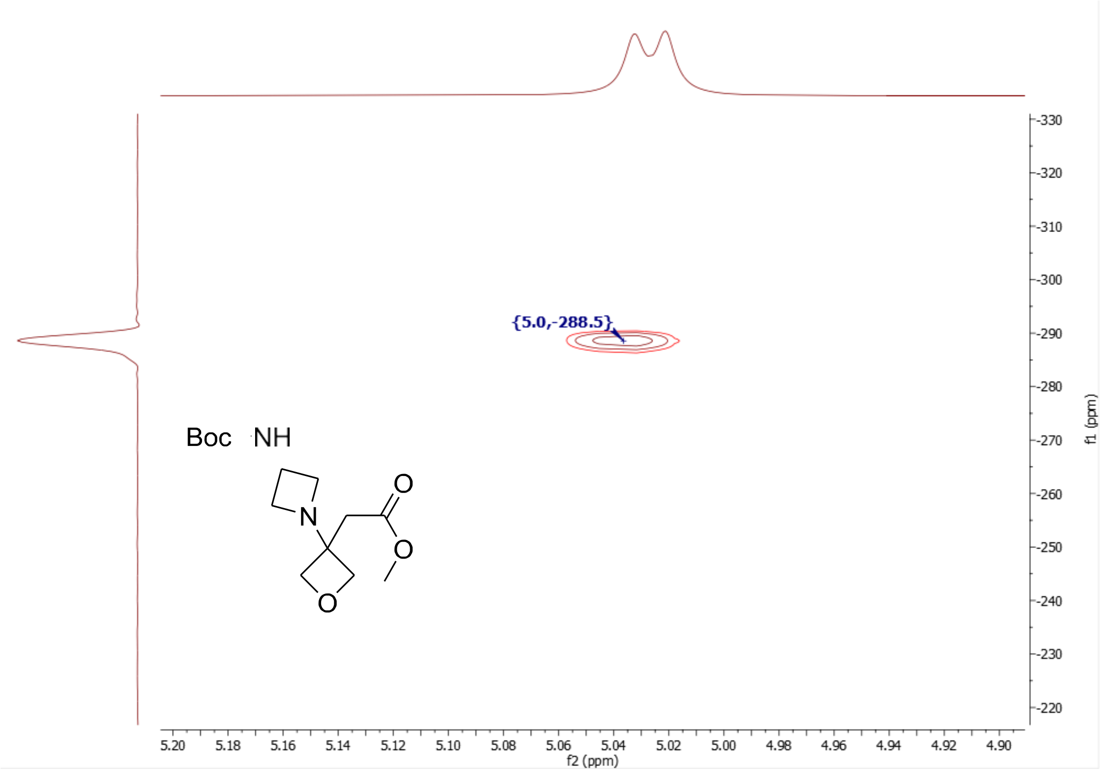

Figure S133. Methyl (3-{3-[(*tert*-butoxycarbonyl)amino]azetidin-1-yl}oxetan-3-yl)acetate (8a) ^{1}H-^{15}N HSQC spectrum (71 MHz, $CDCl_3$).

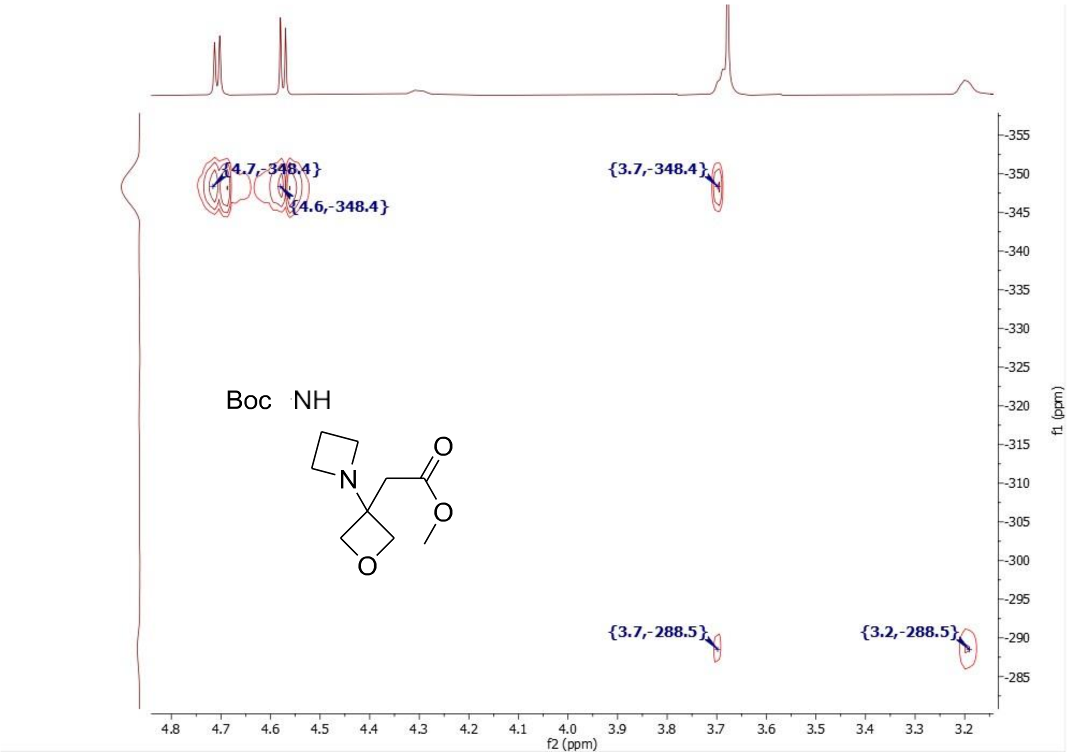

Figure S134. Methyl (3-{3-[(*tert*-butoxycarbonyl)amino]azetidin-1-yl}oxetan-3-yl)acetate (8a) ^{1}H-^{15}N HMBC spectrum (71 MHz, $CDCl_3$).

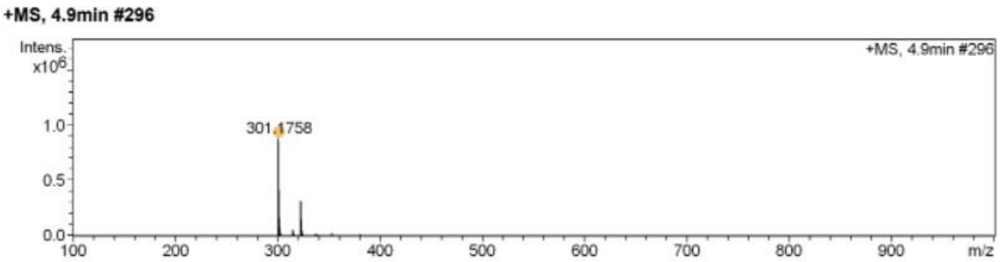

Meas. m/z	#	Ion Formula	m/z	err [ppm]	mSigma	# Sigma	Score	rdb	e⁻ Conf	N-Rule
301.1758	1	C14H25N2O5	301.1758	-0.1	14.4	2	100.00	3.5	even	ok

Figure S135. Methyl (3-{3-[(*tert*-butoxycarbonyl)amino]azetidin-1-yl}oxetan-3-yl)acetate (8a) HRMS (ESI-TOF).

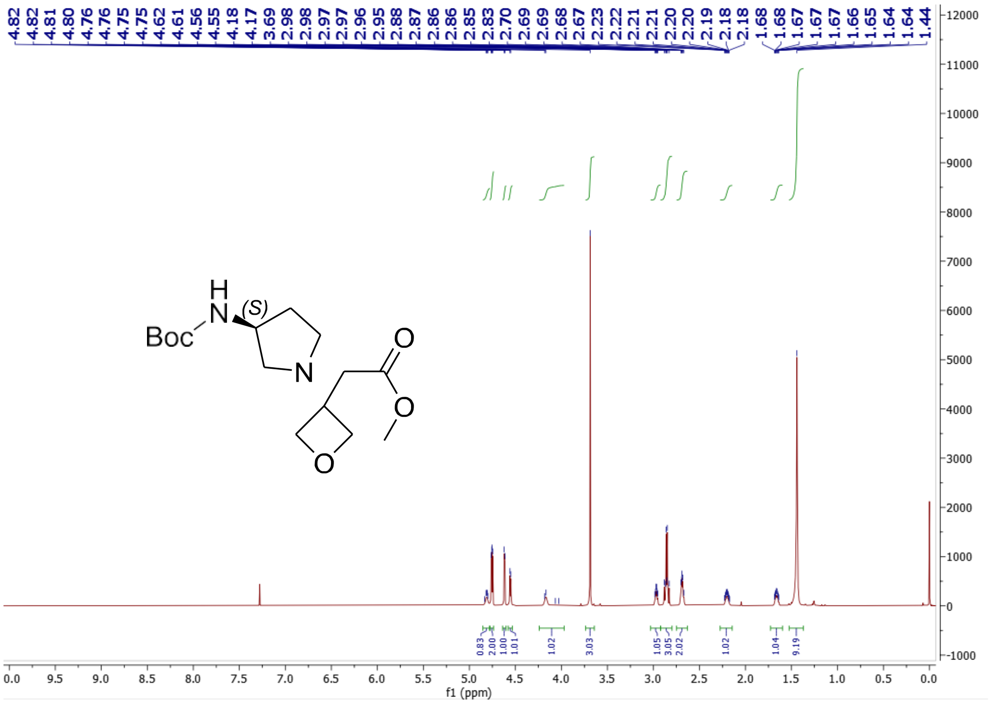

Figure S136. Methyl (3-{(3*S*)-3-[(*tert*-butoxycarbonyl)amino]pyrrolidin-1-yl}oxetan-3-yl)acetate (8b) ^{1}H NMR spectrum (700 MHz, $CDCl_3$).

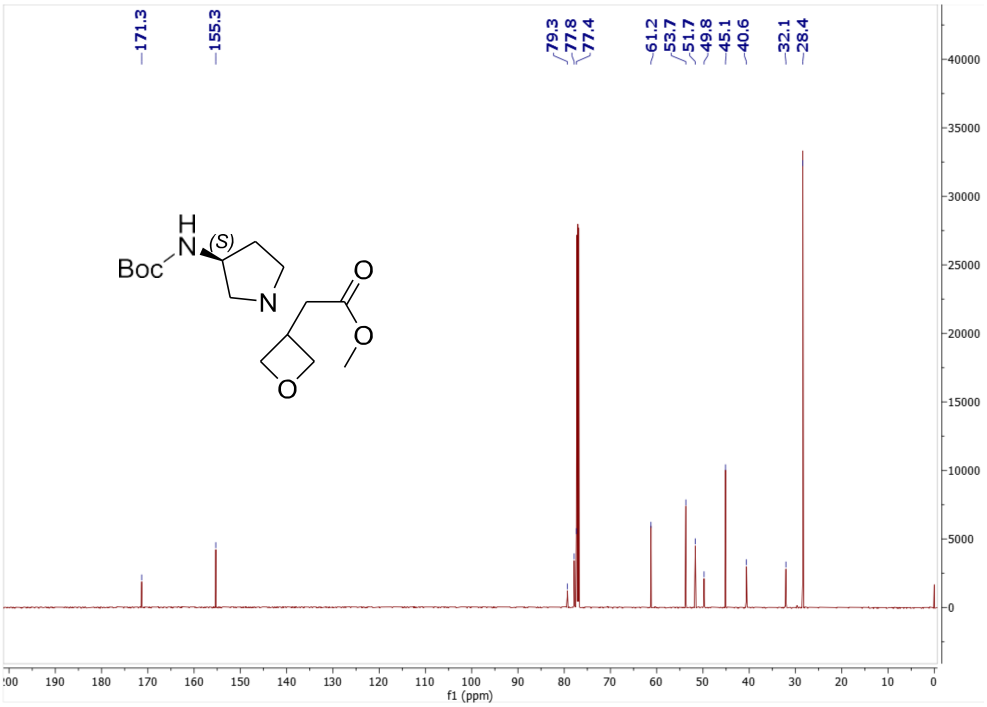

Figure S137. Methyl (3-{(3*S*)-3-[(*tert*-butoxycarbonyl)amino]pyrrolidin-1-yl}oxetan-3-yl)acetate (8b) ^{13}C NMR spectrum (176 MHz, $CDCl_3$).

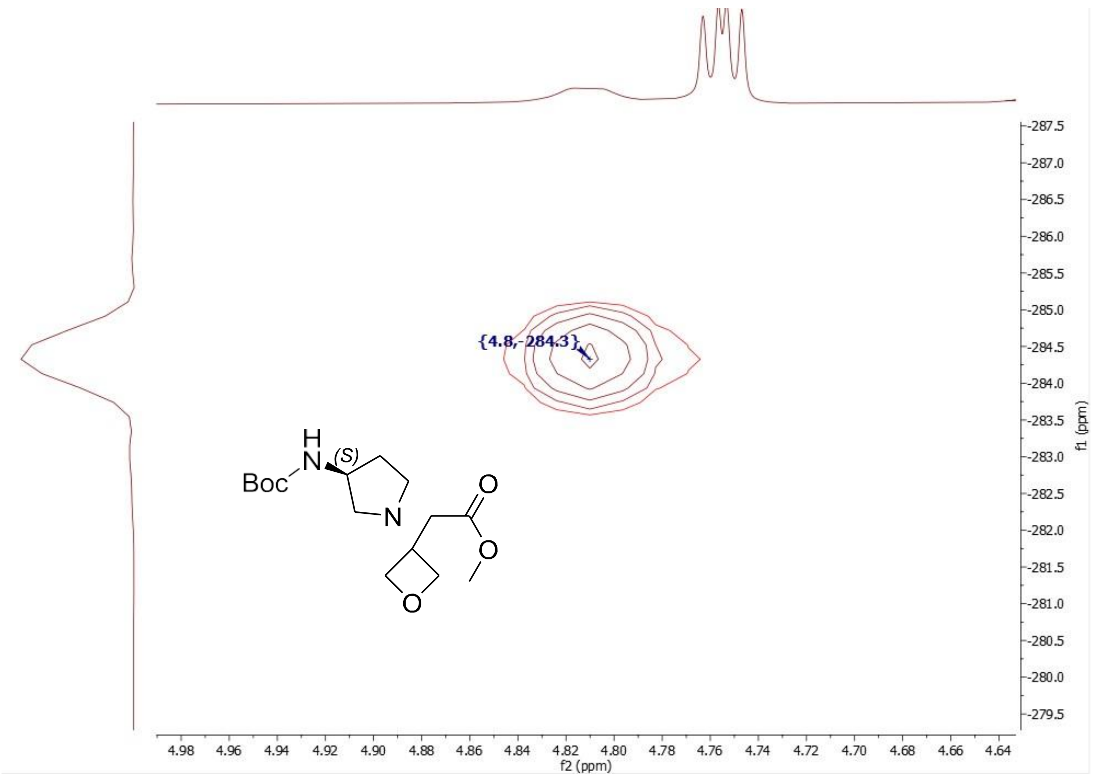

Figure S138. Methyl (3-{(3*S*)-3-[(*tert*-butoxycarbonyl)amino]pyrrolidin-1-yl}oxetan-3-yl)acetate (8b) ^{1}H-^{15}N HSQC spectrum (71 MHz, $CDCl_3$).

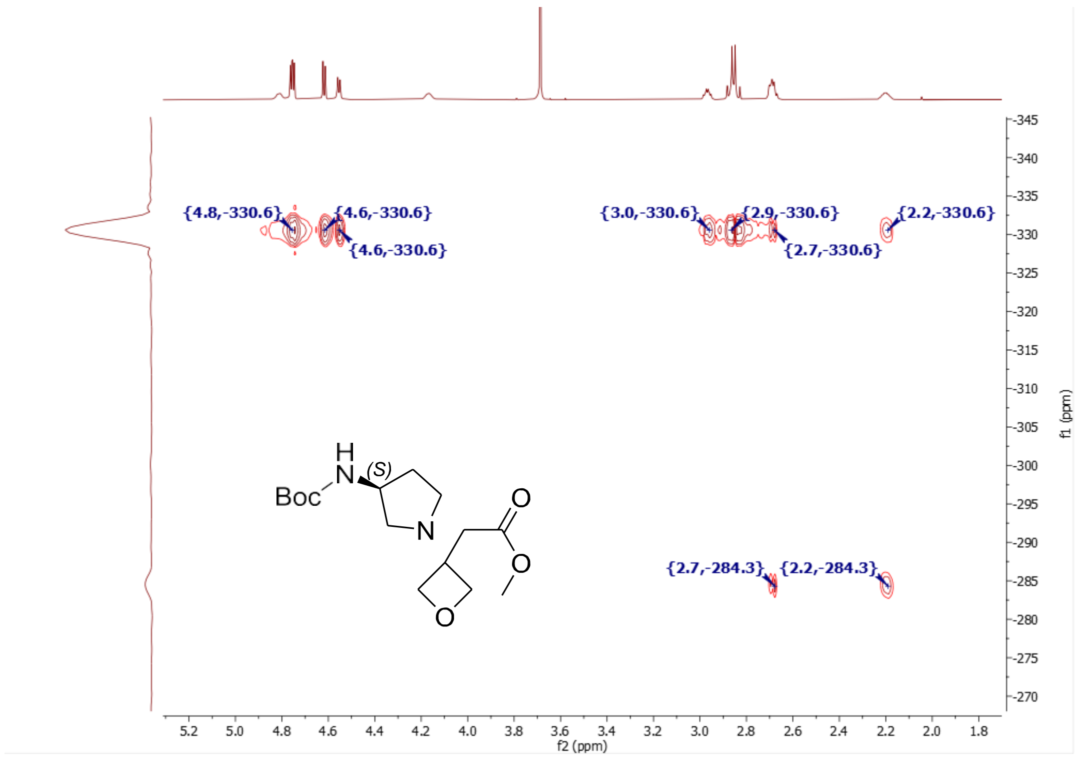

Figure S139. Methyl (3-{(3*S*)-3-[(*tert*-butoxycarbonyl)amino]pyrrolidin-1-yl}oxetan-3-yl)acetate (8b) ^{1}H-^{15}N HMBC spectrum (71 MHz, $CDCl_3$).

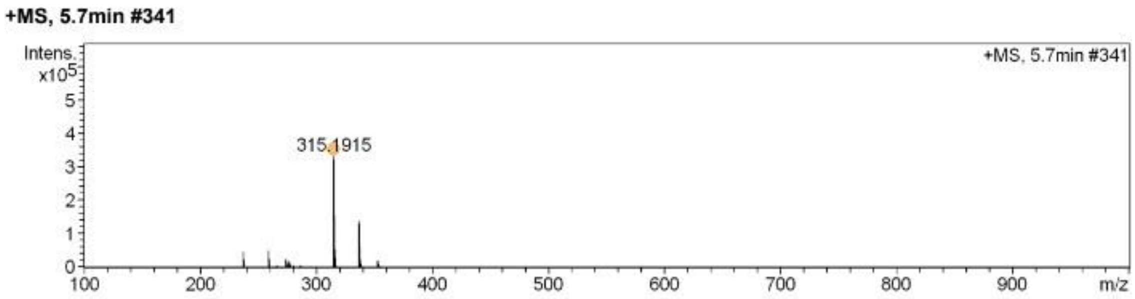

Meas. m/z	#	Ion Formula	m/z	err [ppm]	mSigma	# Sigma	Score	rdb	e⁻ Conf	N-Rule
315.1915	1	C15H27N2O5	315.1914	-0.1	0.6	1	100.00	3.5	even	ok

Figure S140. Methyl (3-{(3*S*)-3-[(*tert*-butoxycarbonyl)amino]pyrrolidin-1-yl}oxetan-3-yl)acetate (8b) HRMS (ESI-TOF).

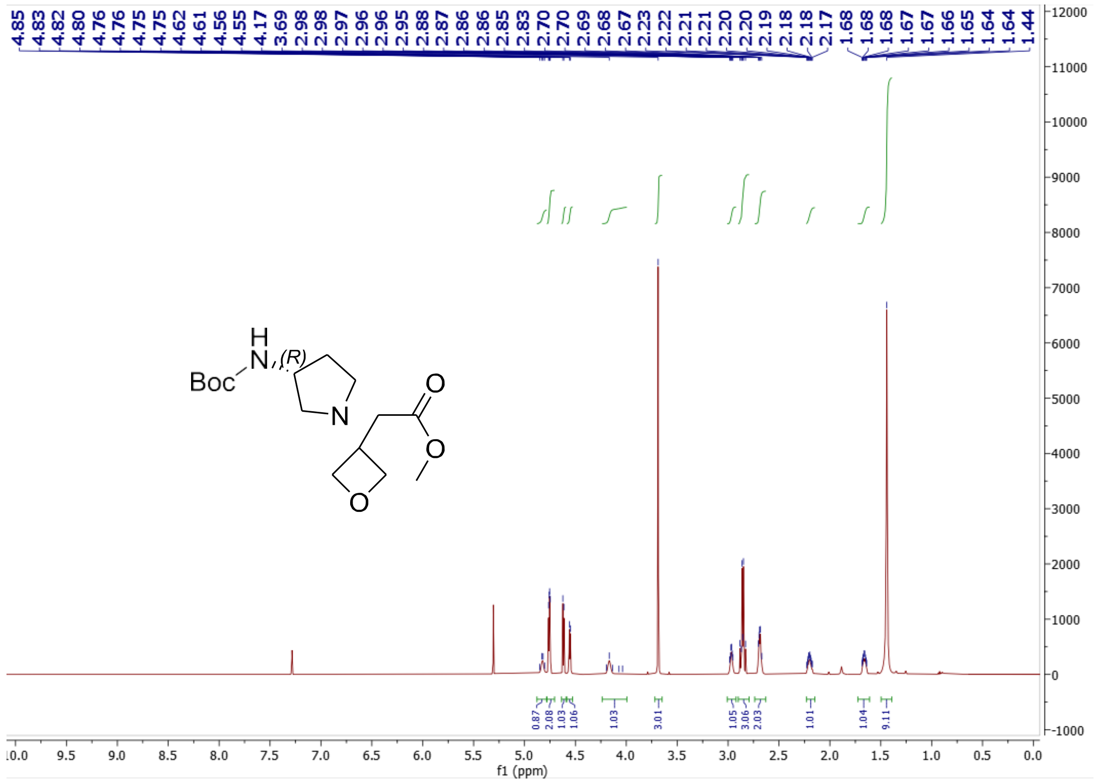

Figure S141. Methyl (3-{(3*R*)-3-[(*tert*-butoxycarbonyl)amino]pyrrolidin-1-yl}oxetan-3-yl)acetate (8c) ^{1}H NMR spectrum (700 MHz, $CDCl_3$).

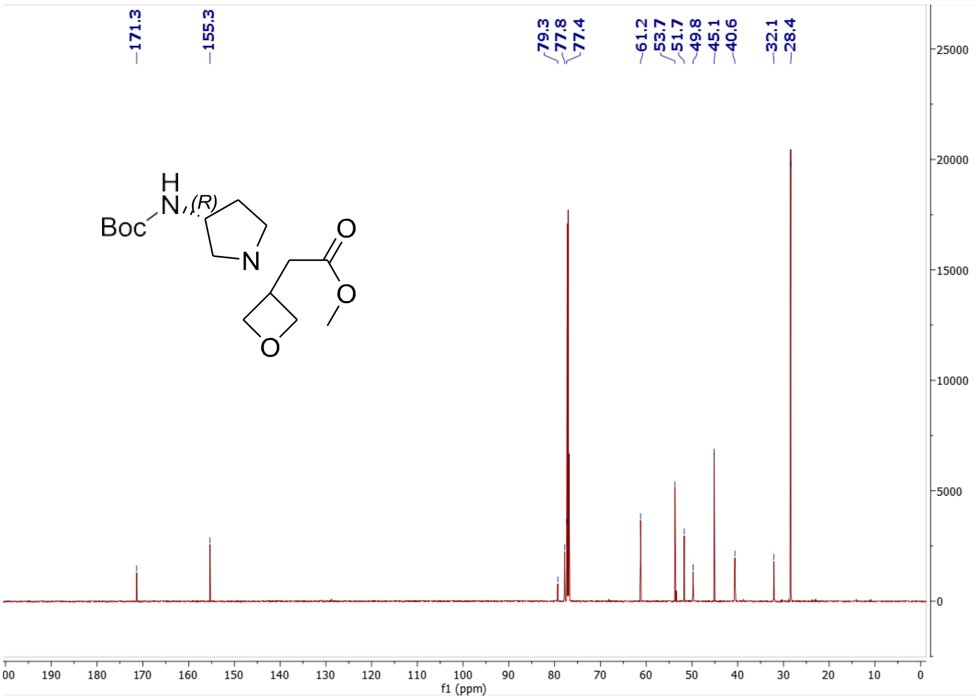

Figure S142. Methyl (3-{(3*R*)-3-[(*tert*-butoxycarbonyl)amino]pyrrolidin-1-yl}oxetan-3-yl)acetate (8c) ^{13}C NMR spectrum (176 MHz, $CDCl_3$).

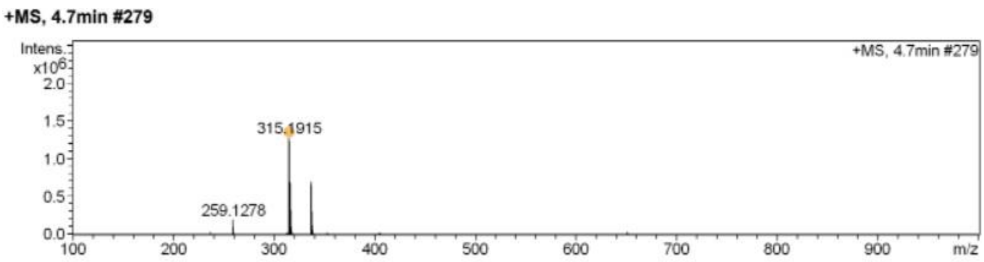

Meas. m/z	#	Ion Formula	m/z	err [ppm]	mSigma	# Sigma	Score	rdb	e⁻ Conf	N-Rule
315.1915	1	C15H27N2O5	315.1914	-0.3	219.5	3	56.17	3.5	even	ok

Figure S143. Methyl (3-{(3*R*)-3-[(*tert*-butoxycarbonyl)amino]pyrrolidin-1-yl}oxetan-3-yl)acetate (8c) HRMS (ESI-TOF).

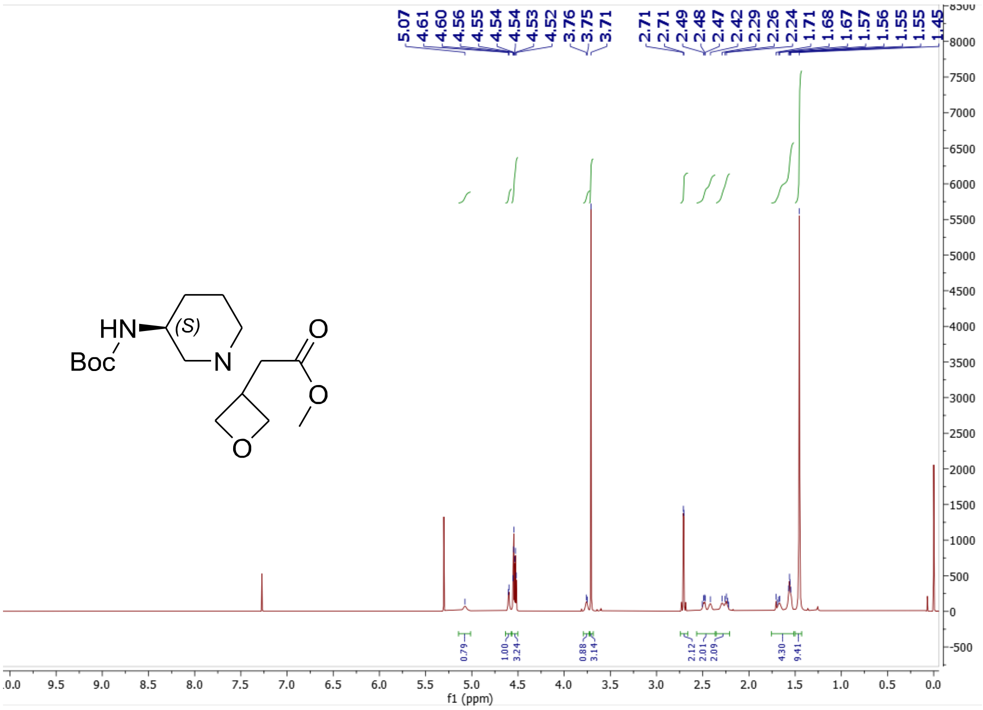

Figure S144. Methyl (3-{(3*S*)-3-[(*tert*-butoxycarbonyl)amino]piperidin-1-yl}oxetan-3-yl)acetate (8d) ^{1}H NMR spectrum (700 MHz, $CDCl_3$).

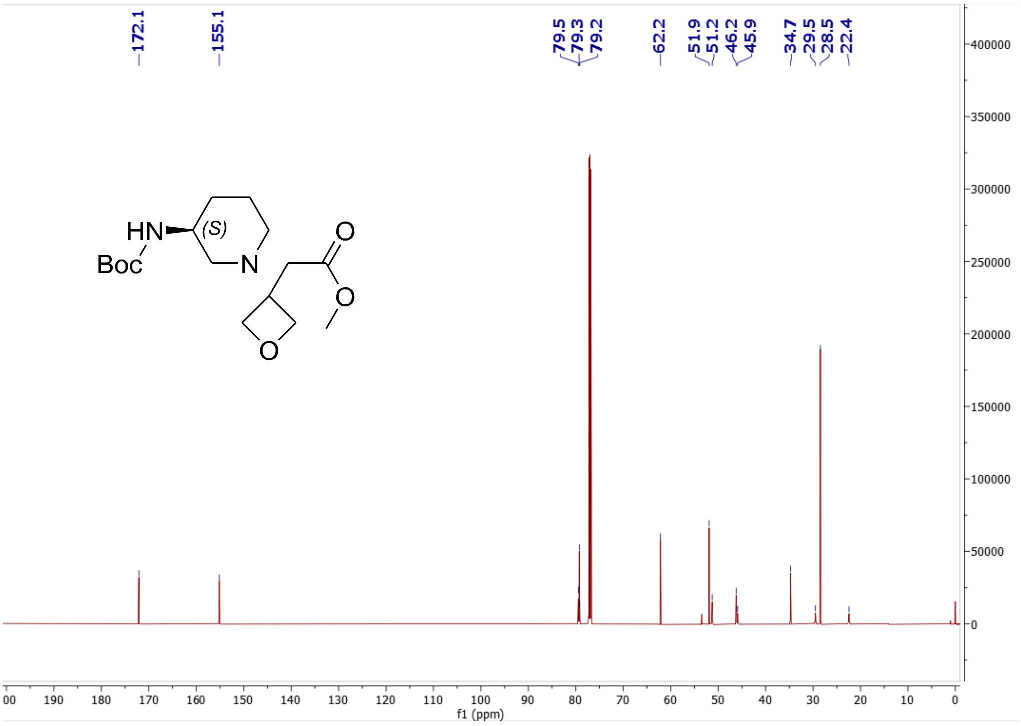

Figure S145. Methyl (3-{(3*S*)-3-[(*tert*-butoxycarbonyl)amino]piperidin-1-yl}oxetan-3-yl)acetate (8d) ^{13}C NMR spectrum (176 MHz, $CDCl_3$).

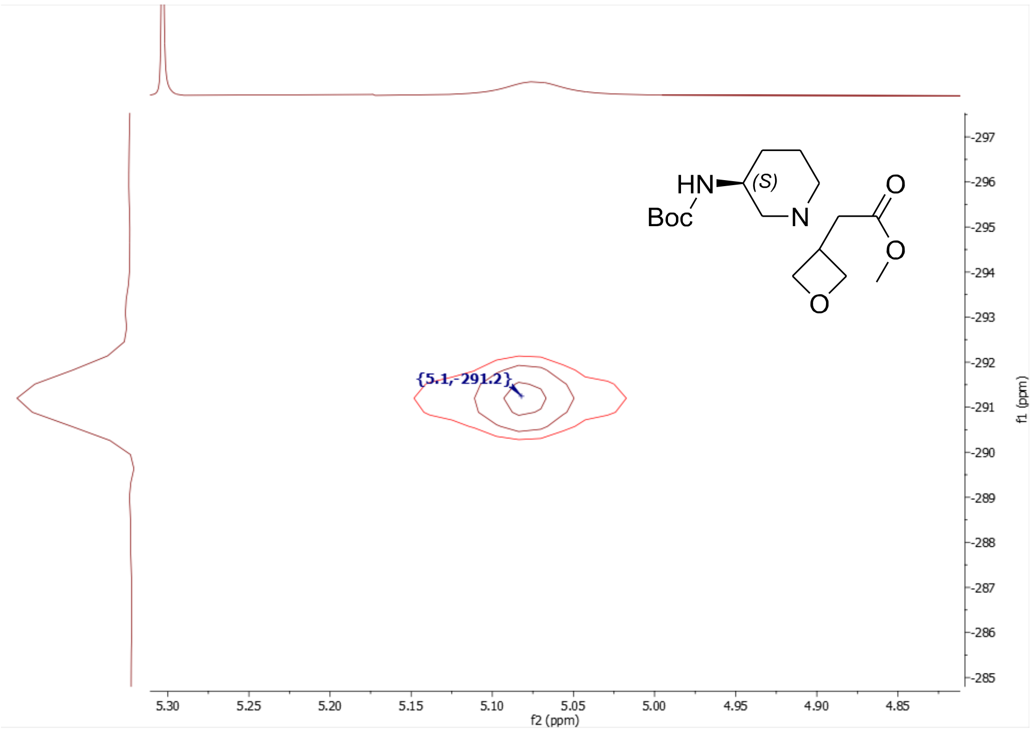

Figure S146. Methyl (3-{(3*S*)-3-[(*tert*-butoxycarbonyl)amino]piperidin-1-yl}oxetan-3-yl)acetate (8d) ^{1}H-^{15}N HSQC spectrum (71 MHz, $CDCl_3$).

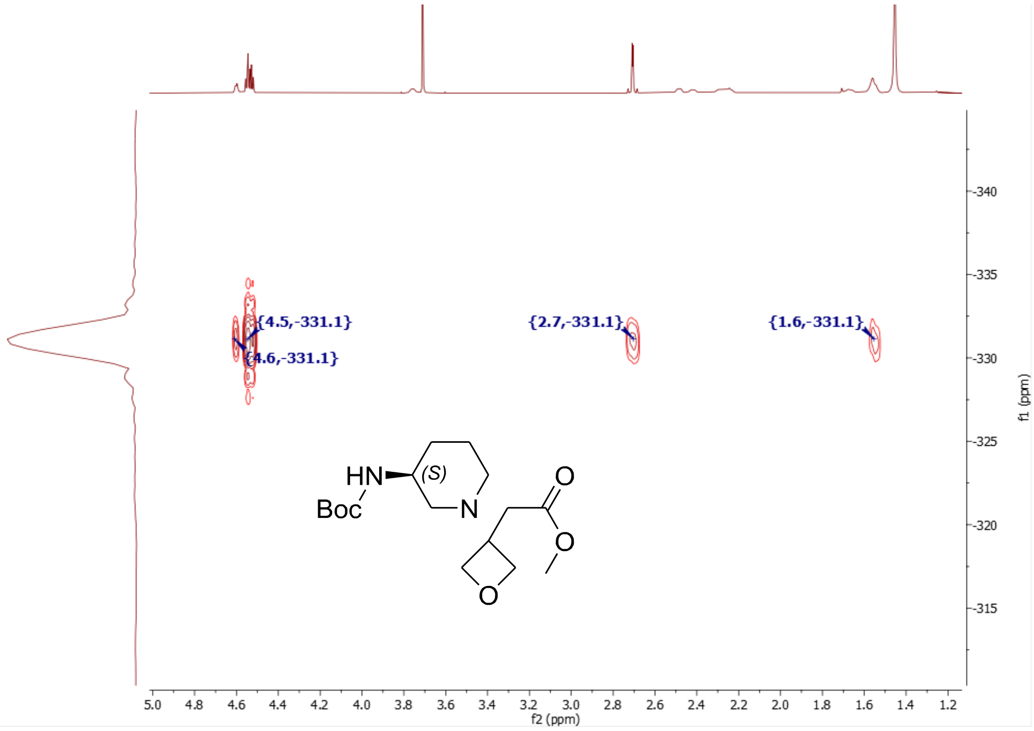

Figure S147. Methyl (3-{(3*S*)-3-[(*tert*-butoxycarbonyl)amino]piperidin-1-yl}oxetan-3-yl)acetate (8d) ^{1}H-^{15}N HMBC spectrum (71 MHz, $CDCl_3$).

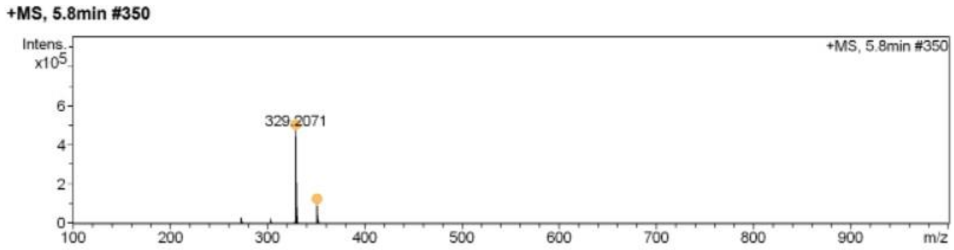

Meas. m/z	#	Ion Formula	m/z	err [ppm]	mSigma	# Sigma	Score	rdb	e⁻ Conf	N-Rule
329.2071	1	C16H29N2O5	329.2071	0.1	1.6	1	100.00	3.5	even	ok
351.1888	1	C16H28N2NaO5	351.1890	-0.8	3.9	1	100.00	3.5	even	ok

Figure S148. Methyl (3-{(3*S*)-3-[(*tert*-butoxycarbonyl)amino]piperidin-1-yl}oxetan-3-yl)acetate (8d) HRMS (ESI-TOF).

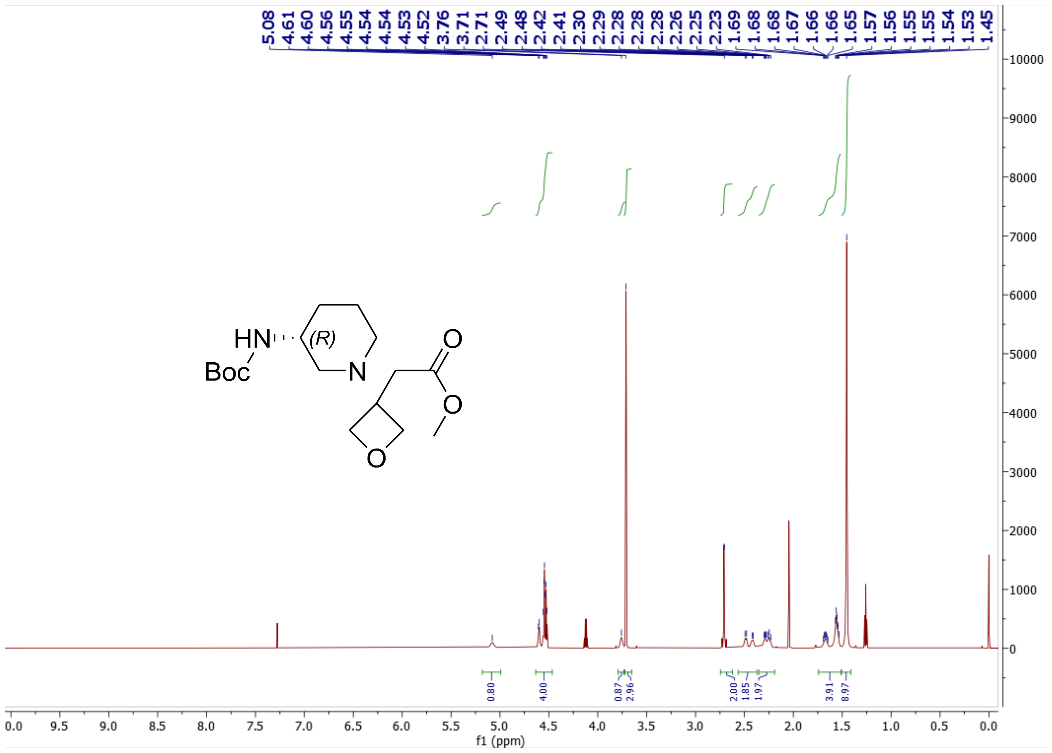

Figure S149. Methyl (3-{(3*R*)-3-[(*tert*-butoxycarbonyl)amino]piperidin-1-yl}oxetan-3-yl)acetate (8e) ^{1}H NMR spectrum (700 MHz, $CDCl_3$).

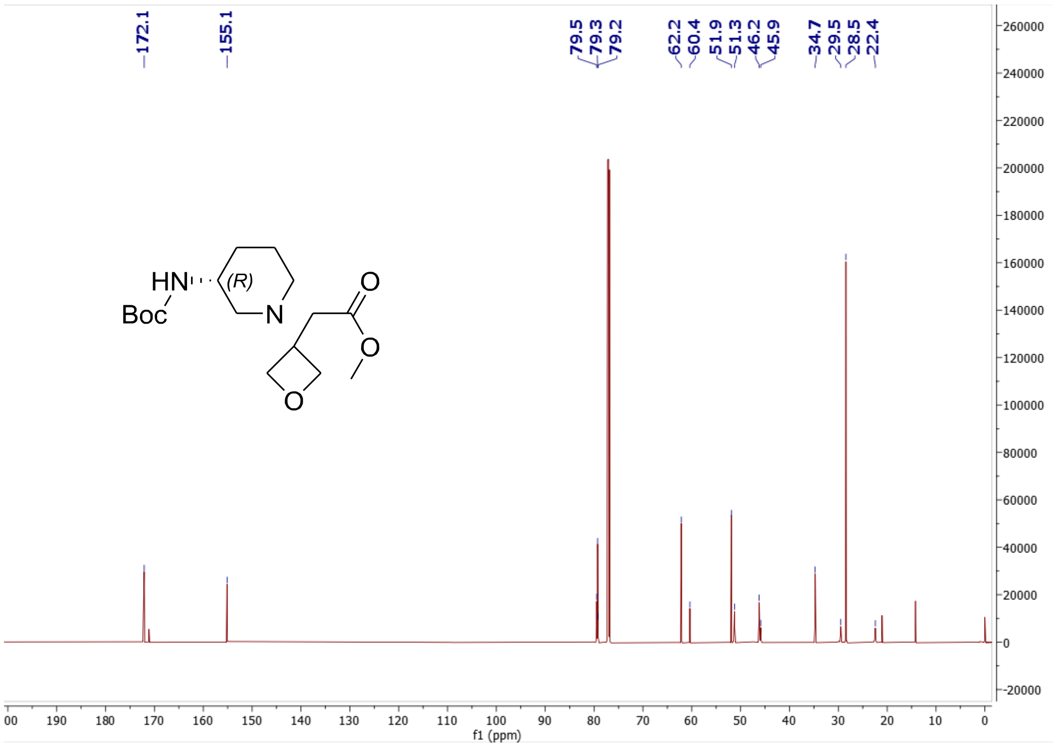

Figure S150. Methyl (3-{(3*R*)-3-[(*tert*-butoxycarbonyl)amino]piperidin-1-yl}oxetan-3-yl)acetate (8e) ^{13}C NMR spectrum (176 MHz, $CDCl_3$).

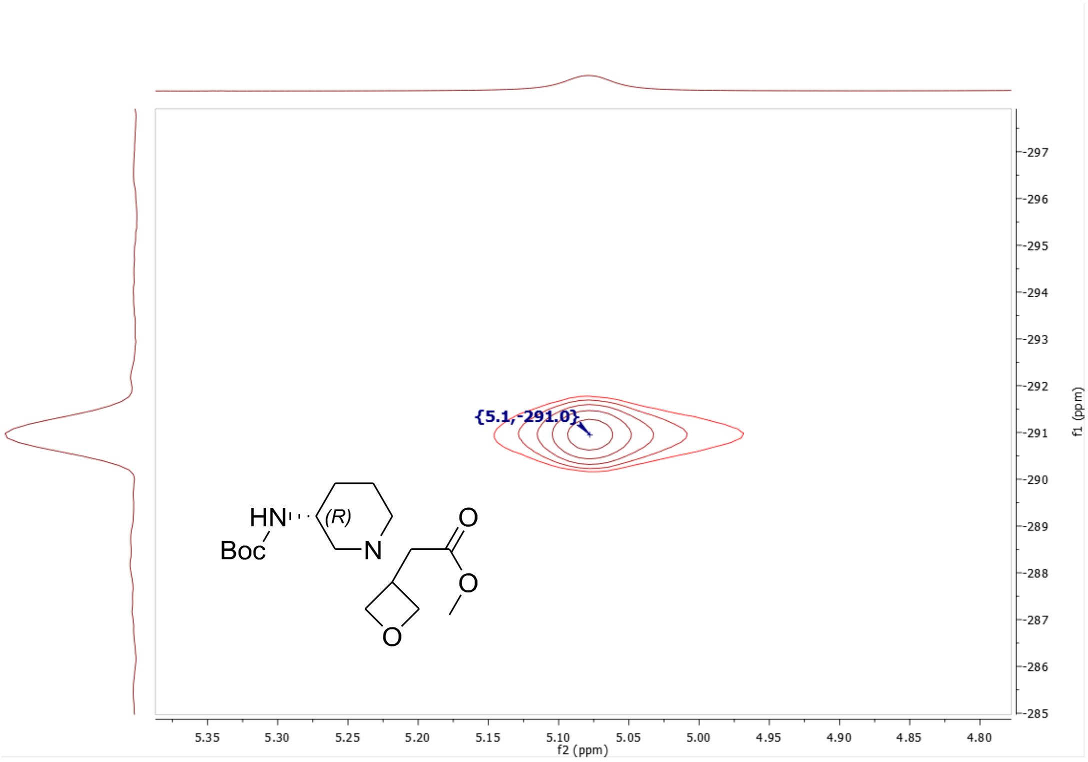

Figure S151. Methyl (3-{(3*R*)-3-[(*tert*-butoxycarbonyl)amino]piperidin-1-yl}oxetan-3-yl)acetate (8e) ^{1}H-^{15}N HSQC spectrum (71 MHz, $CDCl_3$).

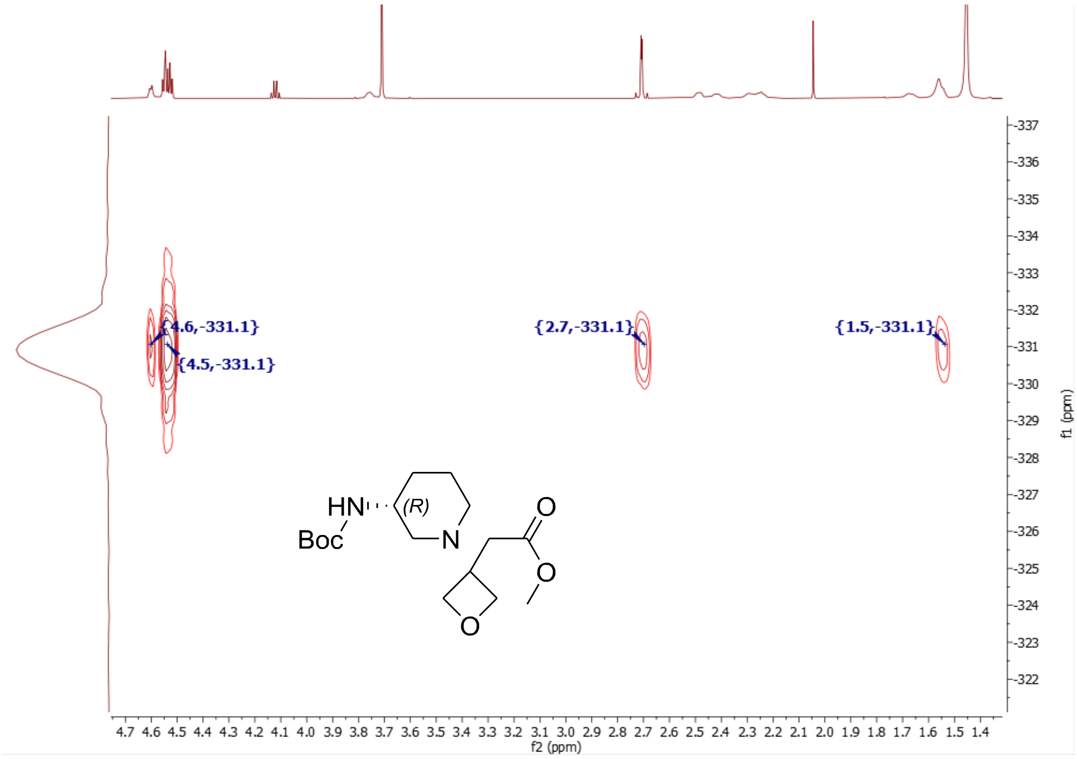

Figure S152. Methyl (3-{(3*R*)-3-[(*tert*-butoxycarbonyl)amino]piperidin-1-yl}oxetan-3-yl)acetate (8e) ^{1}H-^{15}N HMBC spectrum (71 MHz, $CDCl_3$).

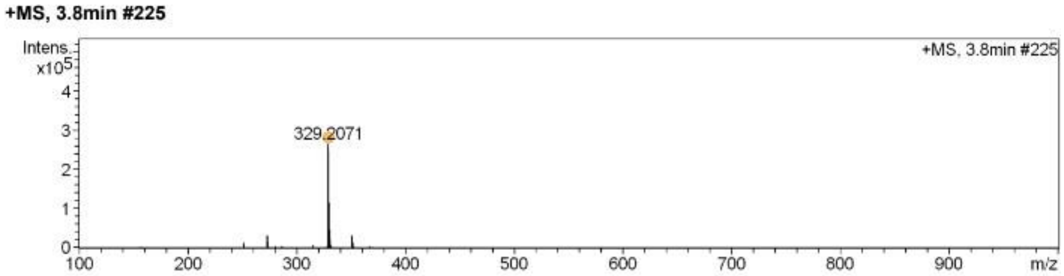

Meas. m/z	#	Ion Formula	m/z	err [ppm]	mSigma	# Sigma	Score	rdb	e⁻ Conf	N-Rule
329.2071	1	C16H29N2O5	329.2071	-0.1	6.3	1	100.00	3.5	even	ok

Figure S153. Methyl (3-{(3*R*)-3-[(*tert*-butoxycarbonyl)amino]piperidin-1-yl}oxetan-3-yl)acetate (8e) HRMS (ESI-TOF).

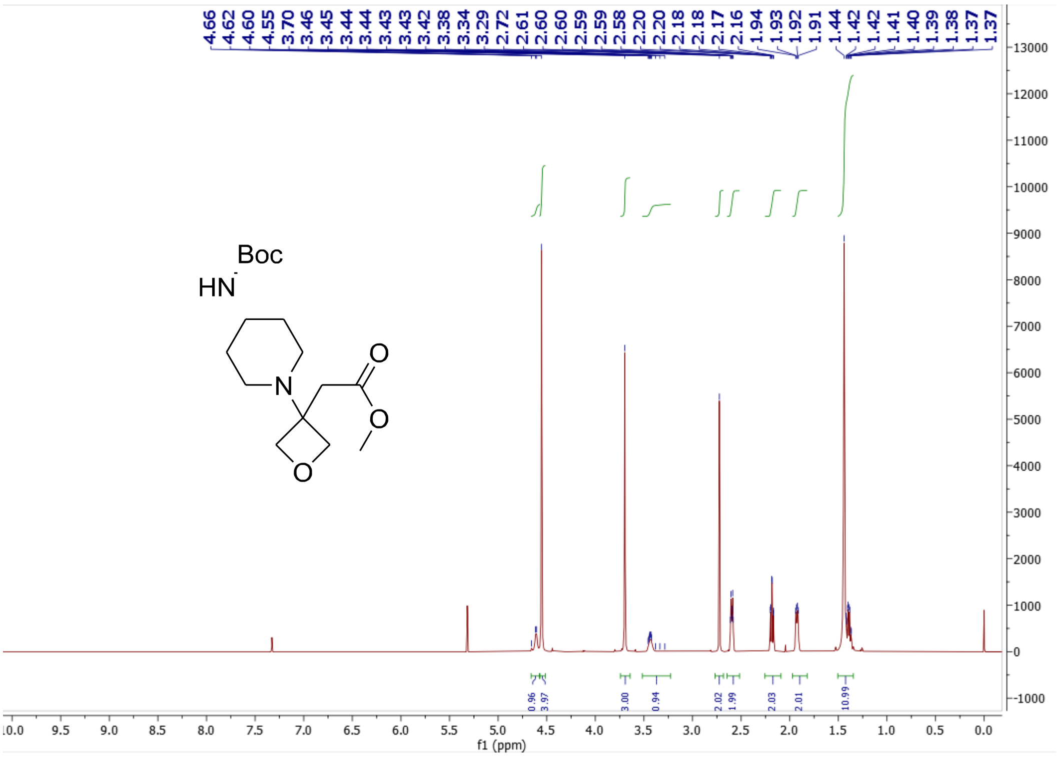

Figure S154. Methyl (3-{4-[(*tert*-butoxycarbonyl)amino]piperidin-1-yl}oxetan-3-yl)acetate (8f) ¹H NMR spectrum (700 MHz, $CDCl_3$).

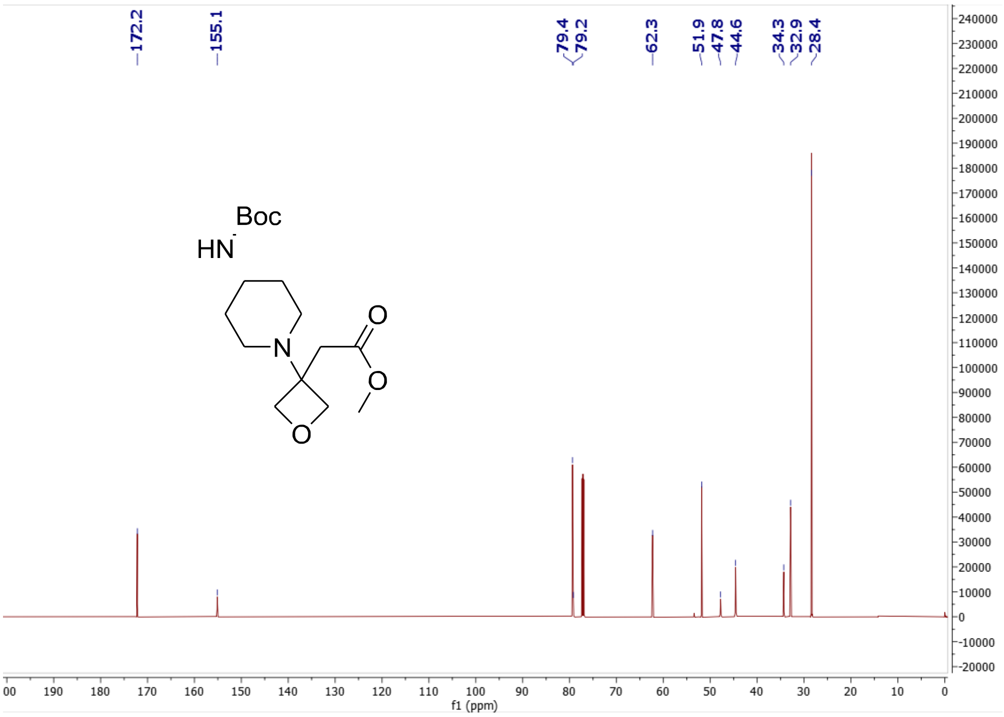

Figure S155. Methyl (3-{4-[(*tert*-butoxycarbonyl)amino]piperidin-1-yl}oxetan-3-yl)acetate (8f) ^{13}C NMR spectrum (176 MHz, $CDCl_3$).

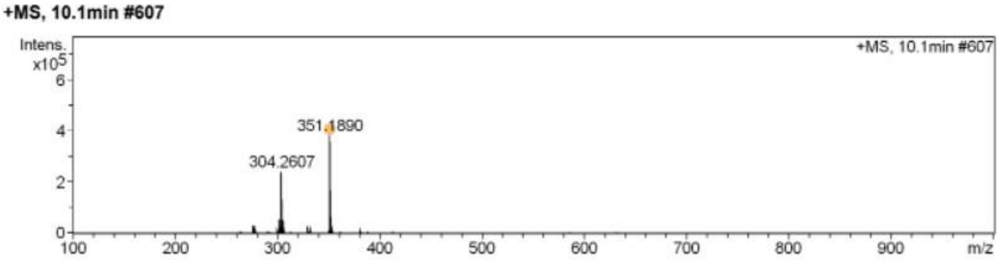

Meas. m/z	#	Ion Formula	m/z	err [ppm]	mSigma	# Sigma	Score	rdb	e⁻ Conf	N-Rule
351.1890	1	C16H28N2NaO5	351.1890	0.1	16.0	1	100.00	3.5	even	ok

Figure S156. Methyl (3-{4-[(*tert*-butoxycarbonyl)amino]piperidin-1-yl}oxetan-3-yl)acetate (8f) HRMS (ESI-TOF).

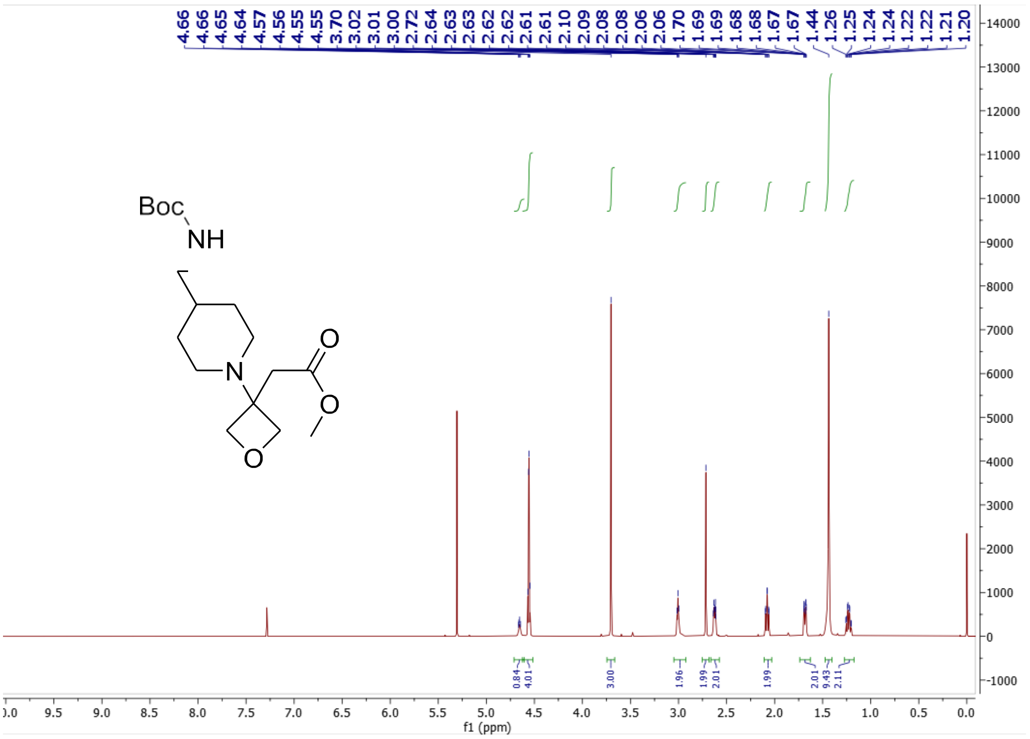

Figure S157. Methyl [3-(4-{[(*tert*-butoxycarbonyl)amino]methyl}piperidin-1-yl)oxetan-3-yl]acetate (8g) ^{1}H NMR spectrum (700 MHz, $CDCl_3$).

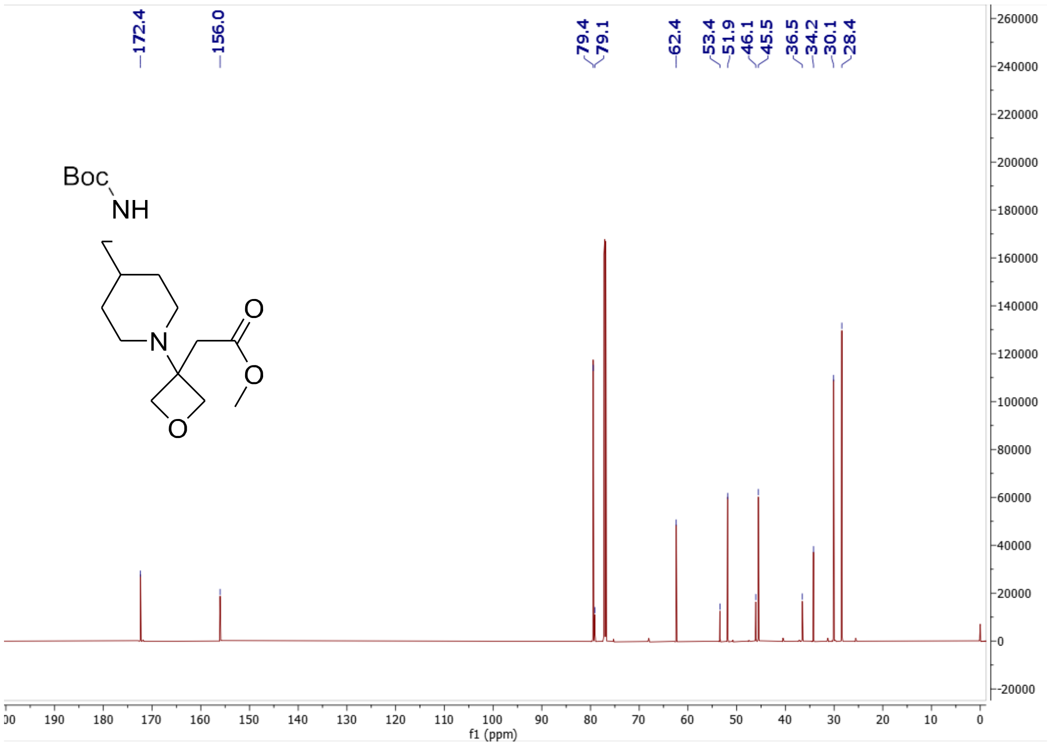

Figure S158. Methyl [3-(4-{[(*tert*-butoxycarbonyl)amino]methyl}piperidin-1-yl)oxetan-3-yl]acetate (8g) ^{13}C NMR spectrum (176 MHz, $CDCl_3$).

+MS, 7.1min #423

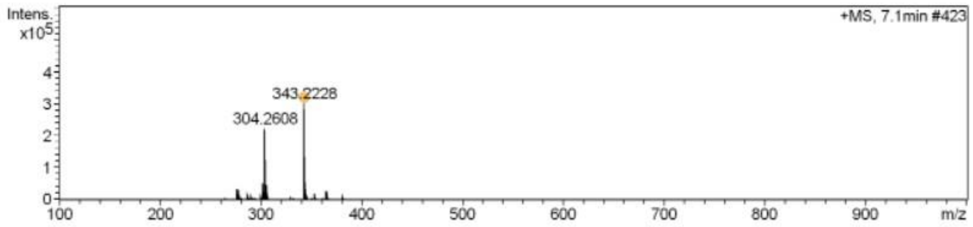

Meas. m/z	#	Ion Formula	m/z	err [ppm]	mSigma	# Sigma	Score	rdb	e⁻ Conf	N-Rule
343.2228	1	C17H31N2O5	343.2227	-0.2	1.4	1	100.00	3.5	even	ok

Figure S159. Methyl [3-(4-{[(*tert*-butoxycarbonyl)amino]methyl}piperidin-1-yl)oxetan-3-yl]acetate (8g) HRMS (ESI-TOF).
